# Use of Fixed Dose Combination (FDC) Drugs in India: Central Regulatory Approval and Sales of FDCs Containing Non-Steroidal Anti-Inflammatory Drugs (NSAIDs), Metformin, or Psychotropic Drugs

**DOI:** 10.1371/journal.pmed.1001826

**Published:** 2015-05-12

**Authors:** Patricia McGettigan, Peter Roderick, Rushikesh Mahajan, Abhay Kadam, Allyson M. Pollock

**Affiliations:** 1 William Harvey Research Institute, Barts and The London School of Medicine and Dentistry, London, United Kingdom; 2 Global Health, Policy and Innovation Unit, Queen Mary University of London, London, United Kingdom; 3 Foundation for Research in Community Health, Mumbai, India; Harvard University, Brigham and Women's Hospital, UNITED STATES

## Abstract

**Background:**

In 2012, an Indian parliamentary committee reported that manufacturing licenses for large numbers of fixed dose combination (FDC) drugs had been issued by state authorities without prior approval of the Central Drugs Standard Control Organization (CDSCO) in violation of rules, and considered that some ambiguity until 1 May 2002 about states’ powers might have contributed. To our knowledge, no systematic enquiry has been undertaken to determine if evidence existed to support these findings. We investigated CDSCO approvals for and availability of oral FDC drugs in four therapeutic areas: analgesia (non-steroidal anti-inflammatory drugs [NSAIDs]), diabetes (metformin), depression/anxiety (anti-depressants/benzodiazepines), and psychosis (anti-psychotics).

**Methods and Findings:**

This was an ecologic study with a time-trend analysis of FDC sales volumes (2007–2012) and a cross-sectional examination of 2011–2012 data to establish the numbers of formulations on the market with and without a record of CDSCO approval (“approved” and “unapproved”), their branded products, and sales volumes. Data from the CDSCO on approved FDC formulations were compared with sales data from PharmaTrac, a database of national drug sales. We determined the proportions of FDC sales volumes (2011–2012) arising from centrally approved and unapproved formulations and from formulations including drugs banned/restricted internationally. We also determined the proportions of centrally approved and unapproved formulations marketed before and after 1 May 2002, when amendments were made to the drug rules. FDC approvals in India, the United Kingdom (UK), and United States of America (US) were compared.

For NSAID FDCs, 124 formulations were marketed, of which 34 (27%) were centrally approved and 90 (73%) were unapproved; metformin: 25 formulations, 20 (80%) approved, five (20%) unapproved; anti-depressants/benzodiazepines: 16 formulations, three (19%) approved, 13 (81%) unapproved; anti-psychotics: ten formulations, three (30%) approved, seven (70%) unapproved. After 1 May 2002, the proportions of approved FDC formulations increased for NSAIDs (26%/28%) and anti-psychotics (0%/38%) and decreased for metformin (100%/75%) and anti-depressants/benzodiazepines (20%/18%), and the overall proportion approved remained similar before and after that date.

FDC formulations gave rise to multiple branded products, ranging from 211 anti-psychotic FDC products from ten formulations to 2,739 NSAID FDC products from 124 formulations. The proportions of FDC sales volumes arising from unapproved formulations were as follows: anti-depressants/benzodiazepines, 69%; anti-psychotics, 43%; NSAIDs, 28%; and metformin, 0.4%. Formulations including drugs banned/restricted internationally comprised over 12% of NSAID FDC sales and 53% of anti-psychotic FDC sales. Across the four therapeutic areas, 14 FDC formulations were approved in the UK and 22 in the US.

**Conclusions:**

There was evidence supporting concerns about FDCs. Metformin excepted, substantial numbers of centrally unapproved formulations for NSAID, anti-depressant/benzodiazepine, and anti-psychotic FDCs were marketed; sales volumes were high. The legal need for central approval of new drugs before manufacture has been in place continuously since 1961, including for FDCs meeting the applicable legal test. Proportions of centrally unapproved formulations after 1 May 2002 did not decrease overall, and no ambiguity was found about states’ licensing powers. Unapproved formulations should be banned immediately, prioritising those withdrawn/banned internationally and undertaking a review of benefits and risks for patients in ceasing or switching to other medicines. Drug laws need to be amended to ensure the safety and effectiveness of medicines marketed in India.

## Abstract 2

Patricia McGettigan and colleagues investigated approval for and availability of oral fixed-dose-combination drugs from four therapeutic areas in India.

## Editors' Summary

**Background:**

Patients who are prescribed several different tablets to treat a single condition or multiple coexisting conditions often find it hard to take all their drugs correctly. For some conditions, clinicians can improve medication compliance by prescribing a fixed dose combination (FDC) product, a drug formulation containing two or more active drugs combined in a fixed ratio of doses that are usually available only in a single dosage form. FDCs, which are sometimes called “polypills,” are particularly useful in situations where both the drug combination and the doses needed to treat patients are standardized and stable, such as in the management of HIV/AIDS. FDCs can also be cheaper to manufacture and easier to distribute than single drug formulations, but they nevertheless have some disadvantages over such formulations. For example, the risks of adverse effects can be compounded by including multiple drugs from the same therapeutic group in a single FDC. Thus, to prevent patients being given unsafe or dangerous formulations, many countries regulate the development and marketing of FDCs.

**Why Was This Study Done?:**

Concerns have been expressed internationally about the regulation of medicinal drugs in India, where thousands of FDCs are available. In response to these concerns, in 2011, an Indian parliamentary standing committee closely examined the Indian national drug regulator—the Central Drugs Standard Control Organization (CDSCO). The committee’s report, released in 2012, highlighted multiple deficiencies in the approval processes of the CDSCO, including a failure to test the efficacy and safety of many of the FDCs available in India, but provided no systematic evidence to support its concerns about FDCs. Here, the researchers undertake a time-trend analysis of the sales volumes of oral FDCs for pain relief (analgesia; formulations of non-steroidal anti-inflammatory drugs, NSAIDs), diabetes (metformin formulations), depression/anxiety (anti-depressant/benzodiazepine formulations), and psychosis (anti-psychotic formulations) in India between 2007 and 2012. In addition, they undertake a cross-sectional examination of data from 2011–2012 to determine the contributions to FDC product numbers and sales volumes of formulations with a known record of CDSCO approval (referred to as “approved”) and those for which such a record could not be found (referred to as “unapproved”).

**What Did the Researchers Do and Find?:**

The researchers obtained information on FDC formulations approved between 1961 and 2013 in each therapeutic area from the CDSCO, and FDC sales data from 2007 to 2012 from PharmaTrac, a database of drug sales in India. Of the 175 FDC formulations marketed in India in the therapeutic areas studied, only 60 (34%) were approved. Although 80% of 25 marketed metformin FDC formulations were approved, only 27% of 124 NSAID FDC formulations, 19% of 16 anti-depressant/benzodiazepine FDC formulations, and 30% of ten anti-psychotic FDC formulations were approved. Over the five years included in the time-trend analysis, FDCs accounted for an increasing proportion of total sales volumes. By 2011–2012, FDCs accounted for more than half of all NSAID and oral anti-diabetic drug sales, and one-third and one-fifth of anti-psychotic and anti-depressant/benzodiazepine sales, respectively. Moreover, in 2011–2012, the proportion of FDC sales volumes arising from unapproved formulations was 43% for anti-psychotics, 69% for anti-depressants/benzodiazapines, 28% for NSAIDs, and 0.4% for metformin; formulations including drugs of which use is banned or restricted internationally accounted for 13.6% and 53% of NSAID and anti-psychotic FDC sales, respectively. While “ambiguity” in the rules prior to 2002 was advanced as a reason for some FDCs having been marketed without a record of central approval, the researchers identified no ambiguity, and in fact, following an amendment to the rules in May 2002 that extended the requirements on approval applicants, new FDCs continued to be marketed without a record of central approval.

**What Do These Findings Mean?:**

Although the accuracy of these findings is likely to be limited by the use of publicly available records of drug approvals and by the use of commercial sales records, the study’s results support concerns about the marketing and use of FDCs in India. They indicate that large numbers of unapproved formulations are available for three of the four therapeutic areas examined, and that sales volumes of these unapproved FDCs (including FDCs that included internationally banned or restricted drugs) are high. The researchers make several recommendations to remedy this situation, which in their view risks harming patients. Sale and manufacture of unapproved FDC formulations, they suggest, should be banned immediately. Withdrawal from the market should be staged, with priority given to removing those formulations containing drugs that are banned or restricted internationally. Manufacturers who wish to retain specific FDCs on the market should be required to submit approval applications to the CDSCO. If this recommendation is implemented, the researchers note, clinicians will need to review the needs of patients taking unapproved FDCs and provide appropriate alternatives. To ensure in the long-term the safety and effectiveness of new medicines marketed in India, as well as transparency of the approval process, the researchers call for amendments in India’s regulatory processes and drug laws. They also suggest that a review should be undertaken of the safety and effectiveness of FDCs currently available in India.

**Additional Information:**

Please access these websites via the online version of this summary at http://dx.doi.org/10.1371/journal.pmed.1001826.

- Wikipedia provides information about fixed dose combination products (note that Wikipedia is a free online encyclopedia that anyone can edit; available in several languages)
- The US Food and Drug Administration regulates all medical drugs and devices in the US and provides information about combination products, including FDCs
- The World Health Organization provides a brief introduction to FDCs and draft guidance for the registration of FDCs
- Information about the CDSCO, the Indian national drug regulatory body, is available; the CDSCO website includes the approved FDC list; the Indian parliamentary standing committee report on the CDSCO is available
- The Medicines and Healthcare Products Regulatory Agency provides information on medicines regulation in the UK
- The European Medicines Agency provides information on medicines regulation in Europe

## Introduction

Drug regulators internationally have expressed concerns about the quality of medicines manufactured in India [1–3]. Nationally, the rigour of the national regulator, the Central Drugs Standard Control Organization (CDSCO), based in the Ministry of Health and Family Welfare, in safeguarding the interests of patients has been questioned in parliamentary and academic reports [4–7]. Particular concerns have been approvals for and the proliferation of fixed dose combination (FDC) products, formulations comprised of two or more drugs combined in a fixed ratio of doses and available in a single dosage form [5,6,8]. Thousands of FDCs are available in India [9]. Many are judged safe and effective and are widely used in situations where both the drug combination and the doses needed are standardised and stable, for example, FDCs for the treatment of human immunodeficiency virus (HIV) or tuberculosis infections, and for contraception. Many others have been judged unsafe, even dangerous, in terms of the combinations of drugs, for example, FDCs comprising multiple drugs from the same therapeutic group (chlorpromazine + trifluoperazine, dual phenothiazine anti-psychotics; mefenamic acid + ibuprofen, dual non-steroidal anti-inflammatory drugs [NSAIDs]) and combinations of centrally acting drugs (chlordiazepoxide + trifluoperazine) [10–12]. In the former, risks of adverse effects are compounded (including central nervous system [CNS] toxicity and cardiac arrhythmia [phenothiazine] and upper gastrointestinal complications [NSAIDs]). In the latter, while both drugs may be indicated, separate dose adjustments cannot be easily undertaken.

In 2007, the CDSCO banned from sale some 294 FDCs because they had never been approved by the central regulator but had been granted manufacturing/distribution licenses by state drug authorities; the list was published in 2014 [13,14]. FDC manufacturers disputed the ban and obtained a stay from the Madras High Court [13]. The matter remains unresolved. Concerns about regulatory capacity and rigour led to an examination in 2011 of the CDSCO by the Department-Related Parliamentary Standing Committee on Health and Family Welfare, one of over 20 standing committees consisting of members of Parliament and established by the Parliament of India to undertake specialist parliamentary work [6]. In 2012, it issued its report (the 59th Report) [6]. This report highlighted multiple deficiencies in the approval processes of the CDSCO, noting that these were compounded by understaffing, a lack of appropriate skills, and inadequate infrastructure. In respect of FDCs, the committee reported that the issue of manufacturing licenses being granted by some states for FDCs without prior CDSCO approval had been “first deliberated” by the Drugs Technical Advisory Board in 2000, and the committee expressed concern that “even after a lapse of a decade, no serious action has been taken”, and stated the following:

Unfortunately some State Drug Authorities have issued manufacturing licenses for a very large number of FDCs without prior clearance from CDSCO. This is in violation of rules though till May 2002, there was some ambiguity on powers of the State Drug Authorities in this respect. However the end result is that many FDCs in the market have not been tested for efficacy and safety. This can put patients at risk. (Section 9 of [6])

The committee also noted that multiple FDCs available in India had been rejected by regulators in Europe, North America, and Australia, while others never had approval applications submitted outside India (Section 7 of [6]). The report, however, provided no systematic examination of regulatory or drug utilisation data to support its complaints.

### Concern with FDCs

To our knowledge, this is the first empiric study into the concerns described about FDCs by the 59th Report. It examines the regulation and use in India of oral FDCs in four therapeutic areas: analgesia (NSAIDs), diabetes (metformin), anxiety/depression (benzodiazepines/anti-depressants), and psychosis (anti-psychotics). We chose these areas because the drugs are commonly used and/or are associated with considerable potential for interactions and adverse events. Examples of both NSAID and anti-psychotic FDCs were highlighted in the 59th Report.

### India’s FDC Regulatory Framework

The much-amended Drugs and Cosmetics Act, 1940, and Drugs and Cosmetics Rules, 1945, govern the regulation of drugs [15] (S1 and S2 Texts). The 1940 law, passed under British colonial rule, placed responsibility for imports on central government, with the states being responsible for manufacture, distribution, and sale. Following independence in 1947 and subsequent adoption of the Constitution, “drugs” became a matter contained in the “Concurrent List” so that both the national Parliament and the state legislatures had, and have, power to make laws in relation to them. In 1952, national rules introduced the concept of a “new drug” along with the requirement for prior central approval for import (S3 Text). This was followed in 1961 by the requirement for prior central approval for manufacture, along with an obligation on state license applicants to produce evidence that the drug had been approved (S4 and S5 Texts). FDCs were not specifically mentioned, but they were regarded as new drugs, with recorded central approvals for FDC formulations dating (continuously) from 1961.

Increased central control of drug regulation has occurred incrementally ever since, whilst the states have retained their licensing powers over the manufacturing and sale of most drugs. A 1988 amendment inserted a new Part XA into the national rules entitled “Import or manufacture of new drugs for clinical trials or marketing” (S6 Text). Part XA included (and includes) requirements for pre-manufacturing central approval before a state manufacturing license is granted and for license applicants to produce evidence of that approval, whilst expressly including FDCs in the definition of a new drug and setting out specific data submission requirements for FDCs. After September 1988, FDCs combining drugs for the first time that had been individually approved previously, or previously combined FDCs with new claims, were expressly included within the definition of a “new drug” under Rule 122E(c). Those FDCs therefore required central approval prior to manufacturing under Rules 122B or 122C, and applicants had to submit evidence to state authorities of that prior approval. This is reflected in the heading of Rule 122D: “Application for permission to import or manufacture fixed dose combination of drugs”.

In 2001, the rules were amended again to impose the legal duty on the CDSCO to be satisfied when approving new drugs for import or manufacture that they are safe and effective. The duty was imposed for FDCs as well, with the amendment further stating that FDCs needed prior approval even though they fell within the definition of new drugs and so were covered as far as the “safe and effective duty” was concerned, whilst the post-1961 provisions and the 1988 amendments covered them as far as the requirement for prior central approval was concerned. An amendment in May 2002, inserting Rules 69(6) and 75(6), essentially duplicated the requirement to produce evidence of prior approval of “new drugs” that had been in the rules since 1961, and extended it to require evidence of approval in favour of the applicant. The 59th Report (Section 9.2 of [6]) noted “some ambiguity” until May 2002. We identified no ambiguity in the rules. Our detailed analysis of the rules (S1 Text) leads us to consider that an FDC needed prior central approval for manufacture—and the submission to states of evidence of that approval—from 1961 if it fell within the three different definitions of a “new drug” applying from 1961–1988, 1988–1999, and 1999 onwards (see Box 1). Rules 69(6) and 75(6) are not relevant to determining that question, but they imposed an additional requirement of producing evidence of approval in favour of the applicant.

**Box 1. Chronology and Detail of Requirements for Central Approval for Import and Manufacture of Fixed Dose Combinations**

**1961–1988**

(1) Prior central approval for manufacture was required for an FDC after June 1961, until September 1988, if either:

- the composition of the FDC in question was not at the time generally recognised among experts as safe for use under the conditions recommended or suggested in the label; or
- the composition of the FDC, as a result of investigations for determining its safety for use under such conditions, was so recognised, but it had not, otherwise than during the course of such investigations, been used to any large extent or for any appreciable length of time under those conditions,

and those applying to a state for a licence to manufacture an FDC or its preparations which fell within (a) or (b) were required to produce along with their application evidence that the drug had already been centrally approved.

**1988–1999**

(2) Between September 1988 and August 1999, prior central approval for import and manufacture of an FDC was legally required if:

- the drugs being combined (i) had been individually approved earlier for certain claims, and (ii) it is proposed to combine them for the first time in a fixed ratio; or
- the drugs being combined (i) had been individually approved earlier for certain claims, and (ii) it is proposed to change the ratio, indications, dosage, dosage form, or route of administration; or
- of the drugs being combined (i) one or more of them had not been previously approved, and (ii) (at least) the combination (and, possibly, each of the individually unapproved drugs) constituted a new substance of chemical, biological, or biotechnological origin in bulk or prepared dosage form used for prevention, diagnosis, or treatment of disease in man or animal; which, except during local clinical trials, has not been used in the country to any significant extent; and which, except during local clinical trials, has not been recognised in the country as effective and safe for the proposed claims,

and those applying to a state had to produce evidence that the drug had already been centrally approved. The requirement in effect lapsed after 4 y from either the date of the first approval or, if earlier, from the date of the inclusion of the FDC in the Indian Pharmacopoeia.

**Since August 1999**

(3) Since August 1999, the position has been the same as under (2) above, but the criteria in (c)(ii) have changed, so that since August 1999 (c) would read:

of the drugs being combined (i) one or more of them has not been previously approved, and (ii) (at least) the combination (and, possibly, each of the individually unapproved drugs) constitutes a drug within the definition of the Act (see S1 Text) including drug bulk substance—if it has not been used significantly in India (and if there has been any limited use, such use must have been with the permission of the central licensing authority) under the conditions prescribed, recommended, or suggested in its label, and has not been recognised by the central authority as effective and safe for the proposed claims (S7 Text).

Further amendments in 2005 removed references to minimum numbers or ranges of participants and sites in “new drug” clinical trials and gave the CDSCO discretion to override data submission rules. For 4 y after approval, or after inclusion in the Indian Pharmacopoeia if earlier, companies wanting to market new drugs—including FDCs—must obtain approval of their own formulation from the CDSCO. After 4 y, new drugs cease to be deemed “new” drugs, and applications for manufacturing/distribution licences can be made to state licensing authorities without prior CDSCO approval.

### Objectives of This Study

We aimed to determine if evidence existed to support the concerns of the 59th Report. Our objectives were to (i) determine the numbers of approvals granted by the CDSCO (1961–2013) for oral FDC formulations in the four therapeutic areas of interest; (ii) determine for each therapeutic area the numbers of FDC formulations on the market (2011–2012), their CDSCO approval status, the numbers of branded products marketed, and the relative contributions to FDC sales (2011–2012) of formulations with and without a record of CDSCO approval (“approved” and “unapproved”); and (iii) evaluate the impact of the May 2002 amendment to the rules by determining the proportions of new formulations launched on the market before and after 1 May 2002 that had CDSCO approval, the numbers of products arising, and their sales volumes (2011–2012).

Finally, we wished to determine if FDC formulations available in India were approved by United Kingdom (UK) and/or United States of America (US) regulators or included drugs banned, restricted, or unapproved internationally, and to apply our findings to make recommendations for rationalising the regulation of, and hence the use of, FDCs in India.

## Methods

This was an ecologic study of FDC approvals in India. It included a time-trend analysis of FDC sales volumes (2007–2012) in each of the four therapeutic areas of study, with a cross-sectional examination of the 2011–2012 data to determine the contributions to FDC product numbers and sales volumes of CDSCO-approved and-unapproved formulations.

### FDC Approvals in India

Using publicly accessible records available from the CDSCO for the period 1961–2013, we collated information on FDC approvals granted annually in each area [16]. The CDSCO listed approvals chronologically in a portable document format (pdf) that included the drugs comprising individual FDC formulations, indication, and the date of approval. Relevant information was extracted into an Excel spreadsheet. We focussed on original formulation approval, that is, the first approval granted for the drug combination in the FDC being examined. We categorised a formulation as “approved” if the combination of drugs, irrespective of dose amounts or modified release variations, was ever recorded as approved by the CDSCO. We categorised a formulation as “unapproved” if it was not included in the list of CDSCO approvals, 1961–2013 (Box 2). We assumed the CDSCO approval records were complete. No information was available publicly on the clinical evidence that was provided to support approvals. State drug authority records of FDC manufacturing/distribution/sale licences were unavailable, but from the list of 294 FDCs banned by the CDSCO in 2007, we identified FDCs in the study categories that had state licenses only [14].

**Box 2. Glossary**

**“Approved”**

This single term is used in the paper to encompass the prior action required by the CDSCO before a state licensing authority can give a license for manufacture/sale/distribution of a new drug. In the Indian legal documents, the terms used are as follows: the CDSCO gives “permission” for import of new drugs, must “approve” manufacture of new drugs, and gives “permission” for the import and manufacture of new drugs, including FDCs.

**“Unapproved”**

This term is used in the paper to encompass FDC formulations for which we found no record of CDSCO approval. We assumed CDSCO records were complete.

**Drug**

A clinically active component in a formulation.

**Drugs Technical Advisory Board**

The board established under Section 5 of the Drugs and Cosmetics Act, 1940, to advise the central and state governments on technical matters arising out of the administration of the act.

**Formulation**

The drugs combined together to make an FDC product.

**Product**

The finished FDC as manufactured and named (or branded) by a pharmaceutical company. Multiple companies may choose to manufacture FDCs of the same formulation. FDCs made by different pharmaceutical companies are given brand names to distinguish them from FDCs of the same formulation made by other companies.

**State licensing authority**

The state-based authority responsible for manufacture, distribution, and sale of drugs; drugs are required to have a state license before they are marketed.

### FDC Approvals in the UK and US

To determine approvals in the UK and US, we searched the Medicines and Healthcare Products Regulatory Agency (MHRA) and Food and Drug Administration (FDA) approval listings for 2014 [17,18]. The FDA index (the Orange Book) lists all approved FDCs and single drug formulations (SDFs) alphabetically by generic name [18]. The MHRA publishes no index of generic name FDC approvals, and its list of approvals does not include medicines licensed centrally by the European Medicines Agency [17], so to minimise the risk of overlooking FDCs approved for use in the UK, we also examined listings in the British National Formulary and in the Monthly Index of Medical Specialties (MIMS) [12,19].

### FDC Sales in India

We determined for each therapeutic area the numbers of FDC formulations and branded products marketed, and we undertook a time-trend analysis of annual sales volumes over 5 y, 2007–2012. Data were obtained from PharmaTrac, a commercial database comprising monthly audits of pharmaceutical product sales through multiple supply routes (some 5,000 pharmaceutical companies, 18,000 distributers and stockists, and 32,000 sub-stockists) to over 500,000 retailers, hospitals, and dispensing doctors in 23 regions of India [20]. The audits capture 35% of national sales. The sampling data are projected to estimate national sales totals. The data largely represent private prescription sales; 85% are based on retailer sales, and 15% on drugs supplied through hospitals and dispensing doctors. They include generic and branded product names, manufacturers, sales volumes and value, and date of market launch. Each formulation has a code that identifies it in the database according to therapy type and treatment group, so, for example, diclofenac + paracetamol is M1A14, a musculoskeletal therapy in the anti-rheumatic systemic non-steroidal group; metformin + gliclazide is A10B10, an alimentary tract and metabolism therapy in the oral anti-diabetic group. Sales volumes are reported as the numbers of units of ten tablet/capsule doses; we converted units to the numbers of tablets/capsules. Value is reported in rupees; we used current exchange rates to report in US dollars. We examined monthly data available for the 5-y period November 2007–October 2012. We adhered to SAMPL guidelines for reporting [21].

There was a large amount of data; in presenting the results, we focus on the most recent 12-mo period, November 2011–October 2012. For each therapeutic area, we categorised sales according to whether FDC formulations had a record of approval by the CDSCO. Taking into account the 59th Report note on “ambiguity” in the national rules (Section 9.2 of [6]) until 1 May 2002 regarding the powers of state drug authorities to grant manufacturing licenses for FDC formulations that did not have prior CDSCO approval, we also examined CDSCO approvals for FDC formulations marketed before and after 1 May 2002, anticipating that all new formulations marketed after 1 May 2002 would have CDSCO approval. (S1 Table includes the individual formulation and product data for each of the 5 y examined.)

#### Data extraction

We searched the PharmaTrac database according to therapy and treatment group in the first instance. For each therapeutic area, we identified the FDC formulations and extracted into Excel worksheets the product, volume, and value data. We summed the monthly data to obtain 12-mo volumes and values for the 5-y period November 2007–October 2012. The database was additionally searched using the individual drug names of NSAIDs, oral anti-diabetic drugs, anti-depressants, benzodiazepines, and anti-psychotics. This ensured that relevant FDCs were not overlooked; for example, alprazolam + propranolol was categorised in the database as a cardiac formulation, and naproxen + sumatriptan as a CNS therapy in the anti-migraine group. Searches were conducted in duplicate, with discrepancies resolved by re-searching and agreement.

## Results

### FDC Formulations: CDSCO Approvals and Formulations Marketed and Approved in India, the UK, and the US

#### CDSCO approvals for FDC formulations

Between 1961 and 2013, the CDSCO approved some 1,125 oral FDC formulations including 67 NSAID FCD formulations (of which 52 were original formulations and 15 were variants where, for example, the dose of one component was changed or a modified release dose form used), 41 metformin FDC formulations (21 original, 20 variants), nine original benzodiazepine/anti-depressant formulations, and five original anti-psychotic drug formulations (Table 1). While some older formulations had become obsolete by 2011–2012 (e.g., indomethacin + dexamethasone, approved November 1971; trifluoperazine + analgin + ergotamine + caffeine, approved March 1976), 60 of these 87 approved original formulations were available on the market in 2011–2012.

**Table 1:** Formulations, CDSCO approvals, products, and sales of NSAID, metformin, anti-depressant/benzodiazepine, and anti-psychotic FDCs.

FDC Formulations, Branded Products, and Sales 2011–2012	Therapeutic Area			
	NSAID	Metformin	Anti-Depressant/Benzodiazepine	Anti-Psychotic
Formulations				
All FDC original formulations approved by the CDSCO 1961–2013	52	21	9	5
FDC original formulations available on the market 2011–2012	124	25	16	10
FDC formulations approved/unapproved by the CDSCO (percent formulations approved/unapproved)	34/90 (27%/73%)	20/5 (80%/20%)	3/13 (19%/81%)	3/7 (30%/70%)
FDC formulations marketed before/after 1 May 2002	19/105	5/20	5/11	2/8
FDC formulations marketed before 1 May 2002 and approved by the CDSCO (percent)	5 of 19 (26%)	5 of 5 (100%)	1 of 5 (20%)	0 of 2 (0%)
FDC formulations marketed after 1 May 2002 and approved by the CDSCO (percent)	29 of 105 (28%)	15 of 20 (75%)	2 of 11 (18%)	3 of 8 (38%)
FDC formulations available in the UK	6	8	0	0
FDC formulations available in the US	10	10	1	1
**Branded Products**				
Total number of FDC branded products^a^ on the market 2011–2012	2739	536	301	211
Proportion of FDC branded products arising from formulations approved/unapproved by the CDSCO	57%/43%	98%/2%	24%/76%	22%/78%
Number of FDC branded products arising from formulations marketed before 1 May 2002 (proportions from CDSCO-approved/unapproved formulations)	1,895 (69%/31%)	232 (100%/0%)	171 (13%/87%)	102 (0%/100%)
Number of FDC branded products arising from formulations marketed after 1 May 2002 (proportions from CDSCO-approved/unapproved formulations)	844 (31%/69%)	304 (91%/9%)	130 (38%/62%)	109 (43%/57%)
**Sales 2011–2012**				
Total oral drug sales volume, 2011–2012, FDC plus SDF, millions of tablets/capsules	11,814.2	8,171.4^b^	3,561.1	1,157.8
FDC sales volume, 2011–2012 (percent of total oral drug sales)	7,328.8 (62%)	4,557.8 (56%)^c^	638.5 (18%)	419.3 (33%)
Proportion of FDC sales volume^a^ arising from formulations approved/unapproved by the CDSCO	69%/28%	99.6%/0.4%	31%/69%	57%/43%
FDC sales volume^a^ in 2011–2012 arising from formulations marketed before 1 May 2002 (proportions from formulations approved/unapproved by the CDSCO)	5,397.3 (86%/14%)	1,765.3 (100%/0%)	404.9 (6%/94%)	86.8 (0%/100%)
FDC sales volume^a^ in 2011–2012 arising from formulations marketed after 1 May 2002 (proportions from formulations approved/unapproved by the CDSCO)	1,743.8 (26%/74%)	2,789.1 (99%/1%)	23.4 (75%/25%)	332.5 (71%/29%)
FDC sales value, 2011–2012, millions of Indian rupees (millions of US dollars)^d^	INR 18,223.9 (US$292.63)	INR 20,115.3 (US$322.99)	INR 2,004.8 (US$32.35)	INR 1,276.8 (US$20.60)
FDC sales value proportion arising from formulations approved/unapproved by the CDSCO	39%/61%	0.3%/99.7%	43%/57%	94%/6%

Numbers of FDC formulations of NSAIDs, metformin, benzodiazepines/anti-depressants, and anti-psychotics approved by the CDSCO, total formulations on the market, CDSCO-approved/unapproved formulations, formulations marketed before and after 1 May 2002, and numbers of FDC formulations available in the UK and US; numbers of FDC branded products on the market, proportions arising from approved/unapproved formulations overall, and before/after 1 May 2002; total oral drug sales in each therapeutic area, FDC sales volumes and FDC proportions of total oral drug sales, FDC sales volumes and proportions arising from approved/napproved formulations overall and before/after 1 May 2002, and FDC sales value and proportions arising from approved/unapproved formulations. FDC formulations on the market with and without a record of CDSCO approval are termed respectively “approved” and “unapproved”. Data sources: PharmaTrac; CDSCO-published list of FDC approvals 1961–February 2013.

^a^Excludes products categorised as “other”.

^b^Includes all oral diabetes drugs.

^c^Percent of all oral drug sales for diabetes.

^d^Conversion rate: INR 1 = US$0.016057.

In each therapeutic area, more new (original) formulations were launched on the market after 1 May 2002 than before (Table 1). Overall, the proportions of formulations with CDSCO approval after 1 May 2002 were unchanged compared with before 1 May 2002. The proportions of formulations with CDSCO approval increased for NSAID FDCs (26% to 28%) and anti-psychotic FDCs (0% to 38%) and decreased for metformin FDCs (100% to 75%) and anti-depressant/benzodiazepine FDCs (20% to 18%) (Table 1).

With the exception of the NSAID FDC aspirin + caffeine, none of the FDCs on the market in the therapeutic areas examined were included in the Indian Pharmacopoeia [22].

#### FDC formulations marketed and approved in India, the UK, and the US

There were 124 NSAID formulations on the market in India (2011–2012), of which 34 (27%) were approved in India, six in the UK, and ten in the US (Table 1); 25 metformin FDC formulations in India, of which 20 were approved in India (80%), eight in the UK, and ten in the US; 16 anti-depressant/benzodiazepine FDC formulations in India, of which three (19%) were approved in India, none in the UK, and one in the US; and ten anti-psychotic FDC formulations in India, of which three (30%) were approved in India, none in the UK, and one in the US. All the formulations available in the UK and US in each therapeutic area were approved by the respective regulators. Formulations for which there was no record of CDSCO approval continued to be marketed after 1 May 2002; less than half of NSAID, anti-depressant/benzodiazepine, and anti-psychotic FDC formulations and 75% of metformin FDC formulations marketed after that date had records of CDSCO approval.

Of NSAID FDC formulations, 2/6 approved in the UK and 7/10 approved in the US were not found in India (UK: ibuprofen + phenylephrine, ketoprofen + omeprazole; US: ibuprofen + diphenhydramine, ibuprofen + famotidine, ibuprofen + hydrocodone, ibuprofen + oxycodone, ibuprofen + phenylephrine, naproxen + esomeprazole, naproxen + pseudoephedrine). Of metformin FDC formulations, two were recently approved in the UK and the US and were not found in India (metformin + alogliptin [2013], metformin + canagliflozin [2014]). One anti-depressant/benzodiazepine FDC approved in the US (amitriptyline + chlordiazepoxide) was also available in India but was not included in the CDSCO’s list of approved FDCs. One anti-psychotic FDC approved in the US (amitriptyline + perphenazine) was unavailable in both the UK and India.

### FDC Products Arising from Unapproved Formulations

**Fig 1:**
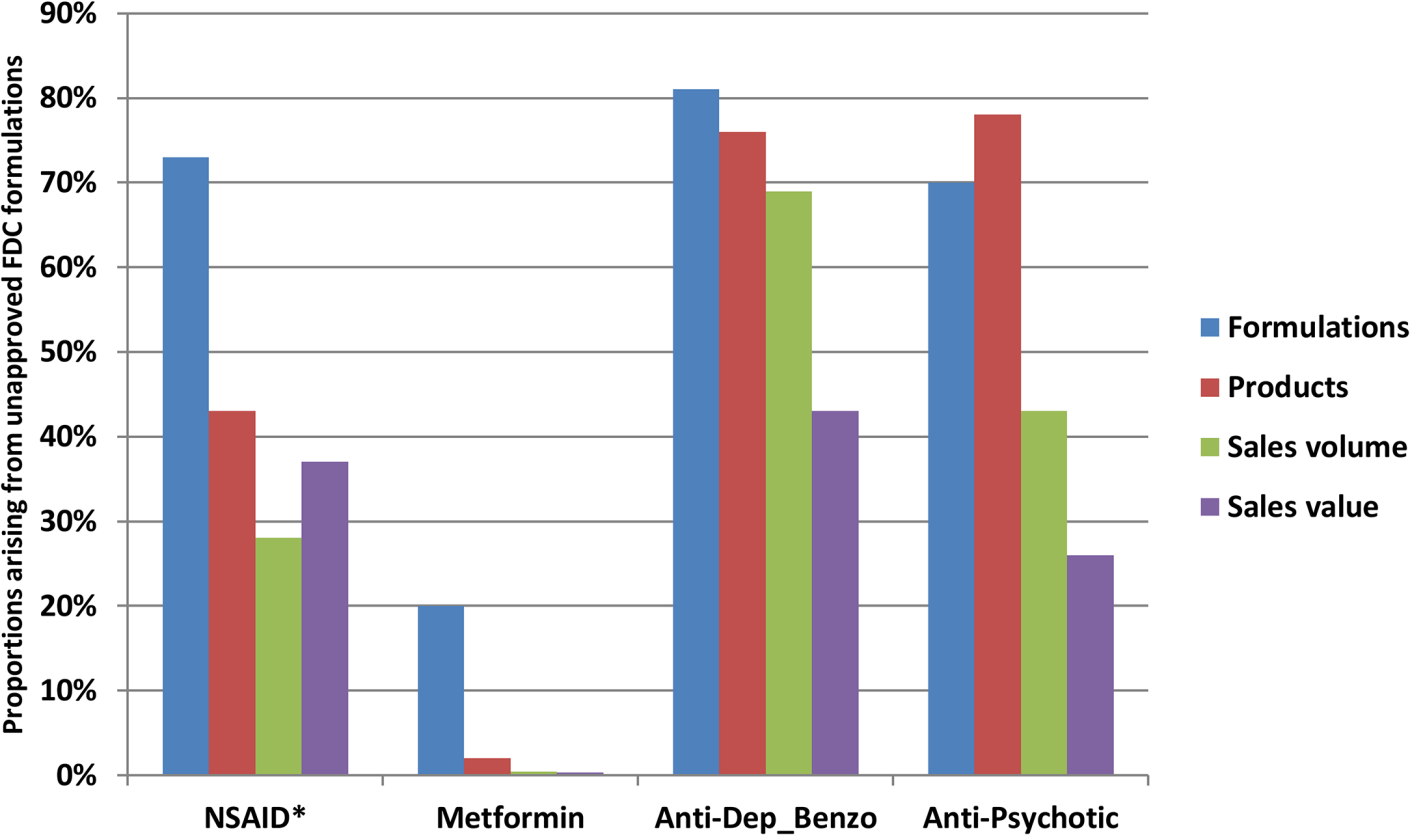
Proportions of formulations on the market, products on the market, sales volume, and sales value in each therapeutic area arising from formulations unapproved by the CDSCO. *Excludes “other” formulations.

Individual formulations gave rise to multiple branded products. On average, the ratio of formulations to products was around 1:20 (Table 1), but several formulations had many more products (Tables 2–4 and S2). The 124 NSAID FDC formulations gave rise to 2,739 products (excluding “other”), 25 metformin formulations to 536 products, 16 anti-depressant/benzodiazepine formulations to 301 products, and ten anti-psychotic formulations to 211 products. Overall, unapproved formulations gave rise to substantial proportions of branded products for all groups except metformin FDCs: 43% of NSAID FDC products, 76% of anti-depressant/benzodiazepine FDC products, 78% of anti-psychotic FDC products, and 2% of metformin FDC products (Table 1 and Fig 1)

**Table 2:** Metformin FDC formulations marketed in India.

Formulations of FDCs with Metformin	FDC Sales Volume Nov 2011–Oct 2012 in Millions of Tablets/Capsules	Percent of FDC Total Volume	FDC Value Nov 2011–Oct 2012 in Millions of Rupees (Millions of US Dollars)	Percent of FDC Total Value	Number of Branded Products (with Sales in 2011–2012)	Market Launch Date for First FDC Product	Date of CDSCO First Approval of Original Formulation	Approved in UK/US
Individual formulations								
Glimepiride	1,588.3	34.87%	7,436.92	36.97%	137 (110)	Sep 2002	Nov 2002	N/N
Glimepiride, pioglitazone	772.8	16.97%	4,112.06	20.44%	68 (63)	Dec 2003	Dec 2005	N/N
Glipizide	683.8	15.01%	563.98	2.80%	25 (15)	Nov 1998	Mar 1998	N/Y
Glibenclamide*	487.5	10.70%	977.86	4.86%	33 (24)	Aug 2001	Nov 1995	N/N
Gliclazide	438.1	9.62%	2,007.70	9.98%	102 (69)	Nov 1999	Apr 2005	N/N
Pioglitazone	154.4	3.39%	713.77	3.55%	61 (49)	Feb 2001	Feb 2002	Y/Y
Glibenclamide, pioglitazone	112.1	2.46%	480.80	2.39%	14 (14)	Mar 2007	Feb 2009	N/N
Voglibose	104.0	2.28%	586.45	2.92%	37 (37)	Apr 2007	Jul 2007	N/N
Vildagliptin	99.5	2.18%	1,512.11	7.52%	3 (3)	May 2009	Jul 2008	Y/N
Sitagliptin	56.9	1.25%	1,336.70	6.65%	2 (2)	Apr 2008	Apr 2008	Y/Y
Gliclazide, pioglitazone	21.6	0.47%	118.61	0.59%	4 (4)	Nov 2007	May 2010	N/N
Voglibose, glimepiride	20.3	0.45%	167.28	0.83%	6 (6)	Jul 2011	Not listed	N/N
Acarbose	8.6	0.19%	53.56	0.27%	2 (2)	Apr 2008	Feb 2009	N/N
Repaglinide	2.2	0.05%	17.38	0.09%	1 (1)	Sep 2010	Aug 2010	N/Y
Miglitol	2.1	0.05%	17.26	0.09%	2 (2)	Oct 2007	Oct 2007	N/N
Gliclazide, rosiglitazone	1.9	0.00%	0.001	0.00%	2 (1)	Apr 2007	Aug 2006	N/N
Glimepiride, atorvastatin	1.8	0.04%	11.0	0.05%	8 (4)	Apr 2007	Dec 2009	N/N
Rosiglitazone**	1.5	0.00%	0.68	0.00%	11 (8)	Jan 2001	Oct 2001	N/Y
Glibenclamide, rosiglitazone	0.2	0.00%	0.79	0.00%	5 (4)	May 2004	Aug 2006	N/N
Pioglitazone, picolinic acid	0.1	0.00%	0.34	0.00%	2 (1)	Jun 2003	Not listed	N/N
Glimepiride, rosiglitazone	0.076	0.00%	0.31	0.00%	6 (3)	Jul 2006	Nov 2002	N/N
Nateglinide	0.057	0.00%	0.24	0.00%	1 (1)	Jun 2006	Aug 2004	N/N
Glimepiride, ramipril	0.002	0.00%	0.005	0.00%	2 (1)	Apr 2007	Not listed	N/N
Glimepiride, atorvastatin, ramipril~	0	0.00%	0	0.00%	1 (0)	Apr 2007	Not listed	N/N
**Total**	4,557.8	100.00%	20,115.9 (US$323.0)	100.00%	536 (424, 79%)			
**Marketed before 1 May 2002**	1,765.3	38.7% of total	4,264.0 (US$66.8)	21.2% of total	232 (43% of total)			
CDSCO-approved	1,765.3		4,264.0					
**Marketed after 1 May 2002**	2,792.6	61.3%	15,851.9 (US$25.6)	78.8%	304 (57%)			
CDSCO-approved	2,772.1		15,684.2					
Unapproved	20.4		167.6					
**CDSCO ever-approved formulations**	4,537.4	99.6%	19,948.2 (US$321.9)	99.2%				

Sales volume (millions of tablets/capsules) and value (millions of Rupees [US dollars]) in amounts and as proportions of total volume and value; numbers of branded products of each formulation on the market by October 2012 (numbers of products with sales recorded in 2011–2012); market launch date for the first FDC product; CDSCO first approval date for the original formulation; approval of the FDC in UK and/or US (N = not approved; Y = approved). FDC formulations on the market with and without a record of CDSCO approval are termed respectively “approved” and “unapproved”. Data sources: PharmaTrac; CDSCO-published list of FDC approvals 1961–February 2013. Conversion rate: INR 1 INR = US$0.016. Small differences in totals arise from rounding.

*Glibenclamide is known as glyburide in the US.

**Rosiglitazone approval withdrawn in India 2005.

~Sales recorded in preceding years.

**Table 3:** Anti-depressant/benzodiazepine FDC formulations marketed in India.

Formulations of Anti-Depressant/Benzodiazepine FDCs	FDC Sales Volume Nov 2011–Oct 2012 in Millions of Tablets/Capsules	Percent of FDC Total Volume	FDC Value Nov 2011–Oct 2012 in Millions of Rupees	Percent of FDC Total Value	Number of Branded Products (with Sales in 2011–2012)	Market Launch Date for First FDC Product	Date of CDSCO Approval of Original Formulation
Individual formulations							
Amitriptyline + chlordiazepoxide	229.7	35.97%	399.58	19.87%	62 (45)	May 2001	Not listed
Escitalopram + clonazepam	168.0	26.31%	1,005.95	50.02%	48 (43)	Mar 2005	Aug 2004
Imipramine + diazepam	67.7	10.60%	71.27	3.54%	36 (24)	Nov 2001	Not listed
Alprazolam + propranolol	54.2	8.49%	124.78	6.20%	44 (34)	Jul 2001	Not listed
Alprazolam + melatonin	28.8	4.51%	49.88	2.48%	6 (3)	Dec 2000	Not listed
Alprazolam + sertraline	24.6	3.85%	87.35	4.34%	23 (11)	Apr 2002	Sep 2006
Etizolam + escitalopram	18.5	2.90%	98.29	4.89%	6 (6)	Oct 2008	Not listed
Diazepam + propranolol	11.3	1.78%	23.44	1.17%	16 (12)	Apr 2007	Not listed
Alprazolam + fluoxetine	9.9	1.54%	30.17	1.50%	20 (9)	Nov 2002	Not listed
Paroxetine + clonazepam	5.9	0.93%	52.0	2.59%	1 (1)	Aug 2011	Aug 2010
Duloxetine + mecobalamin	5.1	0.80%	29.94	1.49%	15 (12)	Jul 2006	Not listed
Clonazepam + propranolol	2.8	0.44%	7.88	0.39%	7 (7)	Mar 2009	Not listed
Desvenlafaxine + clonazepam	1.8	0.28%	17.13	0.85%	1 (1)	Nov 2011	Not listed
Imipramine + chlordiazepoxide	0.4	0.06%	0.58	0.03%	3 (2)	Apr 2007	Not listed
Alprazolam + imipramine	0.4	0.06%	0.42	0.02%	5 (1)	Apr 2007	Not listed
Alprazolam + paracetamol*	0	0.00%	0	0.00%	2 (0)	Mar 2003	Not listed
Other combinations	9.5	1.37%	12.53	0.49%	6 (2)	Excluded	Excluded
**Total**	638.5	99.89%	2,011.18 (US$32.54 million)	99.87%	301 (213, 71%)		
**Marketed before 1 May 2002 (excluding “other combinations”)**	405.0	63.4% of total	732.9	36.4% of total			
CDSCO-approved	24.6	3.9%	87.4	4.3%			
Unapproved	380.4	59.6%	645.5	32.0%			
**Marketed after 1 May 2002 (excluding “other combinations”)**	224.1	35.1%	1,265.8	63.0%			
CDSCO-approved	173.9	27.2%	1,058.0	52.6%			
Unapproved	50.2	7.9%	207.9	10.3%			
**CDSCO ever-approved formulations**	198.5	31.1%	1,145.3	56.9%			
**Unapproved**	430.6	67.4%	853.4	42.4%			
**Other combinations**	9.5	1.4%	12.5	0.5%			
**Total**	638.6	100%	2,011.2	99.8%			

Sales volume (millions of tablets/capsules) and value (millions of rupees [US dollars]) in amounts and as proportions of total volume and value in each therapeutic area (2011–2012); numbers of branded products of each FDC formulation on the market by October 2012 (numbers of products with sales recorded in 2011–2012); market launch date for the first FDC product; CDSCO first approval date for the original formulation. Note: To avoid double-counting, FDCs containing an anti-psychotic and an anti-depressant were counted only in the anti-psychotic FDC category. FDC formulations on the market with and without a record of CDSCO approval are termed respectively “approved” and “unapproved”. Data sources: PharmaTrac; CDSCO-published list of FDC approvals 1961–February 2013. Conversion rate: INR 1 = US$0.016. Small differences in totals arise from rounding.

* Sales recorded in 2007 through 2010.

**Table 4:** Anti-psychotic FDC formulations marketed in India.

Formulations of Anti-Psychotic FDCs	FDC Sales Volume Nov 2011–Oct 2012 in Millions of Tablets/Capsules	Percent of FDC Total Volume	FDC Value Nov 2011–Oct 2012 in Millions of Rupees	Percent of FDC Total Value	Number of Branded Products (with Sales in 2011–2012)	Market Launch Date for First FDC Product	Date of CDSCO Approval of Original Formulation
Individual formulations							
Flupenthixol + melitracen	221.9	52.92%	875.30	68.55%	25 (24)	Jan 2005	Oct 1998
Risperidone + trihexyphenidyl	72.8	17.36%	236.57	18.53%	50 (42)	Apr 2001	Not listed
Trifluoperazine + trihexyphenidyl	71.1	16.96%	58.60	4.59%	1 (1)	Aug 2002	Not listed
Chlorpromazine + trihexyphenidyl + trifluoperazine	18.8	4.49%	9.46	0.74%	27 (18)	Sep 2004	Not listed
Olanzapine + fluoxetine	15.2	3.62%	73.34	5.74%	14 (7)	Jul 2003	Feb 2003
Trifluoperazine + chlordiazepoxide	14.0	3.35%	18.46	1.45%	52 (41)	Oct 2001	Not listed
Haloperidol + trihexyphenidyl	2.7	0.64%	2.00	0.16%	13 (9)	May 2004	Not listed
Chlorpromazine + trihexyphenidyl	2.5	0.60%	2.70	0.21%	20 (9)	Jul 2002	Not listed
Chlordiazepoxide + trifluoperazine + trihexyphenidyl	0.2	0.06%	0.22	0.02%	8 (3)	Dec 2002	May 1979
Trihexyphenidyl + thioridazine	0.04	0.01%	0.16	0.01%	1 (1)	Apr 2007	Not listed
**Total**	419.3	100%	1,276.8 (US$20.7 million)	100%	211 (154, 73%)		
**Marketed before 1 May 2002**	86.8	20.7% of total	255.0	20.0% of total			
CDSCO-approved	0	0.0%	0.0	0.0%			
Unapproved	86.8	20.7%	255.0	20.0%			
**Marketed after 1 May 2002**	332.5	79.3%	1,021.8	80.0%			
CDSCO-approved	237.3	56.6%	948.9	74.3%			
Unapproved	95.1	22.7%	72.9	5.7%			
**CDSCO ever-approved formulations**	237.3	56.6%	948.8	74.3%			
**Unapproved**	181.9	43.4%	328.0	25.7%			
**Total**	419.3	100%	1,276.8	100%			

Sales volume (millions of tablets/capsules) and value (millions of rupees [US dollars]) in amounts and as proportions of total volume and value in each therapeutic area (2011–2012); numbers of branded products of each FDC formulation on the market by October 2012 (numbers of products with sales recorded in 2011–2012); market launch date for the first FDC product; CDSCO first approval date for the original formulation. Note: To avoid double-counting, FDCs containing an anti-psychotic and an anti-depressant were counted only in the anti-psychotic FDC category. FDC formulations on the market with and without a record of CDSCO approval are termed respectively “approved” and “unapproved”. Data sources: PharmaTrac; CDSCO-published list of FDC approvals 1961–February 2013. Conversion rate: INR 1 = US$0.016. Small differences in totals arise from rounding.

The 19 NSAID formulations launched on the market before 1 May 2002 gave rise to 1,895 NSAID branded products, of which 31% (*n* = 583) were from unapproved formulations. Of 844 products arising from the 105 formulations marketed after 1 May 2002, 69% (*n* = 582) were from unapproved formulations (Table 1). Among metformin FDCs, no products marketed before 1 May 2002 were from unapproved formulations, compared with 9% (*n* = 28) after 1 May 2002. For antidepressant/benzodiazepine FDCs and anti-psychotic FDCs, 87% and 100%, respectively, of products marketed before 1 May 2002 were from unapproved formulations, compared with 62% and 57% after 1 May 2002 (Table 1).

### FDC Sales: Sales Trends for 2007–2012 and Sales Volumes (2011–2012) Arising from Unapproved Formulations

**Fig 2:**
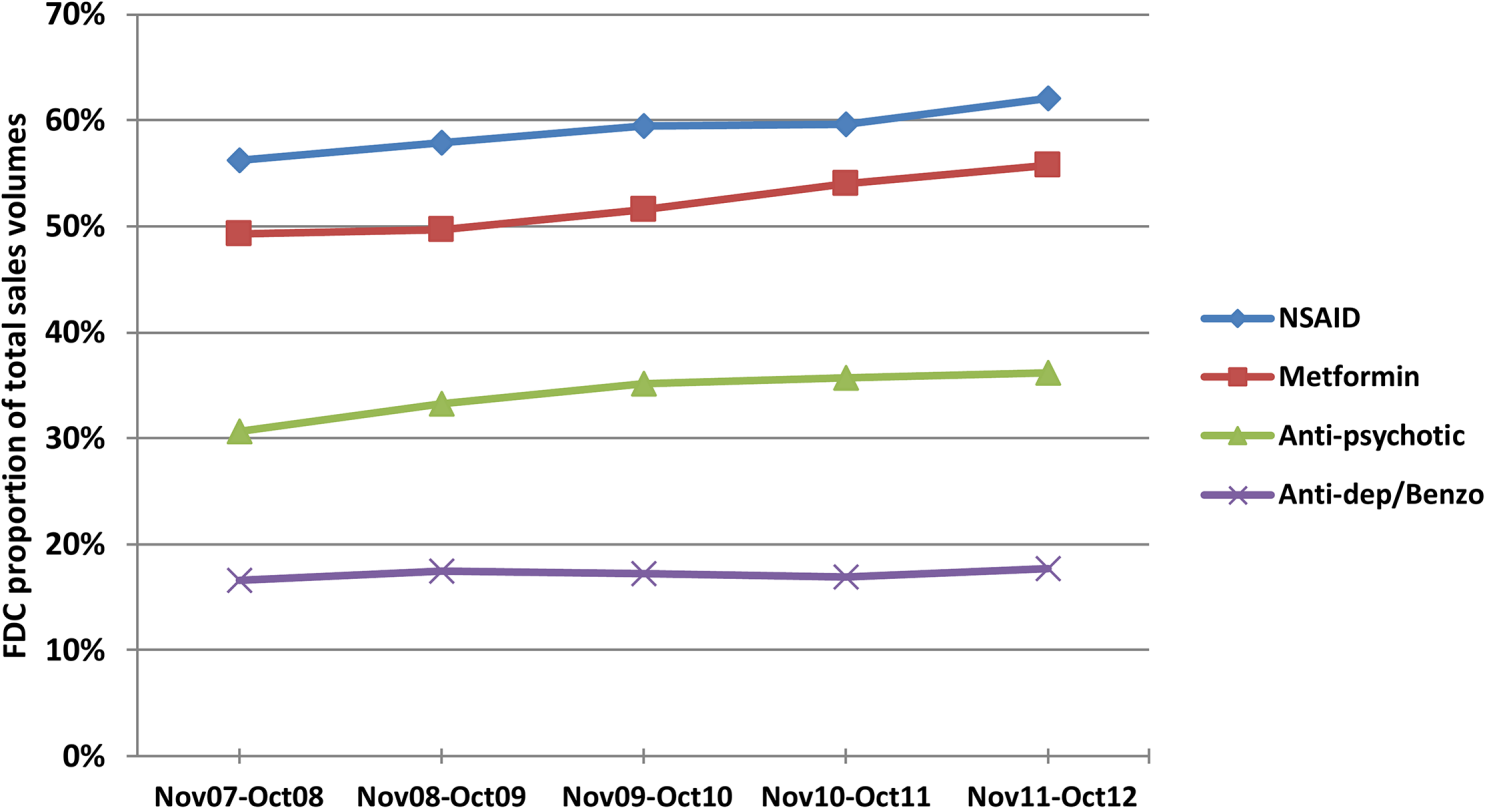
FDCs as proportions of total annual oral drug sales volumes in each therapeutic area, November 2007–October 2012. Metformin FDCs are expressed as a percent of total oral diabetes drugs. Numerator and denominator data are included in S3 Table.

The total oral drug market in each therapeutic area was composed of FDCs and SDFs (Table 1). Sales volumes of NSAIDs and metformin/anti-diabetes drugs were substantial compared with anti-depressants/benzodiazepines and anti-psychotics. During the 5 y examined, FDCs comprised increasing annual proportions of total sales volumes (Fig 2), and by 2011–2012 they accounted for over half of all NSAIDs and oral anti-diabetic drugs sold, one-third of anti-psychotic drugs, and almost one-fifth of anti-depressants/benzodiazepines (Table 1).

Overall, across the four therapeutic areas, approved and unapproved formulations contributed varying proportions of FDC sales volumes: almost all metformin FDC sales were from CDSCO-approved formulations, but formulations unapproved by the CDSCO accounted for over two-thirds of anti-depressant/benzodiazepine FDC sales (69%), almost half of anti-psychotic FDCs (43%), and more than a quarter of NSAID FDCs (28%) (Table 1). Sales values followed generally similar patterns. Fig 1 summarises the proportions of formulations and products on the market and sales volumes and value in 2011–2012 arising in each therapeutic area from unapproved formulations.

Formulations marketed before 1 May 2002 accounted for most of the NSAID FDC sales volume (5,397.3 million tablets/capsules); 14% of this volume arose from unapproved formulations. In contrast, of the sales volume arising from formulations marketed after 1 May 2002 (1,743.8 million tablets/capsules), 74% was from unapproved formulations. Five metformin FDC formulations marketed after 2002 had no record of CDSCO approval, but their contribution to overall sales volume was low (0.4%). For anti-depressant/benzodiazepine and anti-psychotic FDCs, sales arising from formulations marketed before 1 May 2002 were comprised almost entirely of products arising from unapproved formulations; of sales arising from formulations marketed after 1 May 2002, however, 25%–29% were from unapproved formulations.

### Formulation Composition, CDSCO Approval, Sales Volumes, and Numbers of Branded Products Marketed

#### NSAIDs

Four CDSCO-approved formulations, each comprising a NSAID and paracetamol, accounted for 61% of total FDC sales volumes: ibuprofen (2,400 million tablets/capsules), diclofenac (797 million), aceclofenac (715 million), and nimesulide (558 million). Together, the four formulations gave rise to hundreds of branded products, of which some were new to the market during 2007–2012 and had rising sales and some were older and had declining sales (ibuprofen + paracetamol, 158 products; diclofenac + paracetamol, 298; aceclofenac + paracetamol, 327; nimesulide + paracetamol, 292) (S2 Table).

The remaining 30 CDSCO-approved formulations comprised 8% of sales volumes. Most frequently, these were NSAID combinations with muscle relaxants (chlorzoxazone, dicyclomine, drotaverine, thiocolchicoside, tizanidine, tolperisone) or proteolytic enzymes (serratiopeptidase, trypsin-chymotrypsin). There were also combinations with misoprostol, tranexamic acid, and sumatriptan (S2 Table). The marketing date preceded the CDSCO approval date for 23/34 approved formulations.

Ninety unapproved formulations accounted for 28% of sales volumes, and unspecified “other” formulations for 2.6%. The top-selling unapproved formulations were mefenamic acid + dicyclomine, 4.7% of sales; aceclofenac + paracetamol + serratiopeptidase, 4.4%; ibuprofen + paracetamol + caffeine, 2%; and diclofenac + paracetamol + chlorzoxazone, 2%. Other unapproved formulations included FDCs of NSAIDs with paracetamol plus a muscle relaxant, tramadol, dextropropoxyphene, anti-ulcer agent, anti-histamine, caffeine, another NSAID, or sumatriptan (S2 Table).

#### Metformin

CDSCO-approved formulations accounted for almost all sales by volume in 2011–2012 (total sales 4,558 million tablets/capsules) (Table 2). The marketing date preceded the CDSCO approval date for 11/20 approved formulations. The most popular FDCs were dual combinations with sulfonylureas (glimepiride, glipizide, gliclazide, glibenclamide) and a triple combination with glimepiride and pioglitazone (Table 2). These formulations gave rise to 536 branded products, most (*n* = 424, 79%) with sales recorded in 2011–2012. Metformin + glimepiride had 137 different products; metformin + gliclazide, 102; metformin + glimepiride + pioglitazone, 68; and metformin + pioglitazone, 61.

#### Anti-depressants/benzodiazepines

Three CDSCO-approved formulations combining a selective serotonin reuptake inhibitor (SSRI) with a benzodiazepine comprised 31.1% of FDC sales volumes (total sales 638.5 million tablets/capsules) in 2011–2012 (escitalopram + clonazepam, 26.3%; sertraline + alprazolam, 3.9%; paroxetine + clonazepam, 0.9%) (Table 3). The marketing date preceded the CDSCO approval date for 1/3 approved formulations (sertraline + alprazolam). Of 13 formulations unapproved by the CDSCO, seven were FDCs of two centrally acting drugs and three were benzodiazepine + propranolol FDCs (Table 3). Benzodiazepine with tricyclic antidepressant combinations accounted for 47.3% of sales. Among the most popular FDCs, dozens of branded products were available, most with sales recorded in 2011–2012 (Table 3).

#### Anti-psychotics

Of ten formulations on the market, three accounted for 56.6% (237.3 million tablets/capsules) of anti-psychotic sales in 2011–2012 (total sales 419.3 million tablets/capsules) (olanzapine + fluoxetine; chlordiazepoxide + trifluoperazine + trihexyphenidyl; flupenthixol + melitracen) (Table 4). CDSCO approval preceded the marketing date in all cases. The most popular was flupenthixol + melitracen (221.9 million tablets/capsules, 53% of sales volume). Seven formulations included trihexyphenidyl (benzhexol).

### FDC Formulations with State Licenses but Not Approved by the CDSCO

The list of 294 FDCs with state licenses but unapproved by the CDSCO included 72 NSAID FDC formulations, of which 33 were on the market; 15 anti-depressant/benzodiazepine FDC formulations, with five on the market; and one metformin formulation not marketed [14]. No anti-psychotic formulations were listed.

### FDCs Including Drugs Banned, Restricted, or Never Approved Internationally

Multiple formulations, most without CDSCO approval, included drugs banned, restricted, or never approved internationally owing to adverse effects (Table 5) [23–29]. Sales volumes were generally high. None of these drugs had MHRA (UK) or FDA (US) approval.

**Table 5:** Drugs withdrawn or restricted internationally that were included in FDC formulations marketed in India.

Drug Name (FDC Therapeutic Area That Included the Drug)	Withdrawn/Restricted Status Internationally	Reason	Number of FDC Formulations Available in India	Number of Formulations Approved by the CDSCO	FDC Percent Sales in 2011–2012 in India Including the Drug (Millions of Tablets/Capsules)
Nimesulide (NSAIDs)	Banned in multiple countries; EMA restriction 2007, 2011 [23]	Liver toxicity	15	1	NSAIDs 10.1% (723)
Thiocolchicoside (NSAIDs)	EMA restriction 2013 [24]	Aneuploidy	6	5	NSAIDs 2.2% (157)
Dextropropoxyphene (NSAIDs)	Banned in multiple countries; withdrawn in EU [25], also Australia, US	Fatality in overdose	4	0	NSAIDs 0.3% (21)
Metamizole (Analgin) (NSAIDs)	Banned in multiple countries [26]	Agranulocytosis	1 (with nimesulide)	Withdrawn 2013	NSAIDs 0.004% (0.27)
Melitracen (anti-psychotics)	Approved for export only in Denmark [27,28]; manufacturer Lundbeck	Multiple CNS effects	1 (with flupenthixol)	Withdrawn 2013, re-listed, withdrawn 2014	Anti-psychotics 52.9% (222)
Thioridazine (anti-psychotics)	Withdrawn worldwide 2005 [29]	Cardiac arrhythmias; sudden death	1	0	Anti-psychotics 0.008% (0.04)

Drugs withdrawn or restricted internationally but included in FDCs in India, reasons for withdrawal, numbers of FDC formulations available in India, CDSCO approval status, and the proportions (volume) of FDCs sales in India (2011–2012) that included the drugs.

EMA, European Medicines Agency; EU, European Union.

Many NSAID FDC formulations available in India (both approved and unapproved) included muscle relaxants or proteolytic enzymes, of which several were approved as SDFs in the UK and/or US but none were components of NSAID FDCs in either country (Table 6). FDCs including two NSAIDs (diclofenac + nimesulide, mefenamic acid + ibuprofen) were not approved in the UK or the US.

**Table 6:** Muscle relaxants and proteolytic enzymes included in NSAID FDC formulations marketed in India.

NSAID FDC Component	MHRA Market Authorisation as SDF, UK [17]	FDA Approval as SDF, US [18]	Clinical Action	Percent NSAID FDC Sales in 2011–2012 in India Including the Drug (Millions of Tablets/Capsules)
Chlorzoxazone	N	Y	Muscle relaxant	4.09% (300)
Dicyclomine (Dicycloverine)	Y	Y	Muscle relaxant	5.68% (4,165)
Drotaverine	N	N	Muscle relaxant	1.25% (91.9)
Methocarbamol	Y	Y	Muscle relaxant	0.07% (4.8)
Serratiopeptidase	N	N	Proteolytic enzyme	9.73% (713.5)
Tizanidine	Y	Y	Muscle relaxant	1.14% (83.7)
Tolperisone	N	N	Muscle relaxant	0.02% (1.6)
Trypsin-chymotrypsin	N	N	Proteolytic enzyme	0.56% (41.2)

Muscle relaxants and proteolytic enzymes included in NSAID FDC formulations, their MHRA and FDA approval status SDFs, and the proportions of NSAID FDCs sales volumes in India (2011–2012) that included the drugs. N, no; Y, yes.

## Discussion

In quantifying the use of FDCs in four different therapeutic areas and determining the relative contributions of CDSCO-approved and-unapproved FDCs, this study confirms the concerns of the 59th Report about FDC regulation in India. In three of the four areas examined, there were more unapproved formulations on the market than approved formulations. In turn these had given rise to hundreds—and in the case of NSAIDs, thousands—of branded products. Many millions of doses were sold of FDCs that included drugs restricted, banned, or never approved in other countries owing to their association with serious adverse events including fatality.

Multiple formulations of the FDCs studied were marketed without any record of CDSCO approval, supporting the concern that “many FDCs in the market have not been tested for efficacy and safety” (Section 9.2 of [6]). This occurred despite the legal need since 1961 for a new drug to have been approved before manufacture, including an FDC meeting the applicable legal test (see Box 1). Unexplained “ambiguity” before 1 May 2002 regarding the licensing powers of state drug authorities is noted in the 59th Report as a possible contributor (Section 9.2 of [6]). In our analysis of the data in four therapeutic areas, we found no notable differences in the overall proportion of centrally approved formulations after 1 May 2002 compared with before. In our detailed analysis of the national rules (S1 Text), we found no ambiguity relating to the question of whether an FDC needed central approval prior to manufacture. Clearly, however, the legal test until 1988 needed to be interpreted in order to determine its application to FDCs, and practice appears to have varied, as presumably approval was sought for some FDCs but not for others. Numerous FDCs were marketed before the CDSCO approval date, suggesting that in some cases state licenses permitting manufacturing and sale were obtained first and approval sought retrospectively. However, state drug authority records to confirm this were unavailable.

NSAIDs are among the most widely used drugs internationally. Almost 12 billion NSAID tablets/capsules were sold in India in 2011–2012; NSAID FDCs comprised almost two-thirds of these, of which 28% were unapproved formulations. Over 12% of NSAID FDC sales volumes included drugs that have been withdrawn from international markets or have had their use restricted, for example, dextropropoxyphene, metamizole, melitracen, nimesulide, and thiocolchicoside, owing to risks including death in overdose, liver failure, and genetic effects [23–29]. FDCs including two NSAIDs are highly undesirable due to associated gastrointestinal risks [12]. In contrast to NSAIDs, most metformin FDC formulations had CDSCO approval and accounted for practically all FDCs used and over half of total oral anti-diabetic drug sales volumes. Among both groups of CNS drugs examined, unapproved formulations comprised substantial FDC sales volumes: 69% of antidepressant/benzodiazepine FDCs and 43% of anti-psychotic FDCs. Sales volumes of these FDCs were lower than those of NSAID and metformin FDCs, but the combinations were commonly hazardous. Indeed, some anti-psychotic FDCs were potentially lethal. For example, the combination chlorpromazine + trifluoperazine + trihexyphenidyl contains two phenothiazine anti-psychotics independently associated with major CNS toxicity and with cardiotoxicity including sudden death, risks also associated with FDCs containing thioridazine [10,11,29]. Tricyclic antidepressants, benzodiazepines, and antipsychotic medicines are independently associated with increased risk of falls, especially among older patients [30]. Nevertheless, FDCs comprising these sedating classes in single formulations were sold in considerable volumes.

Since 2001, the CDSCO is obliged to be satisfied as to the safety and effectiveness of products approved. The grounds for approval are not published, but the regulator acknowledges having approved some new drugs without clinical data (Section 7 of [6]) [31,32]. Many formulations being sold without approval contravene FDC development guidance on basic pharmacological parameters [8,33]. Pharmacovigilance in India is at an early stage and adverse event reporting rates are very low, so the absence of adverse event reports offers no reassurance of the safety of such formulations (Sections 8 and 12 of [6]) [34–36]. Overall, among the four therapeutic areas examined, most of the FDC formulations available in India were unavailable in either the UK or the US.

The study has limitations. We relied on publicly available records for central approvals and commercial sales records to determine the formulations marketed, the market launch dates, and the sales volumes. These sources were not compiled in order to support research, and they cannot be independently verified. Nevertheless, they are nationwide datasets that permit informative examination of data relevant to complaints about regulation and use in India of FDCs. The sales data reflect mainly private out-of-pocket prescription spending. Sales through hospitals and dispensing doctors may include government-subsidised drugs, but we could not confirm this. In comparing the numbers of formulations on the market in India in each therapeutic area with those in the UK and US, it is possible that some formulations listed in PharmaTrac as “other” were overlooked and not identified by us owing to small volumes sold. We cross-checked FDC formulations approved in the UK and US but not found in PharmaTrac with the MIMS India listings and found two additional metformin FDCs available in India (with linagliptin and saxagliptin) [37]. We found no additional NSAID or psychotropic FDCs listed in MIMS India. Other than the list of 294 banned FDCs, we did not have information on manufacturing/distribution licences granted by state drug authorities. The list of banned FDCs, however, was consistent with the 59th Report’s claim that some FDCs were licensed by states without prior CDSCO approval. Notwithstanding the limitations of the sales data and the absence of systematic central regulatory and state licensing records, this study substantiates the complaints of the 59th Report in demonstrating that regulatory problems in India extend well beyond widely publicised concerns about manufacturing standards.

### Factors Potentially Contributing to the Large Numbers of FDCs

In the four therapeutic areas examined, most of the FDC formulations available in India were unavailable in the UK and US. FDCs were sold in large volumes. They dominated oral NSAID and diabetes drug sales and comprised substantial sales proportions in the CNS areas. In contrast, National Health Service data for publicly subsidised prescribed medicines that were dispensed in England show that metformin FDCs accounted for <5% of oral diabetes drug use in 2012 [38]. There was no FDC dispensing recorded in the other therapeutic areas [38].

Problematic production and use in India of FDCs will not be rectified without addressing the contributing causes. A commonly cited driver, though not discussed in the 59th Report, is manufacturers’ desire to avoid government price controls imposed on drugs included on the National List of Essential Medicines of India [39]. Price control has been evaded by including these drugs in FDC formulations [39,40] (though amendments in the 2013 National Pharmaceutical Pricing Policy may prevent this) [41]. However, price control could also be evaded by manufacturing SDFs of different strength to those on the National List of Essential Medicines of India [39], and the evasion argument does not explain why prescribers prefer FDCs. Neither does it explain the large numbers of FDC products on the market.

Attempts been made to improve India’s drug regulatory situation following the 59th Report. The Drugs Controller General (India) (DCG[I]) requested that states ask manufacturers to prove the safety and efficacy of their FDCs licensed without central approval within a period of 18 mo (January 2013) [42], policy guidelines for approval of FDCs were published (June 2013) [43], and the Chaudhury committee made recommendations on new drugs, clinical trials, and banning of drugs (July 2013) [44]. Legislative underpinning for the 18-mo safety and efficacy review, however, was lacking, and following industry lobbying, the DCG(I) excluded from it FDCs licensed before 21 September 1988 (June 2014) [45]. The president of Indian Drug Manufacturers’ Association subsequently made “an earnest appeal” to the Ministry of Health and Family Welfare to “maintain status quo” for FDCs licensed by states or approved by DCG(I) up to 30 September 2012 [46].

In our view, significant factors contributing to the large number of FDCs on the market include structural problems with provisions of the Drugs and Cosmetics Rules, 1945, stemming partly from the aged Drugs and Cosmetics Act, 1940, and amendments to the rules (2005) [47]. These include: the blurring over time of the initial clarity of responsibility between central government (responsible for imported drugs) and the states (responsible for manufacture, distribution, and sale); the absence of a duty on the regulator in the Drugs and Cosmetics Act, 1940, to be satisfied of a new drug’s safety and effectiveness; the apparently random interchangeability of the different concepts of efficacy and effectiveness; the unclear and contradictory scope of the FDCs that are covered by the 1988 rules, and their inconsistency with WHO guidelines [8]; and the downgrading in 2005 of data submission requirements [40]. The Drugs and Cosmetics (Amendment) Bill introduced in Parliament in 2013 and the proposed version put out for 12 d of consultation by the Ministry of Health and Family Welfare on 31 December 2014 are not aimed at these issues [48,49].

### Recommendations

First, there should be an immediate ban on sales and manufacturing of FDCs not approved by the CDSCO. Withdrawal from the market should be staged, prioritising FDCs that include drugs banned or unapproved internationally and potentially most likely to harm patients. Prescribers need to review the needs of patients taking these FDCs, taper off the drugs, and substitute appropriate alternatives prescribed as SDFs with monitoring of benefit/need. Approved FDC formulations containing drugs that have been withdrawn or restricted by other regulators should be reviewed with the intention of applying similar restrictions in India unless a strong evidence base dictates otherwise. All other approved FDCs should be reviewed for continuing approval. In all cases, the evidence and rationale for decisions need to be made freely available. This means that the Ministry of Health and Family Welfare should exercise its statutory powers to prohibit the import, manufacture, distribution, and sale of FDCs under Section 10A or 26A of the Drugs and Cosmetics Act, 1945 [15], in line with any CDSCO recommendations or, in the event of it not doing so, should explain and publish its reasoning.

Second, transparency and accountability of regulatory decisions must be improved. Complete details of approvals, including the clinical evidence base, need to be made available by the CDSCO for public scrutiny. Support needs to be provided to the CDSCO in terms of planning, information systems, and skilled personnel in order to achieve this.

Third, the CDSCO and the state authorities should establish transparent and public procedures for ensuring that notice of all new drug approvals given by the CDSCO is immediately given to each state licensing authority, and vice versa for all manufacturing, distribution, and sale licences granted by each state. Such notices should be published on the websites of the CDSCO and the state licensing authorities.

Finally, following the DCG(I)’s worrying restriction of the FDC safety and efficacy review, and the 2014 parliamentary elections, the new Parliament should monitor closely the activities of the Ministry of Health and Family Welfare and the CDSCO. If the proposed Drugs and Cosmetics (Amendment) Bill, 2015, reaches Parliament [49], opportunity exists for legislators to propose amendments on safety, effectiveness, rationality, need, and, perhaps most importantly, transparent regulation. Requiring a review of the safety and effectiveness of all currently available FDCs, not unlike the legally required review mandated by the US Congress in 1962, rather than the administrative review that has been shown to be susceptible to industry lobbying, makes eminent sense [50].

## Supporting Information

S1 Table FDC raw data. FDC formulation, brand, manufacturer, and sales volume (in units of ten tablets/capsules) and value (Indian rupees) by month for NSAID, metformin, anti-depressant/benzodiazepine, and anti-psychotic FDCs, November 2007–October 2012.
(XLSX)

S2 Table NSAID FDCs. NSAID FDC formulations marketed in India categorised by formulations marketed before 1 May 2002 and after 1 May 2002. Sales volume (millions of doses [tablets/capsules]) and value (millions of Indian rupees) in amounts and as proportions of total volume and value; numbers of branded products of each formulation on the market by October 2012; numbers of products with sales recorded in 2012; market launch date for the first FDC product; CDSCO first approval date for the formulation; summary of CDSCO-approved and unapproved formulations and sales volumes before/after 1 May 2002 and overall. FDC formulations on the market with and without a record of CDSCO approval are termed respectively “approved” and “unapproved”. Data sources: PharmaTrac; CDSCO-published list of FDC approvals 1961–February 2013.
(XLSX)

S3 Table FDC sales proportions data for Figs 1 and 2. Summary of total annual oral sales volumes (FDCs and SDFs) for NSAID, metformin, anti-depressant/benzodiazepine, and anti-psychotic FDCs, November 2007–October 2012.
(XLSX)

S1 Text Legal analysis of the India Drugs and Cosmetics Rules, 1945. (DOCX)

S2 Text Drugs and Cosmetics Act, 1940. (PDF)

S3 Text Legal rules amendment 14 April 1952. (PDF)

S4 Text Legal rules amendment 3 January 1961. (PDF)

S5 Text Legal rules amendment 13 June 1961. (PDF)

S6 Text Legal rules amendment 21 September 1988. (PDF)

S7 Text Legal rules amendment 17 August 1999. (PDF)

## Abbreviations:

CDSCO: Central Drugs Standard Control Organization (in this paper, the term CDSCO encompasses both the Central Licence Approving Authority and the Drugs Controller General [India]; the Central Licence Approving Authority is defined in the national rules as the Drugs Controller, India)
CNS: central nervous system
DCG(I): Drugs Controller General (India) (the officer who heads up the CDSCO; DCG(I) is the non-statutory term for “Drugs Controller, India”, the statutory term used in the legislation)
FDA: Food and Drug Administration
FDC: fixed dose combination
MHRA: Medicines and Healthcare Products Regulatory Agency
MIMS: Medical Index of Medical Specialties
NSAID: non-steroidal anti-inflammatory drug
SDF: single drug formulation
UK: United Kingdom
US: United States of America
