# Supplementary material for: Use of Fixed Dose Combination (FDC) Drugs in India: Central Regulatory Approval and Sales of FDCs Containing Non-Steroidal Anti-Inflammatory Drugs (NSAIDs), Metformin, or Psychotropic Drugs
Source: PLoS Med. 2015 May 12;12(5):e1001826. doi: 10.1371/journal.pmed.1001826 (PMC4428752; doi:10.1371/journal.pmed.1001826)
Supplement: S1 Text — (DOCX) [file pmed.1001826.s004.docx]

**S1 Text India**

**Detailed analysis of the provisions of the Drugs and Cosmetics Rules 1945 requiring the approval of new drugs before manufacturing**

1. The detailed analysis of the provisions of the Drugs and Cosmetics Rules 1945 (as amended over time) which is set out below is intended to assist in understanding whether, when and how central approval of a fixed dose combination (FDC) drug in India is legally required before a manufacturing licence is granted by States.

2. The analysis begins with the Drugs Act 1940, which placed responsibility for the import of drugs on central government, and the responsibility for manufacture, distribution, and sale on States. It included a definition of a ‘drug’ (see Appendix below), but it did not and still does not include the concepts of a ‘new drug’ or of a ‘fixed dose combination’.

*The category of ‘new drugs’ appears in secondary rules in 1952*

3. In April 1952, Rule 30A was inserted into the 1945 Rules made under the 1940 Act. It introduced the concept of a ‘new drug’, requiring central approval before import. It read as follows:

“(1) No new drug shall be imported except under and in accordance with the permission in writing of the licensing authority.

(2) The importer of a new drug when applying for permission shall produce before the licensing authority all documentary and other evidence relating to its standards of quality, purity and strength and such other information as may be required by the licensing authority including the results of therapeutic trials carried out with it.

Explanation.- For the purpose of this rule, ‘new drug’ means a drug which is not, for the time being, recognised by the Permanent Commission on Biological Standards of the World health organisation or in the latest edition of the British Pharmacopoeia or the British Pharmaceutical Codex or any other Pharmacopoeia recognised in this behalf by the Government of India and includes a patent or proprietary medicine containing such a drug.” (S.R.O. 694 [No. F.1-30/48-D])

4. In January 1961, the definition of a ‘new drug’ in the Explanation to Rule 30A was amended by Rule 2(1) of the Drugs First Amendment Rules 1961, which read as follows:

“for the Explanation to rule 30-A, the following Explanation shall be substituted, namely:-

‘Explanation:-For the purposes of this rule, “new drug” means a drug the composition of which is such that the drug is not generally recognised among experts as safe for use under the conditions recommended or suggested in the label thereof and includes any drug the composition of which is such that the drug, as a result of investigations for determining its safety for use under such conditions, is so recognised, but which has not, otherwise than during the course of such investigations, been used to any large extent or for any appreciable length of time under the said conditions.’ (S.O. 115 [No. F. 14-22/58])

*Central approval before manufacture required since 1961*

5. In June 1961, Rules 69B and 75B were inserted into the rules by the Drugs Third (Amendment) Rules, 1961, requiring pre-manufacture approval of new drugs covering, respectively, non-Schedule C and C(1) drugs, and specified biological and special products that fell within those Schedules. Rule 69B was headed and read as follows:

“Applications to manufacture ‘new drug’ other than the drugs classifiable under Schedule C and C(1) products.-

Subject to the other provisions of these Rules,

(i) No ‘new drug’ shall be manufactured unless it is previously approved by the licensing authority mentioned in rule 21;

(ii) The manufacturer of a ‘new drug’ when applying for approval to the licensing authority mentioned in sub-rule (i) shall produce all documentary and other evidence relating to its standards of quality, purity and strength and such other information as may be required including the results of therapeutic trials carried out with it;

(iii) While applying for a licence to manufacture a ‘new drug’ or its preparations an applicant shall produce along with his application evidence that the drug for the manufacture of which application is made has already been approved.

Explanation.-In this rule ‘new drug’ has the same meaning as in rule 30-A.” [S.O. 1449 (No. F.1-19/59-D)]

The licensing authority mentioned in rule 21 is (and was) “the authority appointed by the Central Government to perform the duties of the licensing authority under these Rules” (Rule 21(b)). This is the Drugs Controller General (India) and the Central Drugs Standard Control Organization (CDSCO) which he heads.

6. The position from June 1961 as regards central approval of an FDC prior to manufacture was therefore that an FDC would have required that approval if it fell within the definition of a ‘new drug’ as set out in Rule 30A. In addition, from that time, companies seeking approval had to produce evidence relating to quality, purity and strength; and evidence of that approval had to be produced by the company when applying subsequently for a manufacturing licence.

*New provisions introduced in 1988*

7. In September 1988, the Drugs and Cosmetics (Eight Amendment) Rules 1988 [G.S.R. 944(E) [No. X-11011/1/87-DMS&PFA] repealed Rules 30A, 69B and 75B, and inserted a new Part XA (Rules 122A-122E) into the Drugs and Cosmetics Rules 1945, headed ‘Import or manufacture of new drug for clinical trials or marketing’. A new Schedule Y was also inserted, headed ‘Requirements and guidelines on clinical trials for import and manufacture of new drug’.

8. As explained in detail below, the summary of the position in paragraph 6 above continued to be the basic position following the 1988 changes, but with two new additional aspects which require consideration: a new definition of ‘new drug’ which expressly referred to certain FDCs, and new data submission requirements specifically for FDCs based on a typology.

9. The new definition of a ‘new drug’ under Rule 122E was as follows:

“For the purpose of this part, new drug shall mean and include:-

(a) A new substance of chemical, biological or biotechnological origin; in bulk or prepared dosage form; used for prevention, diagnosis, or treatment of disease in man or animal; which, except during local clinical, trials, has not been used in the country to any significant extent; and which except during local clinical trials, has not been recognised in the country as effective and safe for the proposed claims.

(b) A drug already approved by the licensing authority mentioned in rule 21 for certain claims, which is now proposed to be marketed with modified or new claims, namely, indications, dosage, form (including sustained release dosage form) and route of administration.

(c) A fixed dose combination of two or more drugs, individually approved earlier for certain claims, which are now proposed to be combined for the first time in a fixed ratio, or if the ratio of ingredients in an already marketed combination is proposed to be changed, with certain claims, viz indications, dosage, dosage form (including sustained release dosage form) and route of administration (See item (b) and (c) of Appendix IV (*sic*) to Schedule-Y).

Explanation:- For the purpose of this rule -

(i) all vaccines shall be new drugs unless certified otherwise by the licensing authority under rule 21:

(ii) a new drug shall continue to be considered as new drug for a period of four years from the date of its first approval or its inclusion in the Indian Pharmacopoeia whichever is earlier.”

10. The requirements for pre-import permission and pre-manufacture approval for new drugs from the central licensing authority was reiterated in Rule 122A, and in Rule 122B (for non-Schedule C and C(1) drugs) and Rule 122C (for drugs within those Schedules), respectively.

11. Rules 122A, 122B and 122C each required the applicants for permission or approval to submit evidence from clinical trials in India (unless the central licensing authority decided in the public interest to grant permission or approval “on the basis of data from other countries”; and the need to submit certain specified types of studies could also be “modified or relaxed” if the new drug had been marketed for several years in other countries and there was adequate published safety evidence).

12. Rules 122B and 122C also required in identical terms (excepting grammar and punctuation) the submission to States of evidence of prior central approval: this extract is from Rule 122B(3):

“When applying for approval to manufacture of a new drug under sub-rule (1) or its preparations, to the State Licensing Authority, an applicant shall produce along with his application, evidence that the drug for the manufacture of which application is made has already been approved by the licensing authority mentioned in rule 21”

13. In addition, separate and new data submission requirements for FDCs were stated in Rule 122D. The entire Rule 122D read as follows:

“Application for permission to import or manufacture fixed dose combination of drugs:

Application for permission to import or manufacture fixed dose combination of drugs already approved as individual drugs by the licensing authority mentioned in rule 21, shall accompany information and data as given in Appendix VI of Schedule Y.”

14. [In parenthesis, it will be noted from paragraph 9 above that there is a reference in Rule 122E(c) to “Appendix IV”. This should have been a reference to “Appendix VI” and it was corrected in 2005 (G.S.R. 32(E)). It also seems, grammatically, that in Rule 122D “shall accompany” should read “shall be accompanied by”.]

15. After September 1988 therefore, FDCs combining drugs for the first time which had been individually approved previously, or previously-combined FDCs with new claims, were expressly included within the definition of a ‘new drug’ under Rule 122E(c). Those FDCs therefore required central approval prior to manufacturing under Rules 122B or 122C, and applicants had to submit evidence to State authorities of that prior approval. This is reflected in the heading of Rule 122D: “Application for permission to import or manufacture fixed dose combination of drugs”.

16. If the drugs combined in the FDC had not been individually approved previously, then the FDC would still require approval if it fell within Rule 122E(a): by definition, Rule 122E(b) could not apply.

17. This is reflected in the first category of FDCs defined in Appendix VI of Schedule 1 (see Box 1 below) which “includes those in which one or more of the active ingredients is a new drug. Such FDC are (*sic*) treated in the same way as any other new drug, both for clinical trials and for marketing permission (see rule 122E, item (a))”. It should be noted, however, that the requirement for prior approval arises from the logic of Rule 122E, in association with Rules 122A, 122B and 122C: the first group in Appendix VI of Schedule 1 is a reflection of that requirement, not its source.

*Box 1: Drugs and Cosmetics Rules 1945, Schedule Y, Appendix VI as inserted in 1988 (emphases added)*

| APPENDIX VI  Fixed dose Combinations (FDC) fall into four groups and their data requirements accordingly.  (a) The first group of FDC includes those in which one or more of the active ingredients is a new drug. Such FDC are treated in the same way as any other new drug, both for clinical trials and **for marketing permission** (see rule 122E, item (a)).  (b) The second group of FDC includes those in which active ingredients already approved/marketed individually are combined for the first time, for a particular claim and where the ingredients are likely to have significant interaction of a pharmacodynamic or pharmacokinetic nature (see rule 122E, item (c)). For permission to carry out clinical trials with such FDC, a summary of available pharmacological, toxicological and clinical data on the individual ingredients should be submitted, along with the rationale for combining them in the proposed ratio. In addition, acute toxicity data (LD 50) and pharmacological data should be submitted on the individual ingredients as well as their combination in the proposed ratio. If clinical trials have been carried out with the FDC in other countries, reports of such trials should be submitted. If the FDC is marketed abroad, the regulatory status in other countries should be stated. (See Appendix I, item 9).  **For marketing permission**, the reports of clinical trials carried out with the FDC in India should be submitted. The nature of trials depending on the claims to be made and the data already available.  (c) The third group of FDC includes those which are already marketed, but in which it is proposed either to change the ratio of active ingredients or to make a new therapeutic claim.  For such FDC, the appropriate rationale should be submitted to obtain a permission for clinical trials, and the reports of trials should be submitted to obtain **a marketing permission.** The nature of trials will depend on the claims to be made and the data already available.  (d) The fourth group of FDC includes those whose individual active ingredients have been widely used in particular indication for years, their concomitent (*sic*) use is often necessary and no claim is proposed to be made other than convenience, and a stable acceptable dosage-form and the ingredients are unlikely to have significant interaction of a pharmacodynamic or pharmacokinetic nature.  No additional animal or human data are generally required for these FDC, and **marketing permission** may be granted if the FDC has an acceptable rationale. |
| --- |

18. Problems with the drafting of Appendix VI have been previously identified (Roderick P, Mahajan R, McGettigan P, Pollock A, Jeffery R. (2014) Need for a New Drugs Bill. Economic and Political Weekly; XLIX No. 33:15-19 *and* Roderick P, Mahajan R, McGettigan P, Pollock AM. (2014) India should introduce a new Drugs Act. The Lancet; 383(9913):203–6.)

19. In the context of the present focus, the main point to note is that Appendix VI sets out different data submission requirements for different groups of FDCs for the purposes of applying for prior central approval for import or manufacture (i.e., “marketing permission”).

*In 1999 the definition of a ‘new drug’ was amended*

20. In August 1999, the Drugs and Cosmetics (Third Amendment) Rules 1999 substituted a new definition for the first category of a ‘new drug’ in Rule 122E(a), as follows:

“(a) A drug, as defined in the Act including bulk drug substance which has not been used in the country to any significant extent under the conditions prescribed, recommended or suggested in the labelling thereof and has not been recognised as effective and safe by the licensing authority mentioned under rule 21 for the proposed claims: Provided that the limited use, if any, has been with the permission of the licensing authority.” (G.S.R. 591(E), [No.X-11014/2/97-DMS & PFA])

21. This definition remains in place, and so since August 1999 an FDC which is not a ‘new drug’ within Rule 122E(c) will still be a ‘new drug’ – and so require prior central approval for import and manufacture - if (i) it has not been used significantly in India, and if there has been any limited use, such use must have been with the permission of the central licensing authority (ii) under the conditions prescribed, recommended or suggested in its label, and (iii) has not been recognised by the central authority as effective and safe for the proposed claims.

*Further amendments were made in 2001*

22. Several amendments were made to Part XA of the rules in 2001 (G.S.R. 900(E)). The issues are considered below under four headings:

*(1) Prohibitions on import and manufacture of new drugs without central approval continued*

Table 1 below compares the wording of these prohibitions in Rules 122A, 122B and 122D, as introduced in 1988, and as introduced in 2001. They are, basically, identical. It will also be noted that the 2001 amendments specified the application forms to be used and the fees payable in different circumstances.

*Table 1: A comparison between the 1988 and 2001 sub-rules*

*on prohibiting import and manufacture of new drugs without central approval*

| 1988 | 2001 |
| --- | --- |
| **122A. Application for permission to import new drug.**  (1) No new drug shall be imported except under and in accordance with the permission in writing of the licensing authority defined in clause (b) of rule 21. | **122A. Application for permission to import new drug.**  (1) (a) No new drug shall be imported, except under, and in accordance with, the permission granted by the Licensing Authority as defined in clause (b) of rule 21.  (b) An application for the grant of permission to import a new drug shall be made in Form 44 to the Licensing Authority, accompanied by a fee of fifty thousand rupees:  Provided that where a subsequent application by the same applicant for that drug, whether in modified dosage form or with new claims, is made, the fee to accompany such application shall be fifteen thousand rupees.  Provided further that any application received after one year of the grant of approval for the import and sale of new drug, shall be accompanied by a fee of fifteen thousand rupees and such information and data as required by Appendix I or Appendix IA of Schedule Y, as the case may be. |
| **122B. Application for approval to manufacture new drug other than the drugs classifiable under Schedule C and C (1).**  (1) No new drug other than the drug classifiable under Schedule C and C (1) shall be manufactured unless it is approved by the licensing authority defined in rule 21. | **122B. Application for approval to manufacture new drug.**  (1)(a) No new drug shall be manufactured for sale unless it is approved by the Licensing Authority as defined in clause (b) of rule 21.  (b) An application for the grant of approval to manufacture the new drug and its formulations shall be made in Form 44 to the Licensing Authority as defined in clause (b) of Rule 21 and shall be accompanied by a fee of fifty thousand rupees:  Provided that where the application is for permission to import a new drug (bulk drug substance) and grant of approval to manufacture its formulation/s, the fee to accompany such application shall be fifty thousand rupees only.  Provided further that where a subsequent application by the same applicant for that drug, whether in modified dosage form or with the new claims, is made, the fee to accompany such subsequent application shall be fifteen thousand rupees:  Provided also that any application received after one year of the grant of approval for the manufacture for sale of the new drug, shall be accompanied by a fee of fifteen thousand rupees and such information and data as required by Appendix 1 or Appendix 1-A of Schedule Y, as the case may be. |
| **Rule 122C**, which had required prior approval for the manufacture of new drugs under Schedule C and C(1) was omitted in 2001, as the distinction was ended, and so since then Rule 122B has applied to new drugs without the distinction | |
| **122D. Application for permission to import or manufacture fixed dose combination of drug:**  Application for permission to import or manufacture fixed dose combinations of drugs already approved as individual drugs by the licensing authority mentioned in rule 21, shall accompany information and data as given in Appendix VI of Schedule Y. | **122D. Permission to import or manufacture fixed dose combination.**  (1) An application for permission to import or manufacture fixed dose combination of two or more drugs as defined in clause (c) of rule 122-E shall be made to the Licensing Authority as defined in clause (b) of Rule 21 in Form 44, accompanied by a fee of fifteen thousand rupees and shall be accompanied by such information and data as is required in Appendix VI of Schedule Y. |

*(2) New duties on the central licensing authority introduced*

New duties were imposed on the central licensing authority to be satisfied of the safety and effectiveness of new drugs, including FDCs; and the forms that the authority was to use in giving permission or approval were specified. The new provisions in Rules 122A(3), 122B(2A) and 122D(2) are set out in Box 2 below. They set out the duty of the authority when deciding whether to give permission or approval under Rule 122A(1), 122B(1) and 122D(1), respectively.

*Box 2: new duties on the central authority to be satisfied about safety and effectiveness*

| **122A. Application for permission to import new drug.**  (3) The Licensing Authority, after being satisfied that the drug if permitted to be imported as raw material (bulk drug substance) or as finished formulation shall be effective and safe for use in the country, may issue an import permission in Form 45 and/or Form 45-A, subject to the conditions stated therein: Provided that the Licensing Authority shall, where the data provided or generated on the drug is inadequate, intimate the applicant in writing, and the conditions, which shall be satisfied before permission could be considered.  **122B. Application for approval to manufacture New Drug other than the Drugs classifiable under Schedule C and C (1).**  (2A) The Licensing authority as defined in clause (b) of rule 21 after being satisfied that the drug if approved to be manufactured as raw material (bulk drug substance) or as finished formulation shall be effective and safe for use in the country, shall issue approval in Form 46 and/or Form 46A, as the case may be, subject to the conditions stated therein: Provided that the Licensing Authority shall, where the data provided or generated on the drug is inadequate, intimate the applicant in writing, and the conditions, which shall be satisfied before permission could be considered.  **122D. Permission to import or manufacture fixed dose combination.**  (2) The Licensing Authority after being satisfied that the fixed dose combination if approved to be imported or manufactured as finished formulation shall be effective and safe for use in the country, shall issue permission in Form 45 or Form 46, as the case may be, subject to the conditions stated therein: Provided that the Licensing Authority shall, where the data provided or generated on the fixed dose combination is inadequate, intimate the applicant in writing, and the conditions which shall be satisfied before grant of approval/permission could be considered. |
| --- |

*(3) Need to submit evidence to States of prior approval continued*

The 2001 amendments did not change the duty of applicants seeking manufacturing licences for new drugs to inform States of prior central approval (see Table 2 below).

*Table 2: A comparison between the 1988 and 2001 sub-rules on the requirement to provide States with evidence of prior central approval when applying for a manufacturing licence*

| 1988 | 2001 |
| --- | --- |
| **122B. Application for approval to manufacture New Drug other than the Drugs classifiable under Schedule C and C (1).**  (3) When applying for approval to manufacture of a new drug under sub-rule (1) or its preparations, to the State Licensing Authority, an applicant shall produce along with his application, evidence that the drug for the manufacture of which application is made has already been approved by the licensing authority mentioned in rule 21 | **122B. Application for approval to manufacture new drug.**  (3) When applying for approval to manufacture a new drug under sub-rule (1) or its preparations, to the State Licensing Authority, an applicant shall produce along with his application, evidence that the drug for the manufacture of which application is made has already been approved by the Licensing Authority mentioned in Rule 21 |

*(4) Power to suspend or cancel permission or approval if failure to comply with conditions*

A new Rule 122DB was inserted in 2001, conferring a power on the central authority in the following terms:

*“If the importer or manufacturer under this Part fails to comply with any of the conditions of the permission or approval, the Licensing Authority may, after giving an opportunity to show cause why such an order should not be passed, by an order in writing stating the reasons therefor, suspend or cancel it.”*

A new Rule 122Dc was also inserted, allowing an appeal within sixty days to the Central Government.

*New sub-rules were inserted in 2002*

23. Rule 69 deals with the application for the grant or renewal of a licence to manufacture for sale or for distribution of drugs, other than those specified in Schedules C and C (1), to the State licensing authority. In 2002, a new Rule 69(6) was inserted:

“Where an application under this rule is for the manufacture of drugs formulations falling under the purview of new drug as defined in rule 122E, such application shall also be accompanied with approval, in writing in favour of the applicant, from the licensing authority as defined in clause (b) of rule 21” (G.S.R. 311(E), dated 1^st^ May 2002)

24. An identical sub-rule was inserted as Rule 75(6), covering such licences for drugs specified in Schedules C and C(1).

25. These rules go further than Rule 122B(3), in that they specify written approval in favour of the applicant having to be submitted. They do not distinguish between categories of new drugs, and do not expressly mention FDCs (which would have been unnecessary). They appear to be reformulations of the provisions in Rules 69B(iii) and 75B(iii) – introduced in June 1961 and omitted by the 1988 amendments and replaced by Rules 122B(3) and 122C(3), respectively – which provided that “[w]hile applying for a licence to manufacture a ‘new drug’ or its preparations an applicant shall produce along with his application evidence that the drug for the manufacture of which application is made has already been approved”.

26. Further amendments have been made to the rules since 2002. They include the downgrading in 2005 of data submission requirements for FDCs (Roderick P, Mahajan R, McGettigan P, Pollock A, Jeffery R. (2014) Need for a New Drugs Bill. Economic and Political Weekly; XLIX No. 33:15-19), but they do not assist in understanding the circumstances in which an FDC needs central approval prior to manufacture.

*Conclusion*

27. The above analysis demonstrates that ‘new drugs’ have required central approval before manufacture since 1961, and that whether, when and how that approval is required for an FDC depends on the particular FDC falling within the different definitions of a ‘new drug’ as they applied from 1961-1988, from 1988-1999, and since 1999. We are led to the following conclusions:

*1961-1988*

(1) Prior central approval for manufacture was required for an FDC after June 1961, until September 1988, if either:

1. the composition of the FDC in question was not at the time generally recognised among experts as safe for use under the conditions recommended or suggested in the label; or
2. if the composition of the FDC, as a result of investigations for determining its safety for use under such conditions, was so recognised, but it had not, otherwise than during the course of such investigations, been used to any large extent or for any appreciable length of time under those conditions,

and those applying to a State for a licence to manufacture an FDC or its preparations which fell within (a) or (b) were required to produce along with their application evidence that the drug had already been centrally approved.

*1988-1999*

(2) Between September 1988 and August 1999, prior central approval for import and manufacture of an FDC was legally required if:

1. the drugs being combined (i) had been individually approved earlier for certain claims, and (ii) it is proposed to combine them for the first time in a fixed ratio; or
2. the drugs being combined (i) had been individually approved earlier for certain claims, and (ii) it is proposed to change the ratio, indications, dosage, dosage form or route of administration; or
3. of the drugs being combined (i) one or more of them had not been previously approved, and (ii) (at least) the combination (and, possibly, each of the individually unapproved drugs) constituted a new substance of chemical, biological or biotechnological origin in bulk or prepared dosage form used for prevention, diagnosis, or treatment of disease in man or animal; which, except during local clinical, trials, has not been used in the country to any significant extent; and which except during local clinical trials, has not been recognised in the country as effective and safe for the proposed claims,

and those applying to a State had to produce evidence that the drug had already been centrally approved. The requirement in effect lapsed after four years from either the date of the first approval, or, if earlier, from the date of the inclusion of the FDC in the Indian Pharmacopoeia.

*Since August 1999*

(3) Since August 1999, the position has been the same as under (2) above, but the criteria in (c)(ii) have changed, so that since August 1999 (c) would read:

of the drugs being combined (i) one or more of them has not been previously approved and (ii) (at least) the combination (and, possibly, each of the individually unapproved drugs) constitutes a drug within the definition of the Act [see Appendix below] including drug bulk substance - if it has not been used significantly in India (and if there has been any limited use, such use must have been with the permission of the central licensing authority) under the conditions prescribed, recommended or suggested in its label, and has not been recognised by the central authority as effective and safe for the proposed claims.

**Appendix**

**The original and current definition of a ‘drug’ in section 3(b) of the Drugs Act 1940**

**Original definition in 1940**

“3. In this Act, unless there is anything repugnant in the subject or context,-

(a)…

(b) ‘drug’ includes all medicines for internal or external use of human beings or animals, and all substances intended to be used for or in the treatment, mitigation or prevention of disease in human beings or animals, other than medicines and substances exclusively used or prepared for use in accordance with the Ayurvedic or Unani systems of medicine;”

**Current definition**

“3. In this Act, unless there is anything repugnant in the subject or context,⎯

(a)…

^[[1]](#footnote-1)^(b) “drug” includes—

(i) all medicines for internal or external use of human beings or animals and all substances intended to be used for or in the diagnosis, treatment, mitigation or prevention of any disease or disorder in human beings or animals, including preparations applied on human body for the purpose of repelling insects like mosquitoes;

(ii) such substances (other than food) intended to affect the structure or any function of the human body or intended to be used for the destruction of vermin or insects which cause disease in human beings or animals, as may be specified from time to time by the Central Government by notification in the Official Gazette;

(iii) all substances intended for use as components of a drug including empty gelatin capsules; and

(iv) such devices intended for internal or external use in the diagnosis, treatment, mitigation or prevention of disease or disorder in human beings or animals, as may be specified from time to time by the Central Government by notification in the Official Gazette, after consultation with the Board;”

**Sources used for this analysis:**

(1) The Drugs Act 1940 (pdf attached)

(2) The Drugs Act 1940, as amended, and the The Drugs and Cosmetics Rules 1945, as amended, and set out in Deshpande S W, Gandi N, Drugs and Cosmetics Act, 1940 and Rules, 1945. Mumbai:Susmit Publishers, 2012, 6th Edition

(3) The Constitution of India

(4) Copies of the following five amendments to The Drugs and Cosmetics Rules, as gazette and obtained from the Judges’ Library, Bombay High Court (pdfs attached)

(i) 14^th^ April 1952, S.R.O. 694 [No. F.1-30/48-D]

(ii) 3^rd^ January 1961, Drugs First Amendment Rules 1961, S.O. 115 [No. F. 14-22/58]

(iii) 13^th^ June 1961, Drugs Third (Amendment) Rules 1961, S.O. 1449 [No. F.1-19/59-D]

(iv) 21^st^ September 1988, Drugs and Cosmetics (Eight Amendment) Rules 1988, G.S.R. 944(E) [No. X-11011/1/87-DMS & PFA]

(v) 17^th^ August 1999, Drugs and Cosmetics (Third Amendment) Rules 1999, G.S.R. 591(E), [No.X-11014/2/97-DMS & PFA]

We also used a similarly-obtained gazette copy of the 2005 amendments to the rules (71 pages), but have not scanned this document to produce a pdf file as it is not central to the detailed analysis (20^th^ January 2005, Drugs and Cosmetics (IInd Amendment) Rules 2005, G.S.R. 32(E) [F.No.X-11014/1/2003-DMS & PFA.]

1. The original section 3(b) was substituted in 1955 by paragraphs (i) and (ii); paragraph (i) was subsequently substituted and the current version above dates from 1982; in 1964, “vermins” in paragraph (ii) was substituted by “vermin”; paragraphs (iii) and (iv) were inserted in 1982. [↑](#footnote-ref-1)
