# Supplementary material for: Use of Fixed Dose Combination (FDC) Drugs in India: Central Regulatory Approval and Sales of FDCs Containing Non-Steroidal Anti-Inflammatory Drugs (NSAIDs), Metformin, or Psychotropic Drugs
Source: PLoS Med. 2015 May 12;12(5):e1001826. doi: 10.1371/journal.pmed.1001826 (PMC4428752; doi:10.1371/journal.pmed.1001826)
Supplement: S2 Text — (PDF) [file pmed.1001826.s005.pdf]

A

COLLECTION

OF

THE ACTS

OF

THE CENTRAL LEGISLATURE AND OF THE GOVERNOR GENERAL

FOR THE YEAR

1940

PUBLISHED BY THE MANAGER OF PUBLICATIONS, DELHI.
PRINTED BY THE MANAGER, GOVERNMENT OF INDIA PRESS, NEW DELHI,
1941.

Price anna 1 or 1½d.

GOVERNMENT OF INDIA

LEGISLATIVE DEPARTMENT

THE DRUGS ACT, 1940

(XXIII OF 1940)

PUBLISHED BY THE MANAGER OF PUBLICATIONS, DELHI.
PRINTED BY THE MANAGER, GOVERNMENT OF INDIA PRESS, NEW DELHI,
1940.

Price annas 2 or 3d.

THE DRUGS ACT, 1940.

CONTENTS

CHAPTER I

INTRODUCTORY

SECTIONS

1. Short title, extent and commencement.
2. Application of other laws not barred.
3. Definitions.
4. Presumption as to poisonous substances.

CHAPTER II

THE DRUGS TECHNICAL ADVISORY BOARD, THE CENTRAL DRUGS LABORATORY AND THE DRUGS CONSULTATIVE COMMITTEE

5. The Drugs Technical Advisory Board.
6. The Central Drugs Laboratory.
7. The Drugs Consultative Committee.

CHAPTER III

IMPORT OF DRUGS

8. Standards of quality.
9. Misbranded drugs.
10. Prohibition of import of certain drugs.
11. Application of law relating to sea customs and powers of Customs officers.
12. Power of Central Government to make rules.
13. Offences.
14. Confiscation.
15. Jurisdiction.

CHAPTER IV.

CHAPTER IV

MANUFACTURE SALE AND DISTRIBUTION OF DRUGS

SECTIONS.

16. Standards of quality.
17. Misbranded drugs.
18. Prohibition of manufacture and sale of certain drugs.
19. Pleas.
20. Government Analysts.
21. Inspectors.
22. Powers of Inspectors.
23. Procedure of Inspectors.
24. Persons bound to disclose place where drugs are manufactured or kept.
25. Reports of Government Analysts.
26. Purchaser of drug enabled to obtain test or analysis.
27. Penalty for manufacture, sale, etc., of drugs in contravention of this Chapter.
28. Penalties for giving false warranty or misuse of warranty.
29. Penalty for use of Government Analyst's report for advertising.
30. Penalty for subsequent offences.
31. Confiscation.
32. Cognisance of offences.
33. Power of Provincial Government to make rules.
34. Protection to persons acting under this Chapter.

THE SCHEDULE.—Standards to be complied with by imported drugs and by drugs manufactured for sale, sold, stocked or exhibited for sale, or distributed.

ACT No. XXIII OF 1940.

[PASSED BY THE INDIAN LEGISLATURE]

(Received the assent of the Governor General on the 10th April, 1940)

An Act to regulate the import, manufacture, distribution and sale of drugs.

WHEREAS it is expedient to regulate the import into, and the manufacture, distribution and sale in, British India of drugs ;

AND WHEREAS the Legislatures of all the Provinces have passed resolutions in terms of section 103 of the Government of India Act, 1935, in relation to such of the above-mentioned matters and matters ancillary thereto as are enumerated in List II of the Seventh Schedule to the said Act ;

It is hereby enacted as follows :—

CHAPTER I.

INTRODUCTORY.

1. (*1*) This Act may be called the Drugs Act, 1940. Short title, extent and commencement.

(*2*) It extends to the whole of British India.

(*3*) It shall come into force at once ; but Chapter III shall take effect only from such date as the Central Government may, by notification in the official Gazette, appoint in this behalf, and Chapter IV shall take effect in a particular Province only from such date as the Provincial Government may, by like notification, appoint in this behalf.

2. The provisions of this Act shall be in addition to, and not in derogation of, the Dangerous Drugs Act, 1930, and any other law for the time being in force. Application of other laws not barred. II of 1930.

3. In this Act, unless there is anything repugnant in the subject or context,— Definitions.

(*a*) "the Board" means the Drugs Technical Advisory Board constituted under section 5 ;

(*b*) "drug"

(*b*) " drug " includes all medicines for internal or external use of human beings or animals, and all substances intended to be used for or in the treatment, mitigation or prevention of disease in human beings or animals, other than medicines and substances exclusively used or prepared for use in accordance with the Ayurvedic or Unani systems of medicine ;

(*c*) " to import ", with its grammatical variations and cognate expressions, means to bring into British India ;

(*d*) " patent or proprietary medicine " means a drug which is a remedy or prescription prepared for internal or external use of human beings or animals, and which is not for the time being recognised by the Permanent Commission on Biological Standardisation of the League of Nations or in the latest edition of the British Pharmacopœia or the British Pharmaceutical Codex or any other pharmacopœia authorised in this behalf by the Central Government after consultation with the Board ;

(*e*) " prescribed " means prescribed by rules made under Chapter II or Chapter III by the Central Government, or under Chapter IV by the Provincial Government.

Presumption as to poisonous substances.

4. Any substance specified as poisonous by rule made under Chapter III or Chapter IV shall be deemed to be a poisonous substance for the purposes of Chapter III or Chapter IV, as the case may be.

CHAPTER II.

THE DRUGS TECHNICAL ADVISORY BOARD, THE CENTRAL DRUGS LABORATORY AND THE DRUGS CONSULTATIVE COMMITTEE.

The Drugs Technical Advisory Board.

5. (*1*) The Central Government shall, as soon as may be, constitute a Board (to be called the Drugs Technical Advisory Board) to advise the Central Government and

and the Provincial Governments on technical matters arising out of the administration of this Act and to carry out the other functions assigned to it by this Act.

(*2*) The Board shall consist of the following members, namely :—

(*i*) the Director-General, Indian Medical Service, *ex officio*, who shall be Chairman ;

(*ii*) the Director of the Central Drugs Laboratory, *ex officio* ;

(*iii*) the Director of the Central Research Institute, *ex officio* ;

(*iv*) the Director of the Imperial Veterinary Research Institute, Muktesar, *ex officio* ;

(*v*) the Chief Chemist, Central Revenues, *ex officio* ;

(*vi*) two persons holding the appointment of Government Analyst under this Act, to be nominated by the Central Government ;

(*vii*) one pharmacologist and one pharmaceutical chemist to be elected by the Scientific Advisory Board of the Indian Research Fund Association ;

(*viii*) three persons to be elected by the Medical Council of India, two of whom shall be from among teachers of medicine or therapeutics on the staff of a university or college in British India providing a course of study which qualifies for admission to the examination for a degree which is a recognised qualification under the Indian Medical Council Act, 1933, and one shall be a registered medical practitioner not being a servant of the Crown ;

(*ix*) one member of the pharmaceutical profession to be nominated by the Central Government ;

(*x*) two persons to be elected by the Council of the Indian Chemical Society ;

(*xi*) one

(*xi*) one person to be elected by the Central Council of the Indian Medical Association and one person to be elected by the branches in India of the British Medical Association.

(*3*) The nominated and elected members of the Board shall hold office for three years, but shall be eligible for re-nomination and re-election.

(*4*) The Board may, subject to the previous approval of the Central Government, make by-laws fixing a quorum and regulating its own procedure and the conduct of all business to be transacted by it.

(*5*) The Board may constitute sub-committees and may appoint to such sub-committees for such periods, not exceeding three years, as it may decide, or temporarily for the consideration of particular matters, persons who are not members of the Board.

(*6*) The functions of the Board may be exercised notwithstanding any vacancy therein.

(*7*) The Central Government shall appoint a person to be Secretary of the Board and shall provide the Board with such clerical and other staff as the Central Government considers necessary.

The Central Drugs Laboratory.

6. (*1*) The Central Government shall, as soon as may be, establish a Central Drugs Laboratory under the control of a Director to be appointed by the Central Government, to carry out the functions entrusted to it by this Act or any rules made under this Chapter :

Provided that, if the Central Government so prescribes, the functions of the Central Drugs Laboratory in respect of any drug or class of drugs shall be carried out at the Central Research Institute, Kasauli, or at any other prescribed Laboratory and the functions of the Director of the Central Drugs Laboratory in respect of such drug or class of drugs shall be exercised by the Director of that institute or of that other Laboratory, as the case may be.

(*2*) The Central Government may, after consultation with the Board, make rules prescribing—

(*a*) the functions of the Central Drugs Laboratory ;

(*b*) the procedure for the grant of certificates of registration under this Act by the said Laboratory in respect of patent or proprietary medicines not having displayed on the label or container thereof the true formula or list of ingredients contained therein in a manner readily intelligible to members of the medical profession, the forms of such certificates and the fees payable therefor ;

(*c*) the procedure for preserving the secrecy of the formulae of patent or proprietary medicines when disclosed to the said Laboratory under this Act ;

(*d*) the procedure for the submission to the said Laboratory under Chapter IV of samples of drugs for analysis or test, the forms of the Laboratory's reports thereon and the fees payable in respect of such reports ;

(*e*) such other matters as may be necessary or expedient to enable the said Laboratory to carry out its functions ;

(*f*) the matters necessary to be prescribed for the purposes of the proviso to sub-section (*1*).

Drugs Consultative Committee.

7. (*1*) The Central Government may constitute an advisory committee to be called "the Drugs Consultative Committee" to advise the Central Government, the Provincial Governments and the Drugs Technical Advisory Board on any matter tending to secure uniformity throughout the Provinces in the administration of this Act.

(*2*) The Drugs Consultative Committee shall consist of two representatives of the Central Government to be nominated by that Government and one representative of each Provincial Government to be nominated by the Provincial Government concerned.

(*3*) The Drugs Consultative Committee shall meet when required to do so by the Central Government and shall have power to regulate its own procedure.

CHAPTER III.

IMPORT OF DRUGS.

Standards of quality.

8. (*1*) For the purposes of this Chapter the expression "standard quality" when applied to a drug means that the drug complies with the standard set out in the Schedule.

(*2*) The Central Government, after consultation with the Board and after giving by notification in the official Gazette not less than three months' notice of its intention so to do, may by a like notification add to or otherwise amend the Schedule for the purposes of this Chapter, and thereupon the Schedule shall be deemed to be amended accordingly.

Misbranded drugs.

9. For the purposes of this Chapter a drug shall be deemed to be misbranded—

(*a*) if it is an imitation of, or substitute for, or resembles in a manner likely to deceive, another drug, or bears upon it or upon its label or container the name of another drug, unless it is plainly and conspicuously marked so as to reveal its true character and its lack of identity with such other drug; or

(*b*) if it purports to be the product of a place or country of which it is not truly a product; or

(*c*) if it is imported under a name which belongs to another drug; or

(*d*) if it is so coloured, coated, powdered or polished that damage is concealed, or if it is made to appear of better or greater therapeutic value than it really is; or

(*e*) if it is not labelled in the prescribed manner; or

(*f*) if its label or container or anything accompanying the drug bears any statement, design or device which makes any false claim for the drug or which is false or misleading in any particular; or

(*g*) if the label or container bears the name of an individual or company purporting to be the manufacturer or producer of the drug, which individual or company is fictitious or does not exist.

Prohibition of import of certain durgs.

10. From such date as may be fixed by the Central Government by notification in the official Gazette in this behalf, no person shall import—

(*a*) any drug which is not of standard quality;

(*b*) any misbranded drug;

(*c*) any drug for the import of which a licence is prescribed, otherwise than under, and in accordance with, such licence;

(*d*) any patent or proprietary medicine, unless there is displayed in the prescribed manner on the label or container thereof either the true formula or list of ingredients contained in it in a manner readily intelligible to members of the medical profession, or the number of the certificate of registration granted in the prescribed manner in respect of such medicine by the Central Drugs Laboratory after being correctly informed of the formula of such medicine;

(*e*) any drug which by means of any statement, design or device accompanying it or by any other means, purports or claims to cure or mitigate any such disease or ailment, or to have any such other effect, as may be prescribed;

(*f*) any drug the import of which is prohibited by rule made under this Chapter:

Provided that nothing in this section shall apply to the import, subject to prescribed conditions, of small quantities of any drug for the purpose of examination, test or analysis or for personal use:

Provided further that the Central Government may, after consultation with the Board, by notification in the official Gazette, permit, subject to any conditions specified in the notification, the import of any drug or class of drugs not being of standard quality.

Explanation.—

Explanation.—The formula or list of ingredients mentioned in clause (*d*) shall be deemed to be true and a sufficient compliance with that sub-clause if, without disclosing a full and detailed recipe of the ingredients, it indicates correctly all potent or poisonous substances contained therein together with an approximate statement of the composition of the medicine.

Application of law relating to sea customs and powers of Customs officers.

11. (*1*) The law for the time being in force relating to sea customs and to goods, the import of which is prohibited by section 18 of the Sea Customs Act, 1878, (VIII of 1878.) shall, subject to the provisions of section 13 of this Act, apply in respect of drugs the import of which is prohibited under this Chapter, and officers of Customs and officers empowered under that Act to perform the duties imposed thereby on a Customs Collector and other officers of Customs, shall have the same powers in respect of such drugs as they have for the time being in respect of such goods as aforesaid.

(*2*) Without prejudice to the provisions of sub-section (*1*), the Customs Collector, or any servant of the Crown authorised by the Provincial Government in this behalf, may detain any imported package which he suspects to contain any drug the import of which is prohibited under this Chapter, and shall forthwith report such detention to the Director of the Central Drugs Laboratory and, if required by him, forward the package or samples of any suspected drug found therein to the said Laboratory.

Power of Central Government to make rules.

12. (*1*) The Central Government may, after consultation with the Board and after previous publication by notification in the official Gazette, make rules for the purpose of giving effect to the provisions of this Chapter.

(*2*) Without prejudice to the generality of the foregoing power, such rules may—

(*a*) specify the drugs or classes of drugs for the import of which a licence is required, and prescribe the form and conditions of such licences, the authority empowered to issue the same, and the fees payable therefor;

(*b*) prescribe

(*b*) prescribe the methods of test or analysis to be employed in determining whether a drug is of standard quality;

(*c*) prescribe, in respect of biological and organo-metallic compounds, the units or methods of standardisation;

(*d*) specify the diseases or ailments which an imported drug may not purport or claim to cure or mitigate and such other effects which such drug may not purport or claim to have;

(*e*) prescribe the conditions subject to which small quantities of drugs, the import of which is otherwise prohibited under this Chapter, may be imported for the purpose of examination, test or analysis or for personal use;

(*f*) prescribe the places at which drugs may be imported, and prohibit their import at any other place;

(*g*) require the date of manufacture and the date of expiry of potency to be clearly and truly stated on the label or container of any specified imported drug or class of such drug, and prohibit the import of the said drug or class of drug after the expiry of a specified period from the date of manufacture;

(*h*) regulate the submission by importers, and the securing, of samples of drugs for examination, test or analysis by the Central Drugs Laboratory, and prescribe the fees, if any, payable for such examination, test or analysis;

(*i*) prescribe the evidence to be supplied, whether by accompanying documents or otherwise, of the quality of drugs sought to be imported, the procedure of officers of Customs in dealing with such evidence, and the manner of storage at places of import of drugs detained pending admission;

(*j*) provide

(j) provide for the exemption, conditionally or otherwise, from all or any of the provisions of this Chapter and the rules made thereunder of drugs imported for the purpose only of transport through, and export from, British India;

(k) prescribe the conditions to be observed in the packing in bottles, packages or other containers of imported drugs;

(l) regulate the mode of labelling drugs imported for sale in packages, and prescribe the matters which shall or shall not be included in such labels;

(m) prescribe the maximum proportion of any poisonous substance which may be added to or contained in any imported drug, prohibit the import of any drug in which that proportion is exceeded, and specify substances which shall be deemed to be poisonous for the purposes of this Chapter and the rules made thereunder;

(n) require that the accepted scientific name of any specified drug shall be displayed in the prescribed manner on the label or wrapper of any imported patent or proprietary medicine containing such drug;

(o) provide for the exemption, conditionally or otherwise, from all or any of the provisions of this Chapter or the rules made thereunder of any specified drug or class of drugs.

Offences.

13. (1) Whoever contravenes any of the provisions of this Chapter or of any rule made thereunder shall, in addition to any penalty to which he may be liable under the provision of section 11, be punishable with imprisonment which may extend to one year, or with fine which may extend to five hundred rupees, or with both.

(2) Whoever, having been convicted under sub-section (1), is again convicted under that sub-section shall,

shall, in addition to any penalty as aforesaid, be punishable with imprisonment which may extend to two years, or with fine which may extend to one thousand rupees, or with both.

Confiscation.

14. Where any offence punishable under section 13 has been committed, the consignment of the drug in respect of which the offence has been committed shall be liable to confiscation.

Jurisdiction.

15. No Court inferior to that of a Presidency Magistrate or of a Magistrate of the first class shall try an offence punishable under section 13.

CHAPTER IV.

MANUFACTURE, SALE AND DISTRIBUTION OF DRUGS.

Standards of quality.

16. (1) For the purposes of this Chapter the expression "standard quality" when applied to a drug means that the drug complies with the standard set out in the Schedule.

(2) The Provincial Government, after consultation with the Board and after giving by notification in the official Gazette not less than three months' notice of its intention so to do, may by a like notification add to or otherwise amend the Schedule for the purposes of this Chapter, and thereupon the Schedule shall be deemed to be amended accordingly.

Misbranded drugs.

17. For the purposes of this Chapter a drug shall be deemed to be misbranded—

(a) if it is an imitation of, or substitute for, or resembles in a manner likely to deceive, another drug, or bears upon it or upon its label or container the name of another drug, unless it is plainly and conspicuously marked so as to reveal its true character and its lack of identity with such other drug; or

(b) if it purports to be the product of a place or country of which it is not truly a product; or

(c) if it is sold, or offered or exposed for sale, under a name which belongs to another drug; or

(d) if

(*d*) if it is so coloured, coated, powdered or polished that damage is concealed, or if it is made to appear of better or greater therapeutic value than it really is; or

(*e*) if it is not labelled in the prescribed manner; or

(*f*) if its label or container or anything accompanying the drug bears any statement, design or device which makes any false claim for the drug or which is false or misleading in any particular; or

(*g*) if the label or container bears the name of an individual or company purporting to be the manufacturer or producer of the drug, which individual or company is fictitious or does not exist.

Prohibition of manufacture and sale of certain drugs.

18. From such date as may be fixed by the Provincial Government by notification in the official Gazette in this behalf, no person shall himself or by any other person on his behalf—

(*a*) manufacture for sale, or sell, or stock or exhibit for sale, or distribute—

(*i*) any drug which is not of standard quality;

(*ii*) any misbranded drug;

(*iii*) any patent or proprietary medicine, unless there is displayed in the prescribed manner on the label or container thereof either the true formula or list of ingredients contained in it in a manner readily intelligible to members of the medical profession, or the number of the certificate of registration granted, in the manner prescribed by the Central Government, in respect of such medicine by the Central Drugs Laboratory after being correctly informed of the formula of such medicine;

(*iv*) any drug which by means of any statement, design or device accompanying it or by any other means, purports or claims to cure or mitigate any such disease or ailment, or to have any such other effect as may be prescribed;

(*v*) any

(*v*) any drug, in contravention of any of the provisions of this Chapter or any rule made thereunder;

(*b*) sell, or stock or exhibit for sale, or distribute any drug which has been imported or manufactured in contravention of any of the provisions of this Act or any rule made thereunder;

(*c*) manufacture for sale, or sell, or stock or exhibit for sale, or distribute any drug, except under, and in accordance with the conditions of, a licence issued for such purpose under this Chapter:

Provided that nothing in this section shall apply to the manufacture, subject to prescribed conditions, of small quantities of any drug for the purpose of examination, test or analysis:

Provided further that the Provincial Government may, after consultation with the Board, by notification in the official Gazette, permit, subject to any conditions specified in the notification, the manufacture for sale, sale or distribution of any drug or class of drugs not being of standard quality.

Explanation.—The formula or list of ingredients mentioned in sub-clause (*iii*) of clause (*a*) shall be deemed to be true and a sufficient compliance with that sub-clause if, without disclosing a full and detailed recipe of the ingredients, it indicates correctly all the potent or poisonous substances contained therein together with an approximate statement of the composition of the medicine.

19. (*1*) Save as hereinafter provided in this section, it shall be no defence in a prosecution under this Chapter to prove merely that the accused was ignorant of the nature, substance or quality of the drug in respect of which the offence has been committed or of the circumstances of its manufacture or import, or that a purchaser, having bought only for the purpose of test or analysis, has not been prejudiced by the sale. Pleas.

(*2*) For

(2) For the purposes of section 18 a drug shall not be deemed to be misbranded or to be below standard quality only by reason of the fact that—

(a) there has been added thereto some innocuous substance or ingredient because the same is required for the manufacture or preparation of the drug as an article of commerce in a state fit for carriage or consumption, and not to increase the bulk, weight or measure of the drug or to conceal its inferior quality or other defects ; or

(b) in the process of manufacture, preparation or conveyance some extraneous substance has unavoidably become intermixed with it : provided that this clause shall not apply in relation to any sale or distribution of the drug occurring after the vendor or distributor became aware of such intermixture.

(3) A person, not being the manufacturer of a drug or his agent for the distribution thereof, shall not be liable for a contravention of section 18 if he proves—

(a) that he did not know, and could not with reasonable diligence have ascertained, that the drug in any way contravened the provisions of that section, and that the drug while in his possession remained in the same state as when he acquired it ; or

(b) that he acquired the drug from a person resident in British India under a written warranty in the prescribed form and signed by such person that the drug does not in any way contravene the provisions of section 18, and that the drug while in his possession remained in the same state as when he acquired it :

Provided that a defence under clause (b) shall be open to a person only—

(i) if he has, within seven days of the service on him of the summons, sent to the Inspector a copy of the warranty with a written notice stating that he intends to rely upon it and giving

giving the name and address of the warrantor, and

(ii) if he proves that he has, within the same period, sent written notice of such intention to the said warrantor.

Government Analysts.

20. The Provincial Government may, by notification in the official Gazette, appoint such persons as it thinks fit, having the prescribed qualifications, to be Government Analysts for such areas and in respect of such drugs or classes of drugs as may be specified in the notification :

Provided that a servant of the Crown serving under the Central Government or another Provincial Government shall not be so appointed without the previous consent of the Government under which he is serving.

Inspectors.

21. (1) The Provincial Government may, by notification in the official Gazette, appoint such persons as it thinks fit, having the prescribed qualifications, to be Inspectors for the purposes of this Chapter within such local limits as it may assign to them respectively :

Provided that no person who has any financial interest in the manufacture, import or sale of drugs shall be appointed to be an Inspector under this sub-section.

(2) Every Inspector shall be deemed to be a public servant within the meaning of the Indian Penal Code, and shall be officially subordinate to such authority as the Provincial Government may specify in this behalf. (XLV of 1860.)

Powers of Inspectors.

22. Subject to the provisions of section 23 and of any rules made by the Provincial Government in this behalf, an Inspector may, within the local limits for which he is appointed,—

(a) inspect any premises wherein any drug is being manufactured and in the case of sera, vaccines and any other drug prescribed in this behalf the plant and process of manufacture and the means employed for standardising and testing the drug ;

(b) take samples of any drug which is being manufactured, or being sold or is stocked or exhibited for sale, or is being distributed ;

(c) where

(*c*) where he has reason to believe that any drug which is being manufactured for sale, or being sold or is stocked or exhibited for sale, or is being distributed, contravenes any of the provisions of section 18, order in writing the person, in whose possession such drug may be, not to dispose of any stock of such drug for a specified period not exceeding ten days, or, unless the alleged contravention is such that the defect may be removed by the possessor of the drug, seize the stock of such drug :

Provided that the Inspector shall not take any action under this clause unless he has reported the facts to the District Magistrate or the Chief Presidency Magistrate and has been authorized by such Magistrate to take such action ;

(*d*) for any of the aforesaid purposes enter at all reasonable times, with such assistants, if any, as he considers necessary, any premises wherein any drug is being manufactured, or being sold or is stocked or exhibited for sale, or is kept for distribution ;

(*e*) exercise such other powers as may be necessary for carrying out the purposes of this Chapter or any rules made thereunder. †

Procedure of Inspectors.

23. (*1*) Where an Inspector takes any sample of a drug under this Chapter, he shall tender the fair price thereof and may require a written acknowledgment therefor.

(*2*) Where the price tendered under sub-section (*1*) is refused, or where the Inspector seizes the stock of any drug under clause (*c*) of section 22, he shall tender a receipt therefor in the prescribed form.

(*3*) Where an Inspector takes a sample of a drug for the purpose of test or analysis, he shall intimate such purpose in writing in the prescribed form to the person from whom he takes it and, in the presence of such person unless he wilfully absents himself, shall divide the sample into four portions and effectively seal

seal and suitably mark the same and permit such person to add his own seal and mark to all or any of the portions so sealed and marked :

Provided that where the sample is taken from premises whereon the drug is being manufactured, it shall be necessary to divide the sample into three portions only :

Provided further that where the drug is made up in containers of small volume, instead of dividing a sample as aforesaid, the Inspector may, and if the drug be such that it is likely to deteriorate or be otherwise damaged by exposure shall, take three or four, as the case may be, of the said containers after suitably marking the same and, where necessary, sealing them.

(*4*) The Inspector shall restore one portion of a sample so divided or one container, as the case may be, to the person from whom he takes it, and shall retain the remainder and dispose of the same as follows :—

(*i*) one portion or container he shall forthwith send to the Government Analyst for test or analysis ;

(*ii*) the second he shall produce to the Court before which proceedings, if any, are instituted in respect of the drug ; and

(*iii*) the third, where taken, he shall send to the warrantor, if any, named under the proviso to sub-section (*3*) of section 19.

(*5*) Where an Inspector takes any action under clause (*c*) of section 22,—

(*a*) he shall use all despatch in ascertaining whether or not the drug contravenes any of the provisions of section 18 and, if it is ascertained that the drug does not so contravene, forthwith revoke the order passed under the said clause or, as the case may be, take such action as may be necessary for the return of the stock seized ;

(*b*) if he seizes the stock of the drug, he shall as soon as may be inform a Magistrate and take his orders as to the custody thereof ;

(*c*) without

(c) without prejudice to the institution of any prosecution, if the alleged contravention be such that the defect may be remedied by the possessor of the drug, he shall, on being satisfied that the defect has been so remedied, forthwith revoke his order under the said clause.'

Persons bound to disclose place where drugs are manufactured or kept.

24. Every person for the time being in charge of any premises whereon any drug is being manufactured or is kept for sale or distribution shall, on being required by an Inspector so to do, be legally bound to disclose to the Inspector the place where the drug is being manufactured or is kept, as the case may be.

Reports of Government Analysts.

25. (*1*) The Government Analyst to whom a sample of any drug has been submitted for test or analysis under sub-section (*4*) of section 23, shall deliver to the Inspector submitting it a signed report in triplicate in the prescribed form.

(*2*) The Inspector on receipt thereof shall deliver one copy of the report to the person from whom the sample was taken and another copy to the warrantor, if any, named under the proviso to sub-section (*3*) of section 19, and shall retain the third copy for use in any prosecution in respect of the sample.

(*3*) Any document purporting to be a report signed by a Government Analyst under this Chapter shall be evidence of the facts stated therein, and such evidence shall be conclusive unless the person from whom the sample was taken or the said warrantor has, within twenty-eight days of the receipt of a copy of the report, notified in writing the Inspector or the Court before which any proceedings in respect of the sample are pending that he intends to adduce evidence in controversion of the report.

(*4*) Unless the sample has already been tested or analysed in the Central Drugs Laboratory, where a person has under sub-section (*3*) notified his intention of adducing evidence in controversion of a Government Analyst's report, the Court may, of its own motion or in its discretion at the request either of the complainant or the accused, cause the sample of the drug produced

produced before the Magistrate under sub-section (*4*) of section 23 to be sent for test or analysis to the said Laboratory, which shall make the test or analysis and report in writing signed by, or under the authority of, the Director of the Central Drugs Laboratory the result thereof, and such report shall be conclusive evidence of the facts stated therein.

(*5*) The cost of a test or analysis made by the Central Drugs Laboratory under sub-section (*4*) shall be paid by the complainant or accused as the Court shall direct.

Purchaser of drug enabled to obtain test or analysis.

26. Any person shall, on application in the prescribed manner and on payment of the prescribed fee, be entitled to submit for test or analysis to a Government Analyst any drug purchased by him and to receive a report of such test or analysis signed by the Government Analyst.

Penalty for manufacture, sale, etc., of drugs in contravention of this Chapter.

27. Whoever himself or by any other person on his behalf manufactures for sale, sells, stocks or exhibits for sale, or distributes any drug in contravention of any of the provisions of this Chapter or any rule made thereunder shall be punishable with imprisonment which may extend to one year, or with fine which may extend to five hundred rupees, or with both.

Penalties for giving false warranty or misuse of warranty.

28. (*1*) Whoever, in respect of any drug sold by him whether as principal or agent, gives to the purchaser a false warranty that the drug does not in any way contravene the provisions of section 18 shall, unless he proves that when he gave the warranty he had good reason to believe the same to be true, be punishable with imprisonment which may extend to one year, or with fine which may extend to five hundred rupees, or with both.

(*2*) Whoever applies or permits to be applied to any drug sold, or stocked or exhibited for sale, by him, whether on the container or label or in any other manner, a warranty given in respect of any other drug, shall be punishable with imprisonment which may extend to one year, or with fine which may extend to five hundred rupees, or with both.

29. Whoever

Penalty for use of Government Analyst's report for advertising.

29. Whoever uses any report of a test or analysis made by the Central Drugs Laboratory or by a Government Analyst, or any extract from such report, for the purpose of advertising any drug, shall be punishable with fine which may extend to five hundred rupees.

Penalty for subsequent offences.

30. Whoever, having been convicted of any offence under section 27 or section 28 or section 29, is again convicted of an offence under the same section shall be punishable with imprisonment which may extend to two years, or with fine which may extend to one thousand rupees, or with both.

Confiscation.

31. Where any person has been convicted under this Chapter for contravening any such provision of this Chapter or any rule made thereunder as may be specified by rule made in this behalf, the stock of the drug in respect of which the contravention has been made shall be liable to confiscation.

Cognisance of offences.

32. (*1*) No prosecution under this Chapter shall be instituted except by an Inspector.

(*2*) No Court inferior to that of a Presidency Magistrate or of a Magistrate of the first class shall try an offence punishable under this Chapter.

(*3*) Nothing contained in this Chapter shall be deemed to prevent any person from being prosecuted under any other law for any act or omission which constitutes an offence against this Chapter.

Power of Provincial Government to make rules.

33. (*1*) The Provincial Government may, after consultation with the Board and after previous publication by notification in the official Gazette, make rules for the purpose of giving effect to the provisions of this Chapter.

(*2*) Without prejudice to the generality of the foregoing power, such rules may—

(*a*) provide for the establishment of laboratories for testing and analysing drugs;

(*b*) prescribe the qualifications and duties of Government Analysts and the qualifications of Inspectors;

(*c*) prescribe the methods of test or analysis to be employed in determining whether a drug is of standard quality;

(*d*) prescribe,

(*d*) prescribe, in respect of biological and organometallic compounds, the units or methods of standardisation;

(*e*) prescribe the forms of licences for the manufacture for sale, for the sale and for the distribution of drugs or any specified drug or class of drugs, the form of application for such licences, the conditions subject to which such licences may be issued, the authority empowered to issue the same and the fees payable therefor;

(*f*) specify the diseases or ailments which a drug may not purport or claim to cure or mitigate and such other effects which a drug may not purport or claim to have;

(*g*) prescribe the conditions subject to which small quantities of drugs may be manufactured for the purpose of examination, test or analysis;

(*h*) require the date of manufacture and the date of expiry of potency to be clearly and truly stated on the label or container of any specified drug or class of drugs, and prohibit the sale, stocking or exhibition for sale, or distribution of the said drug or class of drugs after the expiry of a specified period from the date of manufacture or after the expiry of the date of potency;

(*i*) prescribe the conditions to be observed in the packing in bottles, packages and other containers of drugs, and prohibit the sale, stocking or exhibition for sale, or distribution of drugs packed in contravention of such conditions;

(*j*) regulate the mode of labelling packed drugs, and prescribe the matters which shall or shall not be included in such labels;

(*k*) prescribe the maximum proportion of any poisonous substance which may be added to or contained in any drug, prohibit the manufacture, sale or stocking or exhibition for sale,

sale, or distribution of any drug in which that proportion is exceeded, and specify substances which shall be deemed to be poisonous for the purposes of this Chapter and the rules made thereunder ;

(l) require that the accepted scientific name of any specified drug shall be displayed in the prescribed manner on the label or wrapper of any patent or proprietary medicine containing such drug ;

(m) prescribe the form of warranty referred to in sub-section (1) of section 19 ;

(n) regulate the powers and duties of Inspectors ;

(o) prescribe the forms of report to be given by Government Analysts, and the manner of application for test or analysis under section 26 and the fees payable therefor ;

(p) specify the offences against this Chapter or any rule made thereunder in relation to which the stock of the drug shall be liable to confiscation under section 31 ;

(q) provide for the exemption, conditionally or otherwise, from all or any of the provisions of this Chapter or the rules made thereunder of any specified drug or class of drugs.

Protection to persons acting under this Chapter.

34. No suit, prosecution or other legal proceeding shall lie against any person for anything which is in good faith done or intended to be done under this Chapter.

THE SCHEDULE.

THE SCHEDULE.

(*See* sections 8 and 16.)

Standards to be complied with by imported drugs and by drugs manufactured for sale, sold, stocked or exhibited for sale, or distributed.

Class of drug.	Standard to be complied with.
1. Patent or proprietary medicines.	The formula or list of ingredients displayed in the prescribed manner on the label or container, or the formula disclosed to the Central Drugs Laboratory, as the case may be.
2. Substances commonly known as vaccines, sera, toxins, toxoids, antitoxins, and antigens and biological products of such nature.	The standards maintained at the National Institute for Medical Research, London, and such further standards of strength, quality and purity as may be prescribed.
3. Vitamins, hormones and analogous products.	The standards maintained at the National Institute for Medical Research, London, and such further standards of strength, quality and purity as may be prescribed.
4. Other drugs . .	The standards of identity, purity and strength specified in the latest edition of the British Pharmacopœia or the British Pharmaceutical Codex or any other prescribed pharmacopœia, or adopted by the Permanent Commission on Biological Standardisation of the League of Nations.

GIPD—L66 LD—28-5-40—5,000.
