# Supplementary material for: Use of Fixed Dose Combination (FDC) Drugs in India: Central Regulatory Approval and Sales of FDCs Containing Non-Steroidal Anti-Inflammatory Drugs (NSAIDs), Metformin, or Psychotropic Drugs
Source: PLoS Med. 2015 May 12;12(5):e1001826. doi: 10.1371/journal.pmed.1001826 (PMC4428752; doi:10.1371/journal.pmed.1001826)
Supplement: S3 Text — (PDF) [file pmed.1001826.s006.pdf]

Maximum price that may be charged by a retail dealer

(5)

The price specified in Column 4 PLUS a margin not exceeding Rs. 1-12-0 per cwt.

[illegible] and other local

[illegible]C-7(14)/52.]

[illegible]4 of the Supply [illegible]dification of the [illegible]stry and Supply [illegible]s to the fixation [illegible]hereby fixes the 1962·56 cwts. of [illegible]ity of Lucknow' [illegible]rmenian Street,

Maximum price that may be charged by a retail dealer

(5)

[illegible]he price specified in Column 4 PLUS [illegible] margin not exceeding Rs. [illegible]-12-0 per cwt.

[illegible] and other loca[illegible]

[illegible]-7(22)/51.]

[illegible]M, Under Secy.

MINISTRY OF FOOD AND AGRICULTURE

New Delhi, the 15th April 1952

S.R.O. 692.—In exercise of the powers conferred by clause 11 of the Sugar and Gur Control Order, 1950, the Central Government, subject to any general or special orders which may from time to time be issued by it in this behalf, is pleased to direct that the powers under clause 3 of the said Order shall also be exerciseable by the Cane Commissioner U.P. for the purpose of allowing deduction in the minimum price of sugarcane fixed under Government of India Ministry of Food and Agriculture Notification No. S.R.O. 1597 dated the 18th October 1951 for the 1951-52 crushing season, in the interest of the growers and for reasons specified under rule 20(9) of the U.P. Sugar Factories Control Rule 1938, as amended from time to time.

[No. SV-101(1-1)/51-52.]

P. A. GOPALAKRISHNAN, Jt. Secy.

AGRICULTURE

New Delhi, the 15th April 1952

S.R.O. 693.—Shri C. J. Bocarro, Assistant Secretary, Indian Central Cotton Committee, Bombay has been granted two months and fifteen days leave on average pay with effect from the 24th March, 1952 with permission to affix the 8th and 9th June, 1952 being Sunday and Public Holiday.

[No. F.1-21/52-CJ.]

S. R. MAINI, Dy. Secy.

MINISTRY OF HEALTH

New Delhi, the 14th April 1952

S.R.O. 694.—In exercise of the powers conferred by section 12 of the Drugs Act, 1940 (XXIII of 1940), the Central Government hereby direct that the following further amendments shall be made in the Drugs Rules, 1945, the same having been previously published as required by the said section, namely:—

After rule 30 of the said Rules, the following rule shall be inserted, namely:—

"30-A. (1) No new drug shall be imported except under and in accordance with the permission in writing of the licensing authority.

(2) The importer of a new drug when applying for permission shall produce before the licensing authority all documentary and other evidence relating to its standards of quality, purity and strength and such other information as may be required by the licensing authority including the results of therapeutic trials carried out with it.

Explanation.—For the purpose of this rule, 'new drug' means a drug which is not, for the time being, recognised by the Permanent Commission on Biological Standards of the World Health Organisation or in the latest edition of the British Pharmacopoeia or the British Pharmaceutical Codex or any other Pharmacopoeia recognised in this behalf by the Government of India and includes a patent or proprietary medicine containing such a drug."

[No. F.1-30/48-D.]

S. DEVANATH, Under Secy.
