# Supplementary material for: Use of Fixed Dose Combination (FDC) Drugs in India: Central Regulatory Approval and Sales of FDCs Containing Non-Steroidal Anti-Inflammatory Drugs (NSAIDs), Metformin, or Psychotropic Drugs
Source: PLoS Med. 2015 May 12;12(5):e1001826. doi: 10.1371/journal.pmed.1001826 (PMC4428752; doi:10.1371/journal.pmed.1001826)
Supplement: S4 Text — (PDF) [file pmed.1001826.s007.pdf]

MINISTRY OF HEALTH

New Delhi, the 3rd January 1961

S.O. 112.—In exercise of the powers conferred by section 12(2) of the Indian Medical Council Act, 1956 (102 of 1956), the Central Government hereby make the following amendment in the Second Schedule to the said Act, namely:—

In the said Second Schedule for the entries in column 2 against "University of Melbourne" the following entries shall be substituted, namely:—

"M.B., B.S.
M.D.
M.S."

[No. F. 17-57/59-M.1.]

New Delhi, the 7th January 1961

S.O. 113.—In exercise of the powers conferred by sub-section (4) of section 13 of the Indian Medical Council Act, 1956 (102 of 1956), the Central Government, after consulting the Medical Council of India, hereby makes the following amendments in Part II of the Third Schedule of the said Act, namely:—

In the said Part II of the Third Schedule, after the entry "Medico—Surgeon (Goa)", the following entries shall be inserted, namely:—

"M.B.B.S. (Karachi).
M.B.B.S. (Sydney—New South Wales—Australia).
M.D. (Minnesota—U.S.A.).
M.D. (Geneva—Switzerland)."

[No. F. 17-17/60-M.1.]

ORDER

New Delhi, the 3rd January 1961

S.O. 114.—With reference to the notification of the Government of India Ministry of Health No. F. 16-14/59-M.1., dated the 30th March, 1960, according recognition to the Medical qualification M.D. granted by the Baylor University, U.S.A., for the purposes of the Indian Medical Council Act, 1956 (102 of 1956), the Central Government, in exercise of the powers conferred by the proviso to sub-section (1) of section 14 of the said Act directs that the medical practice by Dr. Seaman Maynard, possessing the said qualification shall be limited to the institution of Chinchpada Mission Hospital, Chinchpada, West Khandesh for a period of two years with effect from the date of this order or so long as Dr. Seaman Maynard continues to work in the said institution for purposes of teaching, research or charitable work, whichever is shorter.

[No. F. 16-5/60-M.1.]

A. C. RAY, Under Secy.

New Delhi, the 4th January 1961

S.O. 115.—In exercise of the powers conferred by sub-section (2) of section 6, and sections 12 and 33 of the Drugs Act, 1940 (23 of 1940), the Central Government after consultation with the Drugs Technical Advisory Board, hereby makes the following rules to amend the Drugs Rules, 1945, the same having been previously published as required by the said sections, namely:—

These rules may be called the Drugs First Amendment Rules, 1961.

In the Drugs Rules, 1945,

2. (1) for the Explanation to rule 30-A, the following Explanation shall be substituted, namely:—

"*Explanation*:—For the purposes of this rule, "new drug" means a drug the composition of which is such that the drug is not generally recognised

among experts as safe for use under the conditions recommended or suggested in the label thereof and includes any drug the composition of which is such that the drug, as a result of investigations for determining its safety for use under such conditions, is so recognised, but which has not, otherwise than during the course of such investigations, been used to any large extent or for any appreciable length of time under the said conditions."

(2) in the proviso to rule 37, the following shall be inserted at the end, namely:—

"and the imports are made within a period of twelve months from the date of issue of such permission."

(3) in rule 49, clause (b) shall be omitted;

(4) in rule 65—

(i) for the first proviso to sub-rule (4), the following proviso shall be substituted, namely:—

"Provided that this sub-rule shall not apply to—

(a) the supply of drugs specified in Schedule C or Schedule E or of preparations containing drugs specified in Schedule E, on the prescription of a registered medical practitioner, or

(b) the supply of drugs specified in Schedule C by way of wholesale dealing."

(ii) after clause (c) of sub-rule (15), the following sub-rule shall be inserted, namely:—

"(16) The licensee shall maintain an Inspection Book to enable an Inspector to record his impressions and the defects noticed."

(5) in rule 74-after clause (c) the following clause shall be inserted, namely:—

(f) the licensee shall maintain an Inspection Book to enable an Inspector to record his impressions and the defects noticed."

(6) in rule 78, after clause (k), the following clause shall be inserted, namely:—

(l) the licensee shall maintain an Inspection Book to enable an Inspector to record his impressions and defects noticed."

(7) in rule 92, after clause (d), the following clause shall be inserted, namely:—

"(e) the licensee shall maintain an Inspection Book to enable an Inspector to record his impressions and defects noticed."

(8) for sub-rule (1) of rule 94, the following sub-rule shall be substituted, namely:—

"(1) Labels on packages or containers of drugs for export shall be adapted to meet the specific requirements of the law of the country to which the drug is to be exported but the following particulars shall appear in a conspicuous position on the innermost container in which the drug is packed and every other covering in which that container is packed:—

(a) name of the drug;

(b) the name, address of the manufacturer and the number of the licence under which the drug has been manufactured;

(c) batch or lot number;

(d) date of expiry, if any";

(9) to sub-rule (2) of rule 108, the following proviso shall be added, namely:—

"Provided that nothing in this sub-rule shall apply to a penicillin suspension in oil and wax."

(10) in rule 114, in clauses (g) and (h), the words "except preparations which, after being sealed in the containers, have been sterilized by heat in a manner satisfactory to the licensing authority" shall be omitted.

(11) in rule 115, for clause (a), following clause shall be substituted, namely:—

(a) to samples taken from each batch of the substance before the operation of filling and sealing the containers in which it is to be issued has commenced except preparations, which after being sealed in the containers are to be sterilized by heat in a manner satisfactory to the licensing authority; and

(12) after item (iv) of clause (b) of rule 122, the following new item shall be inserted, namely:—

"(v) the date of manufacture."

(13) for Schedule B, the following Schedule shall be substituted, namely:—

"SCHEDULE B

(*See* rules 7 and 48)

Fees for test or analysis by the Central Drugs Laboratory or the Government analyst.

I. *Fees for drugs including hormones etc. requiring biological assay.*

	Rs.
	35
Digitalis	35
Strophanthus	35
Pituitary (Posterior Lobe) Extract	35
Addrenaline preparations	35
Thyroid	50
Sex gland preparations:—	70
Ovarian	50
Luteal	50
Orchis	50
Heparin	50 to 75*
Insulin & Insulin combinations (Prolonged action)	
Organic Arsemicals:—	
Neoarsphenamine, Sulpharsphenamine and allied products	50 to 70*
Test for sterility	10
Toxicity tests for organic antimony compound	20 to 30*
Pyrogen test	20
Antibiotics (assay, pyrogen, undue toxicity, sterility; and chemical tests) (depending on the number of tests to be carried out)	30 to 70
Disinfectants	45
Surgical sutures (depending on the number of tests to be carried out)	15 to 30

II. *Fees for sera and vaccine.*

	Rs.
(i) Sera:—	
(a) Determination of exact titre	75
(b) Determination that sample is upto titre specified	50
(ii) Vaccines.	
(a) Examination in which an animal test is employed	50
(b) Examination in which an animal is not employed	25
III. *Fees for other drugs to be examined according to specifications of a Pharmacopoeia.*	20 to 30
IV. *Fees for patent and proprietary preparations.* For the assay of one ingredient	20
For each additional ingredient subject to a maximum total fee of Rs. 70*	10
V. *Crude drug* (depending on the number of tests to be carried out)	20 to 70*

*The exact amount of the fee shall be determined in case by the Director or the Government Analyst as the case may be."

(14) In Schedule F,

(a) for Section (b) in Part I, the following section shall be substituted, namely:—

"(B) Provisions applicable to the production of Vaccine Lymph (Vaccinum Vaccinae).

1. *Definition and proper Name.*—Vaccine Lymph is a preparation of the vaccinal material obtained from the vesicles produced on the skin of healthy animals by inoculation of vaccine virus. Its proper name is "Vaccine Lymph."

2. *Staff of Establishment.*—(1) The establishment in which vaccine lymph is prepared must be under the complete direction and control of a competent expert, who must be assisted by a staff adequate for carrying out the operations and tests required during the preparations of the vaccine lymph and the finished product.

(2) The entire responsibility for the production, storage, testing during manufacture and issue of safe, potent and reasonably pure vaccine lymph rests upon the competent expert.

3. *Condition and Housing of Animals.*—(1) The animals used in the production of vaccine lymph must be housed in hygienic conditions in premises satisfactory for this purpose.

(2) Only healthy animals may be used in the production of vaccine lymph. Each animal intended to be used as a source of vaccine lymph must, before being passed for the production of vaccine lymph, be subjected to a period of observation in quarantine for at least seven days. During the period of quarantine the animal must remain free from any sign of disease and must be thoroughly cleaned and groomed.

4. *Precautions to be observed in preparation.*—(1) A special room, with impervious walls and floor, which can be washed and, when necessary, chemically disinfected, must be provided for the inoculation of the animals and the collection of the vaccinal material.

(2) The inoculation shall be made on such parts of the animal as are not liable to be soiled by the passage of faeces. The surface used for inoculation shall be shaved and so cleaned as to procure the nearest possible approach to asepsis. Prior to the collection of vaccinal material the inoculated area of the skin shall be cleaned in a similar fashion.

(3) (a) Immediately before the vaccinal material is collected, the animal should be killed. Subsequently, a thorough post-mortem examination of the carcass shall be made by a qualified expert. A complete record of each such examination shall be kept and shall be open to inspection by or on behalf of the licensing authority at any time. If the examination reveals any conditions which indicate or suggest that the animal was suffering from any communicable disease (other than vaccinia) the lymph obtained from that animal shall not be issued.

(b) When lymph is collected from a living animal each such animal shall be kept under observation for a period of at least forty-eight hours after collection of lymph. If during this period the examination reveals any conditions which indicate or suggest that the animal was suffering from any infection other than vaccinia the lymph obtained from that animal shall not be issued.

(4) All instruments and appliances used in the production of vaccine lymph shall be previously subjected to an effective process of sterilization.

(5) Laboratories in which the vaccinal material is being manufactured into lymph must be housed in a building separated from stables or animal houses by a reasonable distance. Such laboratories must have impervious walls and floors and must be capable of being readily disinfected when necessary.

(6) All processes concerned with the manufacture of vaccine lymph must be carried out with thorough aseptic precautions.

(7) All vaccinal material must be subjected after collection to such treatment with glycerine or other partial disinfectant as will bring the content of bacteria and other extraneous micro-organisms of the lymph within the limit prescribed in paragraph 7 of this Part of this Schedule.

(8) (a) From the very time the vaccinal material is collected it shall be kept continuously in cold storage at a temperature below 0°C. excepting when it is

being subjected to some essential manufacturing process like grinding or treatment with glycerine or other partial disinfectant necessary to bring down the number of micro-organisms to the prescribed limit.

(b) when the procedures necessary to bring the content of bacteria and other extraneous micro-organisms within the prescribed limit have been completed, the vaccine lymph shall be kept continuously in cold storage at a temperature below 0°C., until it is withdrawn to be filled into containers for issue, after which process the filled containers shall immediately be returned to cold storage and kept continuously at a temperature below 0°C, until required for issue:

Provided that it shall be permissible to remove vaccine lymph from one such cold store to another, if adequate precautions are taken during such removal to guard against deterioration.

(c) A four-hourly record of daily temperature of the cold room shall be maintained for inspection.

5. *Containers.*—Vaccine lymph for issue shall be introduced with aseptic precautions either—

(a) into previously sterilized capillary glass tubes, each of which thereafter has been hermetically sealed at each end, and contains a quantity of vaccine lymph suitable for the effective vaccination of one human subject; or

(b) into tubes or containers of larger dimensions which have been sterilized before the introduction of the lymph and sealed so as to preclude the access of bacteria.

6. *Labelling.*—(1) The label on the container or a label or wrapper affixed to the package in which the container is issued for sale, shall bear a statement that the potency of the vaccine lymph cannot be assured for more than seven days from the date of completion of manufacture, unless the lymph is kept continuously at a temperature below 10°C. when the potency can be assured for fourteen days; Provided that it shall be permissible to state that if the lymph is kept continuously below 0°C. the potency can be assured for at least six months.

(2) For the purpose of clause (b) of sub-rule (3) of rule 109 the date on which the manufacture of the batch is completed shall be the date on which the vaccine lymph is removed for issue from cold storage after having been kept continuously at a temperature below 0°C, since the date of filling into containers for issue.

7. *Tests for Purity.*—(1) The vaccinal material shall be exposed to the action of glycerine or other partial disinfectant under suitable conditions of temperature until tests made by means of plate cultures have shown that the total number of living bacteria or other extraneous micro-organisms has been reduced to not more than 5 in 1 milligram, or 5,000 in 1 ml. of the vaccine lymph. The results of these tests shall be recorded and the records kept for inspection. The determination of the content of the living micro-organisms in the vaccine lymph shall be made in a manner approved by the licensing authority and the enumeration of colonies shall be made after incubation for two days at approximately 37°C., and then for at least three days at approximately 20°C.

(2) (a) Tests for the detection of *B. anthracis, Cb. tetani, Streptococcus haemolyticus, B. coli, Staphylococci* or any other pathogen which may prove harmful if introduced into the body by the process of vaccination shall be performed in a manner approved by the licensing authority and a record kept for inspection.

(b) if *B. anthracis Cl tetani* or *Streptococcus haemolyticus* is found to be present in the vaccine lymph at any stage of its preparation, either before or after the prescribed reduction in the number of living micro-organisms has been effected the batch of lymph shall be rejected forthwith. But if *B. Coli* or any other pathogen which may prove harmful if introduced into the body by the process of vaccination is found, the lymph must pe kept in cold storage till an examination of at least 10 milligrams or 0.01 ml. of the lymph fails to reveal its presence.

(c) The test for the detection of various harmful organisms in vaccine lymph shall be performed both at the initial and final stages of its preparation.

(3) When the prescribed reduction in the number of living micro-organisms has been effected the batch of vaccine lymph may be issued if—

(a) tests carried out in a manner approved by the licensing authority on a sample not less than 10 milligrams or 0.01 ml. have failed to reveal the presence of *B. anthracis*; and

(b) tests carried out in a manner approved by the licensing authority on a sample of not less than 0·1 per cent. of the batch have failed to reveal the presence of *Cl tetani*; and

(c) tests carried out after the process of purification has been completed on a sample of not less than 10 milligrams or 0.01 ml. have failed to reveal the presence of beta haemolytic streptococci; and

(d) tests carried out in a manner approved by the licensing authority on a sample in which not less than 3 ml. of lymph is injected under the skin or in the peritoneal cavity of a healthy guineapig or 5 ml. under the skin or in peritoneal cavity of a rabbit and/or 0.5 ml. under the skin of a healthy mouse fail to produce serious symptoms or death of the animals.

8. *Tests for Potency.*—(1) Each batch of vaccine lymph, after the process of purification has been completed, shall be tested for potency so as to ensure its activity at the time of issue. These tests shall be applied not more than three months before the batch of lymph is finally issued.

(2) For the purpose of a test for potency a dilution shall be prepared by mixing 1 volume of the lymph with 1,000 volumes of physiological saline solution, or other suitable diluent. The dilution shall be used for the test without filtration.

(3) Such dilution of the vaccine lymph shall be tested by application to the suitably prepared skin of a rabbit and the batch of vaccine lymph from which the dilution was prepared shall not be issued unless the lesions characteristic of vaccinia are produced in a susceptible animal. For the purpose of comparison a similar dilution of lymph of known potency shall be applied simultaneously to the skin of the same animal: Provided that the licensing authority may approve any other form of comparative test for potency which may be submitted to the licensing authority for approval".

(b) In Part IX, after the entry 4 under "ANY OTHER PREPARATION IN A FORM TO BE ADMINISTERED PARENTERALLY" the following shall be added, namely:—

"LIVER INJECTION CRUDE.

1. Liver Injection Crude is a sterile solution in water for injection of that soluable thermostable fraction of mammalian livers which increases the red blood corpuscles in the blood of persons suffering from pernicious and other types of maccocytic anaemias. It is obtained by stopping the processes of extraction at such a stage that the final product is derived directly from an alcohol solution of a concentration not higher than 70 per cent. by volume, of C_2H_5 OH.

Each ml. of Liver Injection Crude has Vitamin B_{12}, activity equivalent to either 1 microgram or 2 micrograms of cyanocobalamin. The preparation shall contain not more than 0·5 per cent. of cresol or of phenolas a bacteriostatic agent.

2. *Proper name.*—The proper name of the preparation shall be "Liver Injection Crude.

3. *Description.*—A brownish liquid which at times may show a slight turbidity.

4. *Tests:*—

(a) *Reaction:* pH. 5 to 7.

(b) *Total Solids.*—Evaporate to dryness in a water bath at 105° C. for an hour and then at 60° C. in vacuum for 2 hours; cool in a desiccator and weigh. The total solids shall not be less than 15 per cent. w/v. in the case of preparation containing 2 microgram of cyanocobelamin per ml. and 7.5 per cent. w/v. in the case of preparation containing 1 microgram of cyanocobelamin per ml. respectively.

(c) *Limits for proteins.*—The protein nitrogen shall not exceed 0.08 per cent. w/v. as determined by precipitating the proteins with an equal volume of 20 per cent. trichloroacetic acid washing the precipitate with 10 per cent. trichloroacetic acid and by estimating the nitrogen content of the precipitate by the Micro-Kjeldahl method.

(d) *Absence of undue toxicity.*—The test should be performed on a batch of 5 healthy white mice weighing between 17 and 22 g. Intraperitoneal injection of the sample in dosage of 0.25 ml. per 20 g. of body weight shall not cause death within a period of 120 hours of any of the 5 mice tested. If even one of the 5 mice dies, the test shall be repeated and if there is no mortality in the second batch within a period of 120 hours the sample shall be deemed to have passed the test.

(e) *Sterility and pyrogen test.*—Liver Injection Crude shall comply with the sterility and pyrogen tests laid down for "Injections" in the Indian Pharmacopoeia. The dose to be injected into rabbits for the pyrogen test shall be 0.5 ml. per kilogram body weight.

(f) *Potency.*—The potency shall be determined by the microbiological method for the estimation of vitamin B_{12} activity as specified in the Indian Pharmacopoeia and shall be not less than 100 per cent. and not more than 150 per cent. of that stated on the label.

5. *Container.*—The container used for Liver Injection Crude shall comply with the requirements laid down in the Indian Pharmacopoeia for container of 'Injections'.

6. *Storage.*—Liver Injection Crude shall be stored in a cool place preferably at a temperature not exceeding 20° C, and protected from light.

7. *Label.*—The label on the container shall state the following details in addition to any o'her particulars prescribed in these Rlues:—

(1) The amount of Vitamin B_{12} activity (Cyanocobalamin) per ml.

(2) The average amount of raw liver processed to produce 1 ml. of the extract.

(3) The date of expiration of potency which should not be later than 24 months from the da'e of manufacture, and

(4) The name and quantity of the bacteriostatic agent added.

[No. F. 1-45/58-D.]

M. K. KUTTY, Dy. Secy.

CORRIGENDUM

New Delhi, the 3rd January 1961

S.O. 116.—In the 5th line of the Ministry of Health Notification No. F.14-22/58-IH, dated the 22nd August, 1960, the words "Deratisation Certificates" may be inserted between the words "Issue of" and "Deratisation".

[No. F. 14-22/58-IH.]

[No. D. 7169-IH/60.]

T. V. ANANTANARAYANAN, Under Secy.

MINISTRY OF TRANSPORT & COMMUNICATIONS

(Department of Transport)

(Transport Wing)

MERCHANT SHIPPING

New Delhi, the 5th January 1961

S.O. 117.—In pursuance of sub-section (1) of section 151 of the Indian Merchant Shipping Act, 1923 (21 of 1923), the Central Government hereby makes
