# Supplementary material for: Use of Fixed Dose Combination (FDC) Drugs in India: Central Regulatory Approval and Sales of FDCs Containing Non-Steroidal Anti-Inflammatory Drugs (NSAIDs), Metformin, or Psychotropic Drugs
Source: PLoS Med. 2015 May 12;12(5):e1001826. doi: 10.1371/journal.pmed.1001826 (PMC4428752; doi:10.1371/journal.pmed.1001826)
Supplement: S5 Text — (PDF) [file pmed.1001826.s008.pdf]

(Department of Agriculture)

(Indian Council of Agricultural Research)

New Delhi, the 14th June, 1961.

S.O. 1448.—In pursuance of the appropriate provision of the Indian Cotton Cess Act, 1923 (14 of 1923), the Central Government are pleased to nominate the following persons to be members of the Indian Central Cotton Committee, Bombay, for a period of three years with effect from 1st April, 1961.

S. No.	Name and address	Section
1.	Shri G.K. Devarajulu, Managing Agent, the Laxmi Mills Ltd., Coimbatore.	4(v)
2.	Shri B.M. Patil, B.Sc., Farmer, Torvi, Bijapur Distt.	4(x)

[No. 1-4/61-Com.IV.]

SANTOKH SINGH, Under Secy.

MINISTRY OF HEALTH

New Delhi, the 13th June 1961

S.O. 1449.—In exercise of the powers conferred by sections 12 and 33 of the Drugs Act, 1940 (23 of 1940), the Central Government hereby makes the following rules further to amend the Drugs Rules, 1945, the same having been previously published as required by the said sections, namely:—

1. These rules may be called the Drugs Third (Amendment) Rules, 1961.

In the Drugs Rules (hereinafter referred to as the principal rules)—

1. after rule 54 the following rule shall be inserted, namely:—

"54-A *Prohibition of sale.*—No person in possession of a drug in respect of which an Inspector has made an order under clause (c) of sub-section (i) of section 22 of the Act shall in contravention of that order sell or otherwise dispose of any stock of such drug."

2. In the principal Rules, for rule 58, the following rule shall be substituted, namely:—

"58. *Confiscation of drugs.*—Where any person is convicted for contravening any of the provisions of Chapter IV of the Act or any rule made thereunder the stock of the drug in respect of which the contravention has been made shall be liable for confiscation.

3. In rule 59 of the principal Rules,

(a) in sub-rule (2) for the existing proviso, the following shall be substituted, namely:—

"Provided that in the case of an itinerant vendor or an applicant who desires to establish a shop in a village or town having a population of 5,000 or less the application in form 19-A shall be accompanied by a fee of rupees five."

(b) for sub-rule (3) the following shall be substituted, namely:—

"(3) A fee of rupees five and in the case of an itinerant vendor or an applicant who desires to establish a shop in a village or town having a population of 5,000 or less, a fee of rupee one and twenty-five naye paise shall be paid for a duplicate copy of a licence issued under this rule, if the original is defaced, damaged or lost;

"Provided that if the applicant applies for the renewal of a licence after its expiry but within one month of such enquiry the fee payable for renewal of such licence shall be rupees twenty plus an additional fee of rupees twenty, and in the case of itinerant vendor or an applicant desiring to open a shop in village or town having a population of 5,000 or less the fee shall be rupees five plus an additional fee of rupees five."

4. In rule 63 of the principal Rules, for the existing proviso, the following proviso shall be substituted, namely:—

"Provided that if the application for renewal of a licence in force is made before its expiry, or if the application is made and the additional fee paid within one month of its expiry the licence shall continue to be in force until orders are passed on the application. The licence shall be deemed to have expired if application for its renewal is not made within one month after its expiry."

5. In sub-rule (2) of rule 64 of the principal Rules, in clause (i) the words letters and figures 'the 1st April, 1950' shall be omitted.

6. In the principal Rules, in the explanation to sub-rule (15) of rule 65, after clause (b) the following clause shall be inserted, namely:—

'(bb) is a registered pharmacist under the Pharmacy Act, 1948, or'.

7. In rule 69 of the principal Rules, for the sub-rule (3) the following sub-rule shall be substituted, namely:—

"(3) If a person applies for the renewal of a licence after its expiry but within one month of such expiry the fee payable for the renewal of such licence shall be in the case of Form 24-B, rupees forty plus an additional fee of rupees twenty and, in the case of Form 24, rupees two hundred plus an additional fee of rupees one hundred.

8. After rule 69-A of the principal Rules, the following rule shall be inserted, namely:—

"69-B. Applications to manufacture 'new drug' other than the drugs classifiable under Schedule C and C(1) products.—Subject to the other provisions of these Rules,

(i) no 'new drug' shall be manufactured unless it is previously approved by the licensing authority mentioned in rule 21;

(ii) the manufacturer of a 'new drug' when applying for approval to the licensing authority mentioned in sub-rule (i) shall produce all documentary and other evidence relating to its standards of quality, purity and strength and such other information as may be required including the results of therapeutic trials carried out with it;

(iii) while applying for a licence to manufacture a 'new drug' or its preparations an applicant shall produce along with his application evidence that the drug for the manufacture of which application is made has already been approved.

Explanation.—In this rule 'new drug' has the same meaning as in rule 30-A."

9. In rule 71 of the principal rules, in sub-rule (1)—

(i) in clause (c), the word 'or' shall be inserted at the end;

(ii) after clause (c), the following clause shall be inserted, namely:—

"(d) holding any foreign qualification the quality and content of training of which are comparable with those prescribed in clause (a), clause (b) or clause (c) and is permitted to work as competent technical staff under this rule by the Central Government."

10. In the principal Rules, for the proviso to rule 72, the following shall be substituted, namely:—

"Provided that if application for the renewal of a licence is made before its expiry, or if the application is made within one month of its expiry after payment of the additional fee, the licence shall continue to be in force until orders are passed on the application. The licence shall be deemed to have expired if application for its renewal is not made within one month after its expiry."

11. In rule 75 of the principal Rules, for the proviso to sub-rule (1), the following proviso shall be substituted, namely:—

"Provided that if the applicant applies for the renewal of a licence after its expiry but within one month of such expiry the fee payable for renewal of the licence shall be rupees three hundred plus an additional fee of rupees two hundred in addition to the inspection fee."

(2) After rule 75-A of the principal rules the following rule shall be inserted, namely:—

"75-B. Applications to manufacture new drugs classifiable under Schedules C and C(1)

Subject to the other provisions of these Rules,

(i) no 'new drug' shall be manufactured unless it is previously approved by the 'Licensing authority' mentioned in rule 21;

(ii) the manufacturer of a 'new drug' when applying for approval to the licensing authority mentioned in sub-rule (i) shall produce all documentary and other evidence relating to its standards of quality, purity and strength and such other information as may be required including the results of therapeutic trials carried out with it;

(iii) while applying for a licence to manufacture a 'new drug' or its preparations an applicant shall produce along with his application evidence that the drug for the manufacture of which application is made has already been approved.

Explanation.—In this rule 'new drug' has the same meaning as in rule 30-A."

13. In rule 76 of the principal rules, after clause (c), the following clauses shall be inserted, namely:—

"(d) a graduate in Chemical Engineering of a University recognised by the Central Government with at least three years' practical experience in the manufacture of drugs to which this licence applies after his graduation; or

(e) holding any foreign qualification the quality and content of training of which are comparable with those prescribed in clause (a), clause (b), clause (c) or clause (d) and is permitted to work as competent technical staff under this rule by the Central Government."

14. In the principal rules, for the proviso to rule 77, the following proviso shall be substituted, namely:—

"Provided that if application for the renewal of a licence in force is made before its expiry, or if the application is made within one month of its expiry after payment of the additional fee, the licence shall continue to be in force until orders are passed on the application. The licence shall be deemed to have expired if application for its renewal is not made within one month after its expiry."

15. To rule 89 of the principal rules, the following proviso shall be inserted, namely:—

"Provided that in the case of a drug the composition of which is such that the drug is not generally recognised among experts qualified by scientific training and experience to evaluate the safety of drugs as safe for use, no licence in Form 29 shall be granted unless the applicant produces a certificate from the "Licensing authority" mentioned in rule 21, to the effect that there would be no objection to such licence being granted."

16. In rule 94 of the principal Rules, for sub-rule (2), the following sub-rule shall be substituted, namely:—

"(2) The provisions of rules 96 to 101 inclusive, shall not apply to a medicine made up ready for treatment, whether after or without dilution, which is supplied on the prescription of a registered medical practitioner provided that:—

(i) the medicine is labelled with the following particulars:—

(a) The name and address of the supplier;

(b) The name of the patient and the quantity of the medicine;

(c) The number representing serial number of the entry in the prescription register;

(d) The dose, if the medicine is for internal use;

(e) The words 'FOR EXTERNAL USE ONLY' if the medicine is for external application, and the words "POISON" and "FOR EXTERNAL USE ONLY" in the manner prescribed in rule 98 if the medicine is for external use and contains a substance specified in Schedule E;

(ii) Condition (3) of the conditions in rule 65 is satisfied."

17. In rule 96 of the principal rules, for sub-rule (i) the following sub-rule shall be substituted, namely:—

"96. *Manner of labelling.*—(1) Subject to the other provisions of these Rules, the following particulars shall be either printed or written in indeliable ink and shall appear in a conspicuous manner on the label of the innermost container of any drug and on every other covering in which the container is packed.

(i) *The name of the drug.*

For this purpose, the name shall be given in an equally conspicuous manner as the trade name, if any, and shall be,

(a) for drugs included in the pharmacopoeias specified in the Schedule to the Act or rule 124, the name or synonym specified in the respective pharmacopoeia followed by the letters "IP", "BP", "BPC", "USP", "NF", "Ph.I", "USSRP" as the case may be;

(b) for other drugs, the approved scientific name where known, or if not known the name descriptive of the true nature and origin of the substance.

(ii) A correct statement of the net content in terms of weight, measure, number or Units of activity as the case may be. The weight and volume shall be expressed in Metric system.

(iii) *The content of active ingredients.*—This shall be expressed.

(a) for oral liquid preparations in terms of the content per single dose, the dose being indicated in millilitres;

(b) for liquid parenteral preparations in terms of 1 millilitre or percentage by volume;

Provided that if the preparation is contained in an ampoule it will be enough if the composition is shown on the label or wrapper affixed to any package in which such ampoule is issued for sale;

(c) for tablets, capsules, pills and the like, in terms of the content in each tablet, capsule, pill or other unit, as the case may be;

(d) for other preparations in terms of percentage by weight or volume or in terms of Unitage per grame or millilitre as the case may be.

Provided that sub-rule (iii) shall not apply to a pharmacopoeial preparation where the composition of such preparation is specified in the respective pharmacopoeia.

(iv) Name and address of the manufacturer.

Provided that if the drug is contained in an ampoule, it shall be enough if only the name of the manufacturer and his principal place of business is shown.

(v) Every drug manufactured in India shall bear on its label a distinctive batch number, that is to say, the number by reference to which details of manufacture of the particular batch from which the substance in the container is taken are recorded and are available for inspection; the figure representing the batch number being preceded by the words "Batch No." or "Batch" or "Lot Number" "Lot No." or "Lot" or any distinguishing prefix.

(vi) Every drug manufactured in India shall bear on its label the number of the licence under which the drug is manufactured, the figure representing the manufacturing licence number being preceded by the words "Manufacturing Licence Number" or 'Mfg. Lic. No.' or 'M.L.' ";

18. In rule 100 of the principal rules, for the words "Labelling with the name of the substance", the words and letter "Labelling with the name of substance specified in Schedule E" shall be substituted.

19. In rule 101 of the principal rules, for the words "Labelling with the statement of quantity" the words and letter "Labelling with the statement of quantity of alcohol or a substance specified in Schedule E" shall be substituted.

20. In rule 121-A of the principal rules, the words 'absence of' wherever they occur shall be omitted.

21. In the principal rules, in form 19A, for the note under the asterick, the following shall be substituted, namely:—

"Rupees five for itinerant vendors and applicants from a village or town having a population of 5,000 or less, and rupees twenty for other restricted licence".

2. In Schedule C to the principal rules, for item (12), the following item shall be substituted, namely:—

"(12) Any other preparation which is meant for parenteral administration either in the form in which it is marketed or after being made up with suitable solvent or medium, and which—

(a) requires to be stored in a refrigerator, or

(b) does not require to be stored in a refrigerator."

23. In part IX of Schedule F to the principal rules, in paragraph 3A, the words "absence of" shall be omitted.

24. In Schedule K to the principal rules, after entry 12, the following entry shall be inserted, namely:—

	Extent of exemption
"13. The following household remedies, namely:<br>(a) Castor oil<br>(b) Lozenges, pills and tablets for cough<br>(c) Eucalyptus oil<br>(d) Ointments including analgesic balms<br>(e) Quinine tablets	The provisions of Chapter IV of the Act and the Rules thereunder which require them to be covered with a sale licence in form 20-A subject to the following conditions.<br>(a) The drugs are sold only in a village having population of not more than one thousand persons and where there is no licensed dealer under the Drugs Act;<br>(b) the drugs do not contain any substance specified in Schedules E and L;<br>(c) the drugs are sold in the original unopened containers of the licensed manufacturers;<br>(d) when the drugs are sold under clause (a) condition 3 under "conditions of licence" of Form 20-B shall not apply."

[No. F. 1-19/59-D.]

BESHASHAR NATH, Under Secy.

New Delhi, the 14th June 1961

S.O. 1450.—In pursuance of item (30) of Part II of the Schedule to the Dentists Act, 1948, (16 of 1948), the Dental Council of India hereby approves the following foreign qualification, namely:—

"The Diploma Ecole de Chirugie Dentaire et de Stomatologie de Paris".

S. BRATT, Secy.

Dental Council of India.

[No. F.3-18/61-MII.]
