# Supplementary material for: Use of Fixed Dose Combination (FDC) Drugs in India: Central Regulatory Approval and Sales of FDCs Containing Non-Steroidal Anti-Inflammatory Drugs (NSAIDs), Metformin, or Psychotropic Drugs
Source: PLoS Med. 2015 May 12;12(5):e1001826. doi: 10.1371/journal.pmed.1001826 (PMC4428752; doi:10.1371/journal.pmed.1001826)
Supplement: S6 Text — (PDF) [file pmed.1001826.s009.pdf]

15. सा. का. नि. 510(अ) तारीख 26-7-82
16. सा. का. नि. 14(अ) तारीख 7-1-83
17. सा. का. नि. 318 (अ) तारीख 1-5-84
18. सा. का. नि. 331(अ) तारीख 8-5-84
19. सा. का. नि. 460(अ) तारीख 20-6-84
20. सा. का. नि. 487(अ) तारीख 2-7-84
21. सा. का. नि. 89(अ) तारीख 16-2-85
22. सा. का. नि. 788 तारीख 10-10-85
23. सा. का. नि. 17(अ) तारीख 7-1-86
24. सा. का. नि. 1049(अ) तारीख 29-8-86
25. सा. का. नि. 1960(अ) तारीख 30-9-86
26. सा. का. नि. 1115(अ) तारीख 30-9-95
27. सा. का. नि. 71(अ) तारीख 30-1-87
28. सा. का. नि. 570(अ) तारीख 12-6-87
29. सा. का. नि. 626(अ) तारीख 2-7-87
30. सा. का. नि. 792(अ) तारीख 17-9-87
31. सा. का. नि. 371(अ) तारीख 24-3-88
32. सा० का० नि० 676(अ) तारीख 2-6-88
33. सा० का० नि० 677(अ) तारीख 2-6-88
34. सा० का० नि० 676(अ) तारीख 2-6-88
35. सा० का० नि० 681(अ) तारीख 6-6-88
36. सा० का० नि० 735(अ) तारीख 24-6-88
37 सा० का० नि० 813(अ) तारीख 27-7-88
38. सा० का० नि० 854(अ) तारीख 12-8-88

(शुद्धिपत्र)

MINISTRY OF HEALTH AND FAMILY WELFARE

(Department of Health)

New Delhi, the 21st September, 1988

NOTIFICATION

G.S.R. 944 (E).—Whereas a draft of certain rules further to amend the Drugs and Cosmetics Rules, 1945, was published, as required by sections 12 and 33 of the Drugs and Cosmetics Act, 1940 (23 of 1940), under the notification of the Government of India in the Ministry of Health and Family Welfare, No. GSR 602 (E), dated the 26th June, 1987, in the Gazette of India, Extraordinary, Part II, Section 3, Sub-section (i), dated the 26th June, 1987, inviting objections and suggestions from all persons likely to be effected thereby before the expiry of a period of thirty days from the date on which the copies of the Official Gazette containing the said notification were made available to the public;

And whereas the said Gazette was made available to the public on the 12th August, 1987;

And whereas the objections and suggestions received from the public on the said draft rules have been considered by the Central Government;

Now, therefore, in exercise of the powers conferred by sections 12 and 33 of the Drugs and Cosmetics Act, 1940 (23 of 1940), the Central Government, after consultation with the Drugs Technical Advisory Board, hereby makes the following rules further to amend the Druge and Cosmetics Rules, 1945, namely :—

1. (1) These rules may be called the Drugs and Cosmetics (Eight Amendment) Rules, 1988.

(2) They shall come into force on the date of their publication in the Official Gazette.

2. In the Drugs and Cosmetics Rules, 1945 (hereinafter referred to as the said rules), rules 30A, 69B and 75B shall be omitted.

3. After Part X of the said rules, the following new Part XA shall be inserted, namely :—

"Part-XA-Import or manufacture of new drug for clinical trials or marketing.

122A. Application for permission to Import New Drug.

(1) No new drug shall be imported except under and in accordance with the permission in writing of the licensing authority defined in clause (b) of rule 21.

(2) The importer of a new drug when applying for permission under sub-rule (1), shall submit data as given in Appendix I to Schedule Y including the results of local clinical trials carried out in accordance with the guidelines specified in that Schedule and submit the report of such clinical trials in the format given in Appendix II to the said Schedule.

Provided that the requirement of submitting the results of local clinical trials may not be necessary if the drug is of such a nature that the licensing authority may, in public interest decide to grant such permission on the basis of data available from other countries.

Provided further that the submission of requirements relating to Animal Toxicology, Reproduction studies, Teratogenic studies, perinatal studies, Mutagencity and Carcinogenicity may be modified or relaxed in case of new drugs approved and marketed for several years in other countries if he is satisfied that there is adequate published evidence regarding the safety of the drug, subject to the other provisions of these rules.

122B Application for approval to manufacture New Drug other than the Drugs classifiable under Schedules C and C (1) :

(1) No new drug other than the drug classifiable under Schedules C and C (I) shall be manufactured unless it is approved by the licensing authority defined in rule 21 :

(2) The manufacturer of a new drug under sub-rule (1) when applying for approval to the licensing authority mentioned in the said sub-rule, shall submit data as given in Appendix I to ScheduleY including the results of clinical trials carried out in the country in accordance with the guidelines specified in Schedule Y and submit the report of such clinical trials in the format given in Appendix II to the said Schedule:

(3) When applying for approval to manufacture of a new drug under sub-rule (1) or its preparations, to the State Licensing Authority, an applicant shall produce along with his application, evidence that the drug for the manufacture of which application is made has already been approved by the licensing authority mentioned in rule 21 :

Provided that the requirement of submitting the result of local clinical trials may not be necessary if the drug is of such a nature that the licensing authority may, in public interest decided to grant such permission on the basis of data available from other countries :

Provided further that the submission of requirements relating to Animal Toxicology, Reproduction studies, Teratogenic studies, Perinatal studies, Mutagenicity and Carcinogenicity may be modified or relaxed in case of new drugs approved and marketed for several years in other countries if he is satisfied that there is adequate published evidence regarding the safety of the drug, subject to the other provisions of these rules.

122C. Application for approval to manufacture new drug clasifiable under Schedules C and C (1)

(1) No new drug classifiable under Schedule C and C (L) shall be manufactured unless it is previously approved by the licensing authority mentioned in rule 21 :

(2) A manufacturer of a new drug under sub-rule (1) when applying for approval to the licensing authority mentioned in sub-rule (1) shall submit data as given in Appendix I to Schedule Y including the results of clinical trials carried out in country as per format given in Appendix II to Schedule Y :

(3) While applying for approval to manufacture a new drug under sub-rule (1) or its preparations to the State Licensing Authority an applicant shall produce along with his application evidence that the drug for the manufacture of which application is made, has already been approved by the licensing authority mentioned in rule 21 :

Provided that the requirement of submitting the results of local clinical trials may not be necessary if the drug is of such a nature that the licensing authority may, in public interest decide to grant such permission on the basis of data available from other countries :

Provided further that the submission of requirements relating to Animal Toxicology, Reproduction studies, Teratogenic studies, Perinatal studies, Mutagenicity and Carcinogenicity may be modified or relaxed in case of new drug approved and marketed for several years in other countries if he is satisfied that there is adequate published evidence regarding

the safety of the drug, subject to the other provisions of these rules :

122D. Application for permission to import or manufacture fixed dose combination of drug :

Application for permission to import or manufacture fixed dose combinations of drugs already approved as individual drugs, by the licensing authority mentioned in rule 21, shall accompany information and data as given in Appendix VI of Schedule Y.

122E. Definition of new drug :

For the purpose of this part, new drug shall mean and include :—

(a) A new substance of chemical, biological or biotechnological origin; in bulk or prepared dosage form ; used for prevention, diagnosis, or treatment of disease in man or animal ; which, except during local clinical, trials, has not been used in the country to any significant extent ; and which, except during local clinical trials, has not been recognized in the country as effective and safe for the proposed claims.

(b) A drug already approved by the licensing authority mentioned in rule 21 for certain claims, which is now proposed to be marketed with modified or new claims, namely, indications, dosage, dosage form (including sustained release dosage form) and route of administration.

(c) A fixed dose combination of two or more drugs, individually approved earlier for certain claims, which are now proposed to be combined for the first time in a fixed ratio, or if the ratio of ingredients in an already marketed combination is proposed to be changed, with certain claims, viz indications, dosage, dosage form (including sustained release dosage form) and route of administration. (See item (b) and (c) of Appendix IV to Schedule Y).

Explanation :—For the purpose of this rule —

(i) all vaccines shall be new drugs unless certified otherwise by the licensing authority under rule 21 ;

(ii) a new drug shall continue to be considered as new drug for a period of four years from the date of its first approval or its inclusion in the Indian Pharmacopoeia whichever is earlier.

4. After Schedule X of the said rules, the following Schedule shall be inserted, namely :—

"SCHEDULE—Y

REQUIREMENT AND GUIDELINES ON CLINICAL TRIALS FOR IMPORT AND MANUFACTURE OF NEW DRUG

1. Clinical Trials

1.1 Nature of trials :—The clinical trials required to be carried out in the country before a new drug is approved for marketing depend on the status of the drug in other countries. If the drug is already approved/marketed, phase III trials as required under item 7 of Appendix I usually are required. If the drug is not approved/marketed, trials are generally allowed to be initiated at one phase earlier to the phase of trials in other countries.

For new drug substances discovered in other countries phase I trials are not usually allowed to be initiated in India unless phase I data as required under item 5 of the said Appendix from other countries are available. However, such trials may be permitted even in the absence of phase I data from other countries if the drug is of special relevance to the health problem of India.

For new drug substances discovered in India, clinical trials are required to be carried out in India right from phase I as required under item 5 of the said Appendix through phase III as required under item 7 of the said Appendix, permission to carry out these trials is generally given in stages, considering the data emerging from earlier phase.

1.2 Permission for trials :—Permission to initiate clinical trials with a new drug may be obtained by applying in Form 12 for a test licence (TL) to import or manufacture the drug under the Rules. Data appropriate for the various phases of clinical trials to be carried out should accompany the application as per format given in Appendix I (item 1-4). In addition, the protocol for proposed trials, case report forms to be used, and the names of investigators and institutions should also be submitted for approval. The investigators selected should possess appropriate qualifications and experience and should have such investigational facilities as are germane to the proposed tirals protocol.

Permission to carry out clinical trials with a new drug is issued along with a test licence in Form 11.

It is desirable that protocols for clinical trials be reviewed and approved by the institution's ethical committee. Since such committees at present do not exist in all institutions the approval granted to a protocol by the ethical committee of one institution will be applicable to the use of that protocol in other institution which do not have an ethical committee. In case none of the trial centres/institutions has an ethical committees the acceptance of the protocol by the investigator and its approval by the Drugs Controller (India) of any officer as authorised by him to do so will be adequate to initiate the trials.

For new drugs having potential for use in children, permission for clinical trials in the paediatric age group is normally given after phase III trials as required under item 7 of the said Appendix in adults are completed. However, if the drug is of value primarily in a disease of children, early trials in the paediatric age group may be allowed.

1.3 Responsibilities of Sponsor|Investigator :— Sponsors are required to submit to the licensing authority as given under rule 21 an annual status report on each clinical trials, namely, ongoing, completed, or terminated. In case a trial is terminated, reason for this should be stated. Any unusual, unexpected, or serious adverse drug reaction (ADR) detected during a trial should be promptly communicated by the sponsor to the licensing authority under rule 21 and the other investigators.

In all trials an informed, written consent required to be obtained from each volunteer|patient in the prescribed Forms (See Appendix V) which must be signed by the patient|volunteer and the chief investigator.

2. Chemical and pharmaceutical information. Most of the data under this heading (see Appendix I, item 2) are required with the application for marketing permission. When the application is for clinical trials only, information covered in item 2.1 to 2.3 of Appendix I will usually suffice.

3. Animal Toxicology.

3.1 Acute Toxicology :—Acute toxicity studies (See Appendix I item 4..2) should be carried out in at least two species usually mice and rats using the same route as intended for humans. In addition, at least one more route should be used to ensure systemic absorption of the drug; this route may depend on the nature of the drug. Mortality should be looked for upto 72 hours after parenteral administration and upto 7 days after oral administration. Symptoms, signs and mode of death should be reported, with appropriate macroscopic and microscopic findings where necessary. LD 50s should be reported preferably with 95 percent confidence limits, if LD 50s cannot be determined, reasons for this should be stated.

3.2 Long term toxicity :—Long term toxicity (see Appendix I, item 4.3) should be carried out in at least two mammalian species, of which one should be a non-rodent. The duration of study will depend on whether the application is for marketing permission or for clinical trial, and in the latter case, on the phase of trials (see Appendix III). If a species is known to metabolize the drug in the same way as humans, it should be preferred.

In long term toxicity studies the drug should be administered 7 days a week by the route intended for clinical use in humans. The number of animals required for these studies, i.e. the minimum number on which data should be available, is shown in Appendix IV.

A control group of animals given the vehicle alone should always be included, and three other groups should be given graded doses of the drug; the highest dose should produce observable toxicity; the lowest dose should not cause observable toxicity, but should be comparable to the intended therapeutic dose in humans or a multiple of it. e.g. 2.5 to make allowance for the sensitivity of the species; the intermediate dose should cause some symptoms, but not gross toxicity or death, and may be placed logarithmically between the other two doses.

The variables to be monitored and recorded in long-term toxicity studies should include behavioural, physiological, biochemical, and microscopic observations.

3.3 Reproduction studies :—Reproduction studies (see Appendix I, item 4.4) need to be carried out only if the new drug is proposed to be studied or used in women of child bearing age. Two species should generally be used, one of them being a non rodent if possible.

(a) Fertility studies :—The drug should be administered to both males and females, beginning a sufficient number of days before mating. In females the medication should be continued after mating and the pregnant one should be treated throughout pregnancy. The highest dose used should not affect general health or growth of the animals. The route of administration should be the same as for therapeutic use in humans. The control and the treated group should be of similar size and large enough to give at least 20 pregnant animals in the control group of rodents and at least 8 pregnant animals in the control group of non-rodents. Observations should include total examination of the litters from both the groups, including spontaneous abortions, if any.

(b) Teratogenicity studies :—The drugs should be administered throughout the period of organogenesis, using three dose levels. One of the doses should cause minimum maternal toxicity and one should be the proposed dose for clinical use in humans or a multiple of it. The route of administration should be the same as for human therapeutic use. The control and the treated groups should consist of at least 20 pregnant females in case of non-rodents, on each dose used. Observations should include the number of implantation sites; resorptions if any; and the number of foetuses with their sexes, weights and malformations, if any.

(c) Perinatal studies :—The drug should be administered throughout the last third of pregnancy and then through lactation to weaning. The control of each treated group should have at least 12 pregnant females and the dose which causes low foetal loss should be continued throughout lactation weaning. Animals should be sacrificed and observations should include macroscopic, autopsy and where necessary histopathology.

3.4 Local toxicity :—These studies (see Appendix I, item 4.5) are required when the new drug is proposed to be used topically in humans. The drug should be applied to an appropriate site to determine local effects in a suitable species such as guinea-pigs or rabbits, if the drug is absorbed from the site of applications, appropriate systemic toxicity studies will be required.

3.5 Mutagenicity and Carcinogenicity :- These studies (see Appendix I, item 4.6) are required to be carried out if the drug or its metabolite is related to a known carcinogen or when the nature and action of the drug is such as to suggest a mutagenic|carcinogenic potential. For carcinogenicity studies, at least two species should be used. These species should not have a high incidence of spontaneous tumors and should preferably be known to metabolize the drug in the same manner as humans. At least three dose levels should be used; the highest dose should be sublethal but cause observable toxicity; the lowest dose should be comparable to the intended human therapeutic dose or a multiple of it, e.g. 2.5 ×; to make intermediate dose to be placed logarithmically between the other two doses. A control group should always be included. The drug should be administered 7 days a week or a fraction of the life span comparable to the fraction of human life span over which the drug is likely to be used therapeutically. Observations should include macroscopic changes observed at autopsy and detailed histopathology.

4. Animal pharmacology.

Specific pharmacological actions (see Appendix I, item 3.2) are those with therapeutic-potential for humans. These should be described according to the animal models and species used. Wherever possible, dose-response relationships and ED 50s should be given. Special studies to elucidate mode of action may also be described.

General pharmacological action (see Appendix I, item 3.3) are effects on other organs and systems, especially cardiovascular, respiratory and central nervous systems.

Pharmacokinetic data help relate drug effect to plasma concentration and should be given to the extent available.

5. Human|Clinical Pharmacology (Phase I).

The objective of phase I of trials (see Appendix I, item 5) is to determine the maximum tolerated dose in humans; pharmacodynamic effects; adverse reactions, if any, with their nature and intensity; and pharmacokinetic behaviour of the drug as far as possible. These studies are carried out in healthy adult males, using clinical, physiological and biochemical observations. At least 2 subjects should be used on each dose.

Phase I trials are usually carried out by investigators trained in clinical pharmacology and having the necessary facilities to closely observe and monitor the subjects. These may be carried out at one or two centres.

6. Exploratory trials (Phase II).

In phase II trial (see Appendix I, item 6) a limited number of patients are studied carefully to determine possible therapeutic uses, effective dose range and further evaluation of safety and pharmacokinetics. Normally 10-12 patients should be studied at each dose level. These studies are usually limited to 3-4 centres and are carried out by clinicians specialized in the concerned thrapeutic areas and having adequate facilities to perform the necessary investigations for efficacy and safety.

7. Confirmatory trials (Phase-III).

The purpose of these trials (see Appendix I, item 7) is to obtain sufficient evidence about the efficacy and safety of the drug in a larger number of patients, generally in comparison with a standard drug and or a placebo as appropriate. These trials may be carried out by clinicians in the concerned therapeutic areas, having facilities appropriate to the protocol. If the drug is already approved|marketed in other countries, phase III data should generally be obtained on at least 100 patients distributed over 3-4 centres primarily to confirm the efficacy and safety of the drug in Indian patients when used as recommended in the product monograph for the calims made.

If the drug is a new drug substance discovered in India, and not marketed in any other country, phase III data should be obtained on at least 500 patients distributed over 10-15 centres. In addition, data on adverse drug reactions observed during clinical use of the drug should be collected in 1000-2000 patients; such data may be collected through clinicians who give written consent to use the drug as recommended and to provide a report on its efficacy and adverse drug reactions in the treated patients. The selection of clinicians for such monitoring and supply of drug to them will need approval of the licensing authority under rule 21.

8. Special studies.

(A) These include studies on solid oral dosage forms, such as, bioavailability and disolution studies. These are required to be submitted on the formulations manufactured in the country. (See Appendix I, items 8.1 and 8.2).

(B) These include studies to explore additional aspects of the drug, e.g. use in elderly patients or patients with renal failure, secondary or ancillary effects, interactions, etc. (See Appendix I, items 8.1 and 8.2).

9. Submission of Reports (Appendix II).

The reports of completed clinical trials shall be submitted by the applicant duly signed by the investigator within a stipulated period of time. The

applicant, should do so even if he is no longer interested to market the drug in the country unless there are sufficient reasons for not doing so.

10. Regulatory status in other countries.

It is important to state if any restrictions have been placed on the use of the drug in any other country, e.g. dosage limits, exclusion of certain age groups, warnings about adverse drug reaction, etc. (See Appendix I, item 9.2).

Likewise, if the drug has been withdrawn from any country specially by a regulatory directive, such information should be furnished alongwith reasons and their relevance, if any, to India [See Appendix I, item 9.1 (d)].

11. Marketing information.

The product monograph should comprise the full prescribing information necessary to enable a physician to use the drug properly. It should include description, actions, indications, dosage precaution, drug interactions, warnings and adverse reactions.

The drafts of label and carton texts should comply with provisions of rules 96 and 97 of the said rules.

APPENDIX I

Data required to be submitted with application for permission to market a New Drug

1. INTRODUCTION

A brief description of the drug and the therapeutic class to which it belongs.

2. CHEMICAL AND PHARMACEUTICAL INFORMATION

2.1 Chemical name; code name or number, if any; non-proprietary or generic name, if any: structure; physiochemical proportion.

2.2 Dosage form and its composition.

2.3 Specifications of active and inactive ingredients.

2.4 Tests for identification of the active ingredient and method of its assay.

2.5 Outline of the method of manufacture of the active ingredient.

2.6 Stability data.

3. ANIMAL PHARMACOLOGY

3.1 Summary.

3.2 Specific pharmacological actions.

3.3 General pharmacological actions.

3.4 Pharmacokinetics: absorption: distribution: metabolism; excretion.

4. ANIMAL TOXICOLOGY (See Appendix IV & V).

4.1 Summary.

4.2 Acute Toxicity.

4.3 Long Term Toxicity.

4.4 Reproduction Studies.

4.5 Local Toxicity.

4.6 Mutagenicity and Carcinogenicity

5. HUMAN CLINICAL PHARMACOLOGY (PHASE I).

5.1 Summary.

5.2 Specific Pharmacological effects.

5.3 General Pharmacological effects.

5.4 Pharmacokinetics: absorption: distribution: metabolism; excretion.

6. EXPLORATORY CLINICAL TRIALS (PHASE II).

6.1 Summary.

6.2 Investigatorwise reports.

7. CONFIRMATORY CLINICAL TRIALS (PHASE III).

7.1 Summary.

7.2 Investigatorwise reports.

8. SPECIAL STUDIES

8.1 Summary.

8.2 Bioavailability and Dissolution studies.

8.3 Investigatorwise reports.

9. REGULATORY STATUS IN OTHER COUNTRIES.

9.1 Countries where —

(a) Marketed.

(b) Approved.

(c) Under trial, with phase.

(d) Withdrawn, if any, with reasons.

9.2 Restrictions on use, if any, in countries where marketed/approved.

9.3 Free sale certificate from country of origin.

10. MARKETING INFORMATION

10.1 Proposed product monograph.

10.2 Drafts of labels and cartons.

10.3 Sample of pure drug substance, with testing protocol.

Note :—(1) All items may not be applicable to all drugs, for explanation, see text of Schedule Y.

(2) For requirements of data to be submitted with application for clinical trials see text of Schedule Y, Section I and also Appendices II and III.

APPENDIX II

Format for submission of Clinical Trial Reports

- Title of the trial.
- Name of investigator and institution.
- Objectives of the trial.
- Design of study : Open, single-blind or double blind; non-comparative or comparative; parellel group or crossover.
- Number of patients, with criteria for selection and exclusion, whether written informed consent, was obtained.
- Treatments given—drugs and dosage forms; dosage regimens; method of allocation of patients to the treatments; method of verifying compliance, if any.
- Observations made before, during and at the end of treatment, for efficacy and safety, with methods used.
- Results : exclusions and dropouts, if any, with reasons; description of patients with initial comparability of groups where appropriate ; clinical and laboratory observations on efficacy and safety: adverse drug reactions.
- Discussion of results : relevance to objectives, corelation with other reports/data, if any; guidance for further study, if necessary.
- Summary and conclusion.

APPENDIX III

Animal toxicity requirements for clinical trials and marketing of a New Drug

Route of administration	Duration of Human administration	Phase	Long term toxicity requirements
1	2	3	4
	Single dose or several doses in one day	I-III MP	2 sp; 2 wk
Oral or Parenteral or Transdermal	Upto 2 wk	I, II	2 sp; Upto 4 wk
		III MP	2 sp; Upto 3 mo
		I, II	2 sp; 4 wk
	Upto 3 wk	III	2 sp; 3 mo
		MP	2 sp; Upto 6 mo
	Over 3 mo	I, II	2 sp; 3 mo
		III : MP	2 sp; 6 mo
Inhalation (general anaesthetics)		I : III MP	4 sp; 5 d (3h/d)
Aerosole	Repeated or Chronic use	I : II	1-2 sp; 3h/exp.
		III	1-2 sp; Upto 6 wk (2 exp/d)
		MP	1-2 sp; 24 wk (2 exp/d)

Dermal	Short term or Long term application	I : II	1 sp; single 24th exp; than 2 wk observation
		III : MP	1 sp; number and duration of applications commensurate with duration of use.
Ocular or Otic or Nasal	Single or Multiple applications	I : II	Irrigation test; graded doses
		I II	1 sp; 3 wk; daily applications as in clinical use.
		MP	1 sp; number and or duration of application commensurate with duration of use.
Vaginal or Rectal	Single or multiple application	I : II<br>III : MP	1 sp; number and duration of applications commensurate with duration of use.

Abbreviations : sp—species; wk—week; d—day; h—hour; mo—month; MP—Marketing Permission, exp—exposure.
I, II, III—Phases of clinical trial (see Appendix III, item No. 5-8).

Note :— (1) Animal toxicity data available from other countries are acceptable and do not need to be repeated/duplicated in India.

(2) Requirements for fixed dose combinations are given in Appendix VI.

APPENDIX—IV

Number of animals for long term Toxicity Studies

Group	2—6 Weeks				7—26 Weeks			
	Rodents (rats)		Non-Rodents (dogs)		Rodents (Rats)		Non-rodents (dogs)	
	M	F	M	F	M	F	M	F
Control	6—10	6—10	2—3	2—3	15—30	15—30	4—6	4—6
Low dose	6—10	6—10	2—3	2—3	15—30	15—30	4—6	4—6
Intermediate dose	6—10	6—10	2—3	2—3	15—30	15—30	4—6	4—6
High dose	6—10	6—10	2—3	2—3	15—30	15—30	4—6	4—6

APPENDIX V

Patient consent form for participation in a Phase I Clinical Trial

This clinical trial involves the study of a new ....agent............ in volunteers|patients suffering from ....

The drug which will be administered to volunteers|patients has been found to be safe in animal toxicity tests and other experimental data. The volunteers|patients will be required to undergo, if necessary, all routine examinations including taking of X-ray, ECG, EEG etc. at intervals. The volunteers|patients may be asked to collect stool and urine, and there may be need to draw blood or any other body fluid on several occasions to test the effects of concentrations of the drug. The volunteers patients are free to withdraw from the trial at any stage.

Authorisation

I have read|been briefed on the above project summary and I voluntarily agree to participate in the project. I understand that participation in this study may or may not benefit me. Its general purpose, potential benefits, possible hazards, and inconveniences have been explained to my satisfaction, I hereby give my consent for this treatment.

Name of the volunteer|patient

Signature or thumb|impression

of the volunteer|patient

Signature of Chief Investigator

Date :

Patient consent form for participation in Phase II and Phase III Clinical Trial :—

I .... exercising my free power of choice, hereby give my consent to be included as a subject in the clinical trial of a new drug, namely ....for the treatment of .... I understand that I may be treated with this drug for the diseases. I am suffering from .... I have been informed to my satisfaction, by the attending physician the purpose of the clinical trial and the nature of drug treatment and follow up including the laboratory investigation to monitor and safeguard my body functions.

I am also aware of my right to opt out of the trial at any time during the cause of the trial without having to give the reasons for doing so

Signature of the attending physician

Date :

Signature of patient

Date :

APPENDIX VI

Fixed dose Combinations (FDC) fall into four groups and their data requirements accordingly.

(a) The first group of FDC includes those in which one or more of the active ingredients is a new drug. Such FDC are treated in the same way as any other new drug, both for clinical trials and for marketing permission (see rule 122E, item (a)).

(b) The second group of FDC includes those in which active ingredients already approved|marketed individually are combined for the first time, for a particular claim and where the ingredients are likely to have significant interaction of a pharmacodynamic or pharmacokinetic nature (see rule 122E, item (c)). For permission to carry out clinical trials with such FDC, a summary of available pharmacological, toxicological and clinical data on the individual ingredients should be submitted, alongwith the rationale for combining them in the proposed ratio. In addition, acute toxicity data (LD 50) and pharmacological data should be submitted on the individual ingredients as well as their combination in the proposed ratio. If clinical trials have been carried out with the FDC in other countries, reports of such trials should be submitted. If the FDC is marketed abroad, the regulatory status in other countries should be stated. (See Appendix I, item 9).

For marketing permission, the reports of clinical trials carreid out with the FDC in India should be submitted. The nature of trials depending on the claims to be made and the data already available.

(c) The third group of FDC includes those which are already marketed, but in which it is proposed either to change the ratio of active ingredients or to make a new therapeutic claim.

For such FDC, the appropriate rationale should be submitted to obtain a permission for clinical trials, and the reports of trials should be submitted to obtain a marketing permission. The nature of trials will depend on the claims to be made and the data already available.

(d) The fourth group of FDC includes those whose individual active ingredients have been widely used in particular indication for years, their concomitent use is often necessary and no claim is proposed to be made other than convenience, and a stable acceptable dosage-form and the ingredients are unlikely to have significant interaction of a pharmacodynamic or pharmacokinetic nature.

No additional animal or human data are generally required for these FDC, and marketing permission may be granted if the FDC has an acceptable rationale."

[No. X-11011|1|87-DMS&PFA]

J. VASUDEVAN, Jt. Secy.

Note :—The Drugs and Cosmetics Rules, 1945 as amended upto 1-5-79 is contained in the publication of the Ministry of Health and Family Welfare (Department of Health) containing the Drugs and Cosmetics Act and the Rules (PDGHS-61). Subsequently the said rules have been amended by the following notification published in Part II, Section 3(i) of the Gazette of India, namely :—

1. GSR 1241 dated 6-10-1979.
2. GSR 1242 dated 6-10-1979.
3. GSR 1243 dated 6-10-1979.
4. GSR 1281 dated 12-10-1979.
5. GSR 430 dated 19-4-1980.
6. GSR 779 dated 26-7-1980.
7. GSR 540(E) dated 22-9-1980.
8. GSR 680(E) dated 5-12-1980.
9. GSR 681(E) dated 5-12-1980.
10. GSR 682(E) dated 5-12-1980.
11. GSR 27(E) dated 17-1-1981.
12. GSR 478(E) dated 6-8-1981.
13. GSR 62(E) dated 15-2-1982.
14. GSR 462(E) dated 22-6-1982.

15. GSR 510 (E) dated 22-7-1982.
16. GSR 13 (E) dated 7-1-1983.
17. GSR 318 (E) dated 1-5-1984.
18. GSR 331 (E) dated 8-5-1984.
19. GSR 460 (E) dated 2-6-1984.
20. GSR 487 (E) dated 2-7-1984.
21. GSR 89 (E) dated 16-2-1985.
22. GSR 788 (E) dated 10-10-1985.
23. GSR 17 (E) dated 7-1-1986.
24. GSR 1049(E) dated 29-8-1986.
25. GSR 1060 (E) dated 5-9-1986.
26. GSR 1115 (E) dated 30-9-1986.
27. GSR 71 (E) dated 30-1-1987.
28. GSR 570 (E) dated 12-6-1987.
29. GSR 626 (E) dated 2-7-1987.
30. GSR 792 (E) dated 17-9-1987.
31. GSR 371 (E) dated 24-3-1988.
32. GSR 675 (E) dated 2-6-88.
33. GSR 677 (E) dated 2-6-88
34. GSR 676(E) dated 2-6-88.
35. GSR 681 (E) dated 6-6-88.
36. GSR 735 (E) dated 24-6-88.
37. GSR 813 (E) dated 27-7-88.
38. GSR 854 (E) dated 12-8-88 (Corrigendum).

Printed by the Manager, Govt. of India Press, Ring Road, New Delhi-110064
and published by the Controller of Publications, Delhi-110054, 1988
