# Supplementary material for: Use of Fixed Dose Combination (FDC) Drugs in India: Central Regulatory Approval and Sales of FDCs Containing Non-Steroidal Anti-Inflammatory Drugs (NSAIDs), Metformin, or Psychotropic Drugs
Source: PLoS Med. 2015 May 12;12(5):e1001826. doi: 10.1371/journal.pmed.1001826 (PMC4428752; doi:10.1371/journal.pmed.1001826)
Supplement: S7 Text — (PDF) [file pmed.1001826.s010.pdf]

पाद टिप्पण :—1-5-1979 तक यथा संशोधित औषधि और प्रसाधन सामग्री नियम, 1945, स्वास्थ्य और परिवार कल्याण मंत्रालय (स्वास्थ्य विभाग) के औषधि और प्रसाधन सामग्री अधिनियम, 1940 एवं नियम संबंधी प्रकाशन में (पी.डी.जी.एच.एस.-61) में निहित है जिन्हें पिछली बार सा.का.नि. 395 (अ.) दिनांक 19-5-99 के तहत संशोधित किया गया था।

MINISTRY OF HEALTH AND FAMILY WELFARE

(Department of Health)

NOTIFICATION

New Delhi, the 17th August, 1999

G.S.R. 591(E).—Whereas a draft of certain rules further to amend the Drugs and Cosmetics Rules, 1945, was published as required by sections 12 and 33 of the Drugs and Cosmetics Act, 1940 (23 of 1940), under the notification of the Government of India in the Ministry of Health and Family Welfare (Department of Health) No. G.S.R. 383 (E), dated the 10th July, 1997, in the Gazette of India, Extraordinary, Part II, Section 3, Sub-section (i) dated the 10th July, 1997 inviting objections and suggestions from all persons likely to be affected thereby within a period of forty-five days from the date on which the copies of the Official Gazette containing the said notification were made available to the public ;

And whereas copies of the said Gazette were made available to the public on the 15th July, 1997 ;

And whereas the objections and suggestions received from the public on the said draft rules have been considered by the Central Government ;

Now, therefore, in exercise of the powers conferred by sections 12 and 33 of the said Act, the Central Government, after consultation with the Drugs Technical Advisory Board, hereby makes the following rules further to amend the Drugs and Cosmetics Rules, 1945, namely :—

1. (1) These rules may be called the Drugs and Cosmetics (Third Amendment) Rules, 1999.

(2) They shall come into force on the date of their publication in the Official Gazette.

2. In the Drugs and Cosmetics Rules, 1945, in rule 122(E), for clause (a), the following clause shall be substituted, namely:—

"(a) A drug, as defined in the Act including bulk drug substance which has not been used in the country to any significant extent under the conditions prescribed, recommended or suggested in the labelling thereof and has not been recognised as effective and safe by the licensing authority mentioned under rule 21 for the proposed claims :

Provided that the limited use, if any, has been with the permission of the licensing authority."

[No. X-11014/2/97-DMS & PFA]

DEEPAK GUPTA, Jt. Secy.

Foot Note :—The Drugs and Cosmetics Rules, 1945, as amended upto 1-5-1979 is contained in the publication of the Ministry of Health and Family Welfare (Department of Health) containing the Drugs and Cosmetics Act, 1940 and the Rules (PDGHS-61) last amended vide GSR 395 (E) dated 19-5-99.

Printed by the Manager, Govt. of India Press, Ring Road, Mayapuri, New Delhi-110064
and Published by the Controller of Publications, Delhi-110054.
